# Charitable donations and the theory of planned behaviour: A systematic review and meta-analysis

**DOI:** 10.1371/journal.pone.0286053

**Published:** 2023-05-19

**Authors:** Katherine M. White, Louise C. Starfelt Sutton, Xiang Zhao

**Affiliations:** 1 School of Psychology and Counselling, Queensland University of Technology, Brisbane, Australia; 2 Research and Evaluation Unit, Swedish Prison and Probation Service, Norrköping, Sweden; 3 School of Behavioural, Social and Legal Sciences, Örebro University, Örebro, Sweden; Caleb University, NIGERIA

## Abstract

Given the predominance of the theory of planned behaviour (TPB) to represent the psychological determinants underlying people’s charitable decisions, the present study synthesised the model’s key relationships, using meta-analysis, and tested the predictive utility of the model for charitable giving encompassing donations of blood, organs, time, and money. Given its relevance to altruistic decisions, the impact of moral norm was assessed also. A systematic literature review identified 117 samples (from 104 studies) examining donation intentions and/or prospective behaviour using TPB measures. The sample-weighted average effects for all associations were moderate-to-strong with perceived behavioural control (PBC) most strongly associated with intention (*r*+ = 0.562), followed by moral norm (*r*+ = 0.537), attitude (*r*+ = 0.507), and subjective norm (*r*+ = 0.472). Intention (*r*+ = 0.424) showed stronger associations with prospective behaviour than PBC (*r*+ = 0.301). The standard TPB predictors explained 44% of variance in intention (52% including moral norm). Intention and PBC explained 19% of variance in behaviour. A number of TPB associations showed differences when analysed for moderator variables such as length of follow-up for prospective behaviour and type of target behaviour. Stronger associations were found for the (subjective and moral) norm-intention associations among some of the different types of giving behaviours, especially for donating organs and time. Overall, the large proportion of variance explained by the TPB predictors especially for intention highlights those cognitions associated with people’s plans to give, informative for charities reliant on people’s propensity to give.

## Introduction

Globally, charitable giving satisfies important needs for individuals in the society including the provision of resources such as blood, organs, time, and money. According to the CAF World Giving Index 2021, while some forms of giving are on the rise (e.g., helping a stranger, donating money), others (e.g., volunteering time) have reduced [1]. Charitable organisations are dependent on people giving to promote initiatives that reduce poverty, advance education, aid health services, and increase societal benefit. In addition to assisting the recipients and charitable organisations, giving has been shown to benefit the donor also (e.g., physical and mental health benefits from donating time) [2,3]. Research on donation behaviour has established demographic determinants of charitable giving; for example, donors tend to be married, well-educated, and of an older age [4]. However, to promote public donations, charities benefit from an understanding of the psychological determinants of charitable donation behaviours.

While some authors view charitable giving as relating to financial donations [5], other approaches are broader in their terminology and include helping others and volunteering in addition to donating money [1]. For the scope of the present study, the review will focus on a broad definition of charitable giving as the voluntary donation of one’s personal resources (time, services, or assets/goods) to charity that provides benefits to others [6].

Researchers have often drawn on multidisciplinary approaches to understand giving. For instance, in their seminal work of people’s charitable financial donations, Bekkers and Wiepking [5] encompassed perspectives including economics, psychology, sociology, and biology and proposed eight mechanisms driving monetary donation (e.g., costs and benefits, altruism, values, and efficacy). Measures have been developed reflecting major underlying motives (e.g., trust, altruism, tax benefits) for donating money to charities [7]. In relation to predicting people’s giving behaviours, there is a number of proposed constructs and models to encapsulate the underlying psychological determinants of people’s giving decisions. Some commonly identified psychological constructs proposed to impact on people’s giving decisions include empathy [8], altruism [5], warm glow [9], social norms [10], and self/role identity [11]. For more comprehensive models of psychological determinants, examples include the Volunteers Function Index (VFI) developed by Clary and Snyder [12] to represent the underlying motivations people have for volunteering time. Other models of volunteering consider also process and structure characteristics and different types of volunteering, including the Volunteer Process Model and Episodic Volunteer Engagement and Retention Model [13,14]. Across some charitable giving behaviours (e.g., volunteering time, blood donation), distinctions are made for the purpose of conceptualising psychological determinants in different stages of the donation ‘career’, such as the initiation stage of giving. In the context of behaviours where a person’s willingness may be a more appropriate outcome than behavioural enactment (e.g., organ donation upon death), the Prototype Willingness Model [15] has been used to gauge people’s openness to give rather than actual donation.

One theoretical framework that has been examined across a wide variety of donation behaviours and is one of the major models applied in the field of psychological determinants of charitable giving is the Theory of Planned Behaviour. The Theory of Planned Behaviour [16] offers a parsimonious explanation of behaviour with intention conceptualised as the proximal determinant of people’s behavioural decisions. The determinants of intention are favourable/unfavourable evaluations of the behaviour (attitude), perceptions of normative pressure to perform the behaviour (subjective norm), and perceived ability/efficacy of performing the behaviour (perceived behavioural control; PBC). When PBC represents actual control over a target behaviour if it is not completely volitional, PBC directly influences behaviour. Attitude, subjective norm, and PBC are belief based with behavioural beliefs (advantages and disadvantages of behavioural performance) underlying attitude, normative beliefs (approval of specific referents for behavioural performance) underpinning subjective norm, and control beliefs (barriers and facilitators of behavioural performance) as the determinants of PBC.

With its inclusion of PBC, the TPB is an extension of the Theory of Reasoned Action (TRA) [17] which focused on the role of attitude and subjective norm on intention, and intention on behaviour. Despite criticisms of the TPB in the health domain [18] focusing on a lack of predictive validity of the standard TPB constructs and the lack of effectiveness of the TPB to provide practitioners with useful behavioural interventions which Ajzen [19] subsequently refuted, the model has garnered much evidence over time, with meta-analytic support across general behavioural domains [20,21].

The TPB is open to the inclusion of additional constructs provided they can be conceived as causal factors, add independent variance over and above the standard predictors, and can be applied to a range of behaviours in the interest of parsimony [16]. Arguably the most consistent addition in TPB examinations of charitable giving behaviour assesses a person’s sense of moral obligation to assist. Based on these and other findings in areas comprising a moral component (e.g., road safety and other decisions with legal and moral ramifications), researchers have argued for the inclusion of a moral/personal norm in the TPB [22]. Moral/personal norm reflects internalised rules or values with moral overtones [23] and engaging (or not engaging) in the target behaviour will, thus, result in self-approval or disapproval. Moral norm predicts additional variance in intention over and above the standard TPB predictors and is relevant across a range of behaviours involving an ethical/moral component [23,24]. The intention to donate blood is moderately associated with moral norm according to a previous meta-analysis [25]. As charitable giving results in significant consequences for the welfare and benefit of other people and groups of people, a sense of moral obligation is likely influential in decisions to engage in the behaviour. As it has been recognised independently as a useful addition to the TPB in previous meta-analytic studies, the construct of moral norm will be included also in this systematic synthesis.

Although a large body of literature has applied the TRA/TPB to understand charitable giving, there is a lack of a systematic synthesis and meta-analysis which enables evaluation of the consistency (or reasons for inconsistency) of results and provides more accurate estimates of TPB associations in this context. The only exception is a systematic review and meta-analysis of blood donation intention and behaviour; this study highlighted the role of attitude and PBC in blood donation intention and behaviour [25]. A review with a broader scope for donation behaviours is of interest to establish whether the strong associations identified in the review of blood donation are equally strong across other charitable giving domains (e.g., organs, money) and whether there is an overarching set of strong TPB associations shared across charitable behaviour types or stronger associations unique to one behavioural domain. Previous research has found high correlations between people giving both time and money [26,27] without as many examinations of whether the determinants of people’s giving across domains are of equal strength. Therefore, this review will examine one of the predominant social psychological models commonly employed to understand and predict people’s giving behaviours in the TPB. Of note, the model has been used by researchers to predict multiple charitable giving behaviours simultaneously [28].

Evaluating the contribution of more general decision-making models is this context is timely given critiques of the utility and validity of the TPB to represent adequately the variability in people’s behavioural decisions [18] and researchers’ efforts to propose models reflecting people’s decisions targeting specific donation behaviours [14]. Consequently, it is of benefit to researchers and practitioners alike to establish the extent to which parsimonious, general models of decision making such as the TPB are efficacious to represent the main determinants of people’s giving behaviours across a range of donation contexts. In this vein, clarity may be provided to identify whether there is sufficient evidence to support a general model of people’s charitable giving across distinct giving domains or if efforts are needed to continue to develop tailored predictive models unique to separate giving behaviours. Recognition of the key constructs of people’s giving behaviours allows practitioners from charitable organisations to focus recruitment and retention strategies on the most relevant underlying cognitions to promote giving intentions and behaviours.

The overall aim of this study, then, is to systematically review studies that have adopted a TPB approach to examine people’s giving intentions and behaviours and test the utility of the model as a parsimonious representation of the determinants of people’s giving decisions. The objectives of this systematic review of the charitable donation literature are to: (1) synthesise through meta-analysis TPB relationships, incorporating measures of moral/personal norm; (2) test the predictive utility of the TPB, with the addition of moral norm, within the context of charitable donations; and (3) explore relevant sample and methodological moderators of these associations. Specifically, seven moderators were considered. Sample characteristics of age, participant gender, and sample type (i.e., student status) were examined consistent with previous TPB meta-analyses examining sample-based differences [25,29]. These analyses remained exploratory in nature due to previously reported differences across types of donation behaviours. A potentially important methodological moderator to consider was the degree of specificity in the operationalisation of the target behaviour [30]. In the context of the TPB, the principles of Target, Action, Context, and Time (TACT) [31] were formulated to ensure alignment between the theoretical concept and measured outcome and to guide questionnaire construction. The principles promote specifying *who* performs the behaviour (Target), *what* the target behaviour is (Action), *where* or *how* the behaviour is performed (Context), and *when* the behaviour is performed (Time). In the current review, studies that adhered to the TACT principles were expected to render stronger TPB associations. Additional methodological considerations of attrition rates and follow-up lengths were investigated also, with higher attrition rates and shorter follow-up periods for measuring prospective behaviour expected to result in stronger associations. Finally, given the range of charitable donation behaviours covered, the type of donation behaviour was examined in an explorative manner.

## Methods

### Definition of the target behaviour

Charitable donations were, for the purpose of this study, defined as the voluntary donation of one’s personal resources (time, services, or assets/goods) to charity that provides benefits to others [6] without expectation of benefits in return [6]. Charities are not-for-profit/charitable organisations for public benefit.

### Search strategy and eligibility assessment

Searches for relevant literature were conducted in four stages. First, we performed database literature searches on January 9^th^ 2016 in PsycINFO (via EBSCOhost), PsycEXTRA (via EBSCOhost), Pub Med, Web of Science, ProQuest Dissertations & Thesis Global (abstract limiter), Scopus (title, abstract, keyword limiters), CINAHL (via EBSCOhost), Sociological Abstracts (via ProQuest), and EMBASE, using the following search string: (“theory of reasoned action” OR “theory of planned behaviour” OR “theory of planned behaviour”) AND (give OR giving OR donat* OR volunteer* OR charit* OR altruis* OR gift OR help*). The search terms were reviewed by an information specialist. Second, manual searches of reference lists of included records were conducted. Third, key authors (first/corresponding authors of included records) were contacted, and announcements were made through relevant professional associations (i.e., European Association of Experimental Social Psychology, Society for Personality and Social Psychology, Society of Australasian Social Psychologists) to source potential in press or unpublished studies. Finally, the database literature searches were repeated on April 16^th^ 2020 and March 18^th^ 2022 to source literature published since the initial search with additional data obtained at subsequent time points reviewed independently, retaining the partially processed data for the subsequent search iterations.

The identified records were screened for eligibility against four inclusion criteria: The record needed to: (1) report on a primary study; (2) use quantitative measurement of charitable donation intentions and/or prospective behaviour; (3) use the TPB as a basis for measurement; and (4) report bivariate correlations for at least one of the five basic TPB associations using the direct predictors (attitude-intention; subjective norm-intention; PBC-intention; intention-behaviour; PBC-behaviour). For intervention studies, relevant bivariate correlations from baseline or a non-intervention control group (e.g., wait-listed control) needed to be retrievable or obtainable from authors. To minimise publication bias, dissertations and unpublished work were included [32]. Although no language limiter was applied in the searches, articles reported in languages other than English were excluded.

To assess adherence of a record to the use of the TPB as a basis for measurement (inclusion criteria 3), we applied Ajzen’s [31] recommendations for constructing a TPB questionnaire as a guide. As such, we accepted affective (e.g., “[charitable giving] is pleasant-unpleasant”) and/or instrumental (e.g., “[charitable giving] is positive-negative”) measures of attitude; injunctive norms (e.g., “most people who are important to me would want me to [give to charity]”) and/or descriptive norms (e.g., “most people who are important to me [give to charity]”) as measures of subjective norm; and measures of self-efficacy (e.g., “I am capable of [charitable giving]”) and/or perceived control (“it is mostly up to me whether or not I [give to charity]”) to reflect PBC. For the normative component of the TPB, acceptable items could refer to specific referent groups (e.g., friends, family).

**Fig 1:**
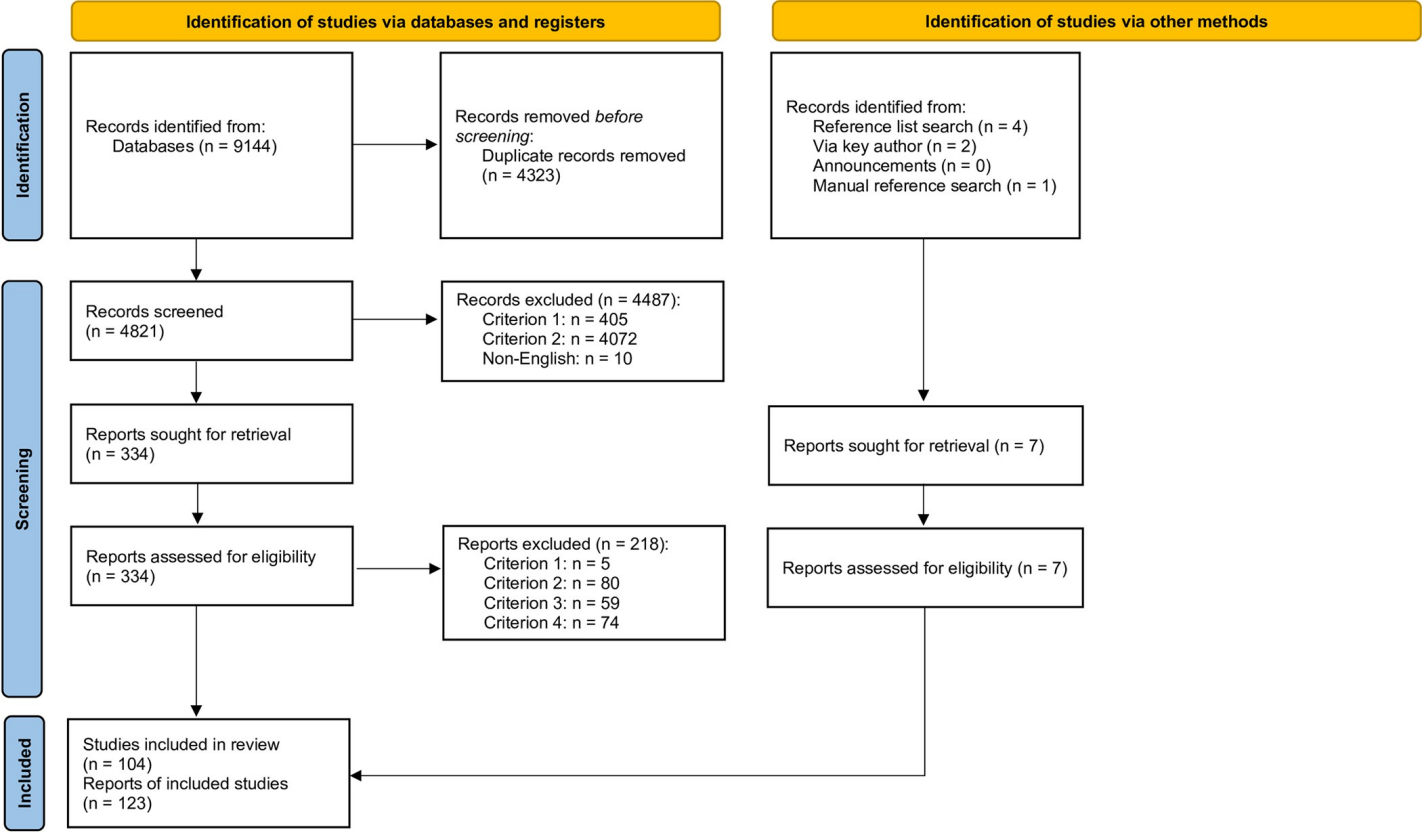
Flow diagram of the selection process. Criterion 1: Report on a primary study; Criterion 2: Use quantitative measurement of charitable donation intentions and/or prospective behaviour; Criterion 3: Use the TPB as a basis for measurement; Criterion 4: Report bivariate correlations for at least one of the five basic TPB associations using the direct predictors (attitude-intention; subjective norm-intention; PBC-intention; intention-behaviour; PBC-behaviour).

As shown in Fig 1, after removing duplicates, 4828 records were identified from database searches and additional search strategies. During an initial screening phase, one coder read titles and abstracts to assess eligibility based on inclusion criteria 1 and 2. A selection of 483 abstracts (10%, following the removal of duplicates) was screened also by a second coder to assess inter-coder reliability with a moderate-to-high level of agreement (Cohen’s κ = 0.92 in 2016, 0.74 in 2020, and 1.00 in 2022). The lower level of inter-rater reliability following the updated literature search in 2020 was due to more inclusive coding on part of the research assistant who completed the title and abstract screen, compared to the first and second author who conducted the double coding at the other time points, which was considered unproblematic given the subsequent full text screen rectified this over inclusion.

With title and abstract screening completed, 341 records (7.1%) remained. Records were most commonly excluded in the initial screening phase due to not measuring charitable donation intentions and/or prospective behaviour (criterion 2). In addition to exclusion based on stated criteria, 10 non-English articles were excluded at this stage. In a second screening phase, a full-text review was conducted using all four inclusion criteria to assess eligibility, which resulted in 123 included records. Records excluded at this stage most often did not include measurements of charitable donation intention and/or prospective behaviour (criterion 2). A random selection of 34 full-text records (10%) was reviewed by the first or second author and inter-coder reliability was high (Cohen’s κ = 0.88). Cases where disagreement occurred, and records which did not clearly meet inclusion criteria in the first and second phase of screening, were raised for discussion between all authors of the present study to reach a consensus decision. In cases where reporting was unclear or bivariate correlations were missing in studies which otherwise clearly met inclusion criteria, the study authors were contacted (up until the initial additional database search in April 2020). Risk of bias assessment was undertaken using the Joanna Briggs Institute Checklist for Analytical Cross Sectional Studies [33].

### Moderator coding

The included studies were independently coded by the first and second named authors to code relevant sample and methodological characteristics to enable tests of moderation (subgroup/meta-regression analyses). The *sample mean age* was extracted or calculated based on reported sample statistics. *Participant gender* was coded as the valid percentage of female participants in study samples. The coding of the *sample type* was based on whether the sampling strategy focused on students (e.g., participation for course credit) or the general population, with mixed sampling strategies categorised in the latter category. Ajzen’s [31] measurement principles of TACT were used to code definitional adherence: studies which specified the Target (e.g., participant), Action (e.g., donating blood) and Time (e.g., in the next three months) in the measurement of intention or prospective behaviour were coded as meeting the TACT criteria. Given that few studies specified the Context of charitable giving (e.g., a specific donation centre or blood drive), we did not apply this criterion in the moderator coding to enable group comparisons based on TACT adherence. In prospective studies assessing follow-up behaviour, the level of *attrition* was categorised as no attrition, or any attrition (between Time 1 and Time 2) and *follow-up time period* was coded as number of days follow-up between initial measurement and follow-up behaviour (reported ranges were averaged). Finally, to allow for a comparison of the results with a previous relevant meta-analysis of blood donation [25] and to explore the level of consistency of the strength of TPB associations across different types of charitable behaviours, the *target behaviour* was coded as donations of blood, organs, time, or money. Coding agreement between the first and second named authors was mixed (69.7–100%), with disagreement resolved successfully through discussion. The largest discrepancy (69.7% agreement for level of attrition) could be attributed to the differential coding of some studies reporting no attrition, erroneously coded as “not applicable”.

### Meta-analytic strategy and analyses

We followed the meta-analytic strategy reported in [32] and the PRISMA statement [34] for reporting (see S1 Checklist). The first/second named author completed data extraction which involved recording the full correlation matrix of TPB associations (including moral norm) and its associated sample size. In the case several correlation coefficients for a specific TPB construct (e.g., perceived control and self-efficacy) or for different charitable behaviour outcomes (e.g., blood and organ donation) were provided within the same sample, we used an average correlation to adhere to the assumption of independence. Correlations that were reported separately for multiple samples in a single study were treated independently (see Table 1).

**Table 1:** Overall sample-weighted effect sizes and heterogeneity analysis for each TPB association.

TPB association	*N*	k	*r*+	95% CI	Q	df	*p*	*I* ^2^
Attitude-Intention	55567	112	.507	.473;.539	2848.37	111	< .001	96.10
Subjective norm-Intention	51182	100	.472	.420;.520	4990.28	99	< .001	98.02
PBC-Intention	48489	91	.562	.513;.608	4982.35	90	< .001	98.19
Moral norm-Intention	28135	46	.537	.480;.590	1544.31	45	< .001	97.09
Intention-Behaviour	11543	41	.424	.367;.477	459.12	40	< .001	91.29
PBC-Behaviour	7927	26	.301	.227;.370	281.39	25	< .001	91.12
Attitude-Subjective norm	48592	90	.378	.342;.413	1694.31	89	< .001	94.75
Attitude-PBC	47227	86	.394	.350;.437	2431.32	85	< .001	96.50
Attitude-Behaviour	10554	33	.205	.161;.249	151.63	32	< .001	78.90
Attitude-Moral norm	28063	46	.390	.335;.442	986.10	45	< .001	95.44
Subjective norm-PBC	47732	87	.350	.283;.414	5367.24	86	< .001	98.40
Subjective norm-Behaviour	8107	26	.212	.163;.259	111.78	25	< .001	77.64
Subjective norm-Moral norm	26343	43	.458	.415;.499	583.85	42	< .001	92.81
PBC-Moral norm	25104	39	.319	.262;.373	676.79	38	< .001	94.39
Moral norm-Behaviour	4881	16	.244	.172;.314	95.81	15	< .001	84.34

*Note*: *N* = Total sample

k = number of samples/associations

*r*+ = Sample-weighted correlation coefficients

CI = Confidence Interval

Q = Cochran’s Q

df = Degrees of freedom

*p* = probability level

*I*^2^ = estimated percentage of true variance among population effect sizes.

Sample-weighted correlation coefficients (*r*+) were calculated using Comprehensive Meta-Analysis version 3.3.070 [35]. With a broad definition of charitable behaviour, we applied a random effects model to allow for effect size heterogeneity [36,37]. Sample-weighted correlations were labelled small (.10), medium (.30), or strong (.50) in line with guidelines [38]. Regression analysis was undertaken in SPSS using the sample-weighted correlations in an input matrix. Subgroup analyses to test for moderation were conducted when k within subgroups exceeded 3. Based on untransformed (continuous) moderators, sample size-weighted regression analyses were first conducted. As linearity tests indicated spurious nonlinear relationships between the moderators and intervariable correlations, dichotomised recodification was applied for continuous moderators (see Table 2 for categorisation of these moderators). For transparency, moderation results based on continuous moderators are provided in Table 3. Homogeneity analyses were conducted to identify dispersion among effect sizes, with a significant Q statistic denoting heterogeneity and *I*^2^ indicating the magnitude of true variance among population effect sizes. Funnel plots in combination with [39] trim and fill analysis were used to assess publication bias. The risk of bias was assessed formally by the Joanna Briggs Institute Checklist for Analytical Cross Sectional Studies [40] given the predominance of included studies were neither longitudinal nor experimental. Some criteria were used around specific questions to increase the fairness in judging their bias potential. The first author reviewed all studies while the second author assessed 10% of included studies with a limited number of inconsistencies discussed between the authors.

**Table 2:** Subgroup analyses based on sampling, methodological, and behaviour type moderators.

TPB association	Moderator	Subgroup	Within-group statistics						Between-groups statistics			
			k	*r*+	95% CI	Q	df	*p*	*I* ^2^	Q	df	*p*
Attitude-Intention	Sample age	Young	38	.571	.519;.619	580.21	37	< .001	93.62	12.24	1	< .001
		Older	57	.450	.404;.493	1344.39	56	< .001	95.84			
	Sample gender	Male	32	.473	.403;.543	698.77	31	< .001	95.56	1.52	1	.217
		Female	71	.523	.481;.562	1891.82	70	< .001	96.30			
	Sampling	Student	39	.574	.529;.615	423.47	38	< .001	91.03	13.19	1	< .001
		Non-student	69	.459	.414;.501	1961.83	68	< .001	96.53			
	TACT	Yes	62	.460	.410;.507	1674.53	61	< .001	96.36	9.84	1	< .001
		No	50	.562	.519;.602	911.02	49	< .001	94.62			
	Behaviour	Blood donation	50	.475	.428;.520	1184.94	49	< .001	95.87	3.06	1	.080
		Other	62	.532	.487;.575	1270.92	61	< .001	95.20			
Subjective norm-Intention	Sample age	Young	39	.473	.429;.515	342.51	38	< .001	88.91	0.17	1	.680
		Older	49	.493	.405;.572	4463.74	48	< .001	98.93			
	Sample gender	Male	24	.508	.435;.574	525.90	23	< .001	95.63	0.84	1	.361
		Female	70	.463	.393;.528	4242.29	69	< .001	98.37			
	Sampling	Student	36	.452	.404;.497	290.63	35	< .001	87.96	0.85	1	.357
		Non-student	60	.492	.418;.559	4663.24	59	< .001	98.74			
	TACT	Yes	57	.440	.389;.489	1371.92	56	< .001	95.92	1.830	1	.176
		No	43	.511	.419;.592	2884.66	42	< .001	98.54			
	Behaviour	Blood donation	43	.384	.336;.429	691.82	42	< .001	93.93	13.70	1	< .001
		Other	59	.533	.470;.590	2389.77	58	< .001	97.57			
PBC-Intention	Sample age	Young	34	.578	.494;.630	888.72	33	< .001	96.29	0.07	1	.796
		Older	49	.566	.508;.641	3618.45	48	< .001	98.67			
	Sample gender	Male	22	.579	.474;.667	1091.80	21	< .001	98.08	0.08	1	.783
		Female	63	.562	.499;.620	3763.01	62	< .001	98.35			
	Sampling	Student	29	.509	.428;.582	617.69	28	< .001	95.47	2.78	1	.095
		Non-student	58	.590	.530;.644	3793.12	57	< .001	98.50			
	TACT	Yes	54	.573	.522;.620	1783.42	53	< .001	97.03	0.24	1	.626
		No	37	.546	.440;.637	3198.75	36	< .001	98.88			
	Behaviour	Blood donation	42	.573	.524;.619	1164.71	41	< .001	96.48	0.04	1	.833
		Other	51	.563	.474;.641	3868.15	50	< .001	98.71			
Moral norm-Intention	Sample age	Young	17	.549	.478;.612	218.29	16	< .001	92.67	0.14	1	.712
		Older	29	.530	.455;.598	1097.16	28	< .001	97.45			
	Sample gender	Male	13	.514	.402;.610	214.49	12	< .001	94.41	0.23	1	.635
		Female	34	.543	.474;.606	1259.82	33	< .001	97.38			
	Sampling	Student	14	.584	.488;.667	224.20	13	< .001	94.20	1.36	1	.243
		Non-student	31	.517	.448;.580	1077.69	30	< .001	97.22			
	TACT	Yes	32	.555	.481;.622	1281.48	31	< .001	97.58	0.95	1	.330
		No	14	.498	.399;.585	253.93	13	< .001	94.88			
	Behaviour	Blood donation	23	.442	.377;.502	540.91	22	< .001	95.93	13.19	1	< .001
		Other	25	.613	.545;.673	372.50	24	< .001	93.56			
Intention-Behaviour	Sample age	Young	8	.400	.256;.526	82.18	7	< .001	91.48	0.37	1	.543
		Older	26	.447	.370;.519	319.78	25	< .001	92.18			
	Sample gender	Male	18	.398	.309;.480	179.04	17	< .001	90.51	0.62	1	.431
		Female	20	.446	.358;.527	266.80	19	< .001	92.88			
	Sampling	Student	9	.464	.336;.576	74.50	8	< .001	89.26	0.40	1	.527
		Non-student	31	.420	.357;.480	329.21	30	< .001	90.89			
	TACT	Yes	32	.442	.378;.501	412.19	31	< .001	92.48	1.47	1	.225
		No	9	.366	.254;.469	36.56	8	< .001	78.12			
	Behaviour	Blood donation	19	.385	.315;.452	214.12	18	< .001	91.59	1.61	1	.205
		Other	21	.461	.363;.549	218.35	20	< .001	90.84			
	Attrition	No	17	.329	.263;.392	109.30	16	< .001	85.36	7.08	1	.008
		Any	21	.483	.390;.566	285.59	20	< .001	93.00			
	Follow-up	Short	20	.526	.439;.603	225.58	19	< .001	91.58	12.53	1	< .001
		Long	20	.325	.254;.392	175.38	19	< .001	89.17			
PBC-Behaviour	Sample age	Young	6	.266	.141;.382	32.19	5	< .001	84.47	0.64	1	.423
		Older	15	.334	.215;.443	261.19	14	< .001	93.52			
	Sample gender	Male	9	.204	.143;.264	25.46	8	< .001	68.58	4.59	1	.032
		Female	15	.364	.231;.483	231.61	14	< .001	93.96			
	Sampling	Student	5	.298	.113;.463	27.15	4	< .001	85.27	0.02	1	.881
		Non-student	20	.313	.229;.392	223.60	19	< .001	91.50			
	TACT	Yes	21	.326	.239;.408	275.67	20	< .001	92.75	4.98	1	.026
		No	5	.209	.153;.264	1.74	4	.783	0.00			
	Behaviour	Blood donation	13	.284	.195;.368	118.77	12	< .001	89.90	0.34	1	.558
		Other	12	.331	.195;.456	151.61	11	< .001	92.74			
	Attrition	No	7	.229	.195;.262	29.62	6	< .001	79.75	2.14	1	.144
		Any	17	.276	.249;.303	226.72	16	< .001	92.94			
	Follow-up	Short	13	.389	.269;.498	143.56	12	< .001	91.64	5.03	1	.025
		Long	12	.222	.136;.305	93.85	11	< .001	88.28			

*Note*: k = number of samples/associations, *r*+ = Sample-weighted correlation coefficients, CI = Confidence Interval, Q = Cochran’s Q, df = Degrees of freedom, *p* = probability level, *I*^2^ = estimated percentage of true variance among population effect sizes. TACT = Target, Action, Context, Time.

**Table 3:** Subgroup analysis based on tetradic behaviour type moderators.

TPB association	Reference type	Behaviour type	Coefficient	*p*
Attitude-Intention Q_(3)_ = 21.07, *p* < .001	Blood	Organs	0.20	< .001
		Time	−0.06	.293
		Money	0.15	.025
	Time	Blood	0.06	.293
		Organs	0.26	< .001
		Money	0.21	.006
	Money	Blood	−0.15	.025
		Organs	0.05	.513
		Time	−0.21	.006
Subjective norm-Intention Q_(3)_ = 11.57, *p* = .009	Blood	Organs	0.21	.005
		Time	0.17	.014
		Money	0.16	.080
	Time	Blood	−0.17	.014
		Organs	0.04	.638
		Money	−0.00	.971
	Money	Blood	−0.16	.080
		Organs	0.04	.685
		Time	0.00	.971
PBC-Intention Q_(3)_ = 1.12, *p* = .773	Blood	Organs	−0.01	.335
		Time	0.03	.711
		Money	0.05	.678
	Time	Blood	−0.03	.711
		Organs	−0.13	.249
		Money	0.02	.896
	Money	Blood	−0.05	.678
		Organs	−0.15	.293
		Time	−0.02	.896
Moral norm-Intention Q_(3)_ = 25.21, *p* < .001	Blood	Organs	0.41	< .001
		Time	0.27	.002
		Money	0.18	.032
	Time	Blood	−0.27	.002
		Organs	0.15	.177
		Money	−0.09	.424
	Money	Blood	−0.18	.032
		Organs	0.23	.030
		Time	0.09	.424
Intention-Behaviour Q_(3)_ = 11.25, *p* = .011	Blood	Organs	0.03	.825
		Time	0.22	.008
		Money	-0.15	.195
	Time	Blood	-0.22	.008
		Organs	-0.19	.136
		Money	-0.37	.003
	Money	Blood	0.15	.195
		Organs	0.17	.250
		Time	0.37	.003
PBC-Behaviour Q_(3)_ = 1.60, *p* = .660	Blood	Organs	-0.03	.829
		Time	0.12	.269
		Money	-0.02	.884
	Time	Blood	-0.12	.269
		Organs	-0.15	.345
		Money	-0.14	.369
	Money	Blood	0.02	.884
		Organs	-0.01	.953
		Time	0.14	.369

*Note*: Q = Cochran’s Q.

## Results

### Study characteristics and risk of bias assessment

Eligibility screening resulted in 123 included records, reporting on 104 studies with 117 independent samples. Data (available from the Open Science Framework https://osf.io/dkptj/) were obtained from 27 countries, most commonly USA, over a 44-year time period between 1978 and 2022. Data extraction was undertaken from 94 articles published in peer-reviewed journals, 2 book chapters, 9 conference abstracts (in combination with published articles and/or information from authors), 1 unpublished report, and 17 dissertations. Sample sizes in individual studies ranged between 51 and 12,051 participants. Examples of charitable behaviours examined in the studies are blood donation, organ donation, volunteering for community services, and monetary donations to charities. More detailed information about the included studies can be found in S1 Table. Please note some studies were reported in multiple records (e.g., a conference abstract and a published article reporting the same study).

Moderator coding (see S2 Table) revealed the sample mean age varied between 18.94 and 66.35 years. With dichotomous coding of the age moderator, there were 39 unique young samples and 59 older samples (18 undetermined and 1 sample coded as “older” at baseline and “young” at follow-up). The percentage of female participants in the study samples varied between 0% (all male participants) and 100% (all female participants), with most studies sampling more female than male participants (> 50%) (11 undetermined). Applying dichotomous coding, 76 samples consisted of mainly female participants and 33 of mainly male participants (8 undetermined). Study recruitment targeted students in 42 samples and non-students, or a mix of students and general population members, in 73 samples (2 undetermined). The TACT principles were applied in 59 (56.7%) of 104 studies. In the prospective studies predicting behaviour, the level of attrition ranged from 0% and 70.5% and the length of follow-up time period in prospective studies was 0 days (immediate follow-up) to 2 years. Level of attrition (0% vs. any attrition) was categorised as no attrition in 19 cases and any attrition in 24 cases (3 undetermined) to represent the time between the first survey of TPB predictors and the follow-up measure of behaviour. Based on a median split of 2 months for length of follow-up in prospective studies, 22 cases were categorised as a short time period and 24 cases were classified as a long time period. Blood donation intention or behaviour was the main target behaviour, examined exclusively in 50 (42.7%) of 117 samples.

For the risk of bias assessment, most of the included studies met criteria to be considered as generally effective in addressing bias in their design, conduct, and analysis. A small number of studies did not provide clear inclusion criteria for participants, most studies provided study participant details of age and sex demographics without further descriptors (e.g., ethnicity, socio-economic status), and many studies described the location (but not often the time period) for study setting information. Further, a minority of studies did not refer to scale reliability or the reliability was low for some measures. Nearly all of the included studies were rated as using appropriate statistical analysis. Few studies used objective measures of behaviour (if assessing behaviour prospectively) but most studies included information about how the non-objective measures were validated. The criteria related to diagnoses and confounding factors/associated strategies in relation to comparison groups were not applicable in nearly all of the identified studies (see S3 Table).

### Sample-weighted effect sizes and publication bias assessment

The sample-weighted correlations (see Table 1) showed that all theoretically relevant TPB associations were moderate-to-strong. The strongest association with charitable giving intention was PBC (*r*+ = 0.562, 95% CI [0.513; 0.608], *p* < .001), followed by moral norm (*r*+ = 0.537, 95% CI [0.480; 0.590], *p* < .001), attitude (*r*+ = 0.507, 95% CI [0.473; 0.539], *p* < .001), and subjective norm (*r*+ = 0.472, 95% CI [0.420; 0.520], *p* < .001). Intention showed a stronger association with prospective behaviour (*r*+ = 0.424, 95% CI [0.367; 0.477], *p* < .001) compared to PBC (*r*+ = 0.301, 95% CI [0.227; 0.370], *p* < .001). Forest plots for each TPB association showed considerable heterogeneities among the correlations (see S1 to S6 Figs in S1 File), with a statistically significant Q statistic for each association (see Table 1). The range of the true effect size dispersion among the six main associations was from 91.12% for PBC-behaviour and 98.19% for PBC-intention, supporting the need to undertake moderator analyses.

For the six TPB associations, there was no obvious indication of asymmetry as confirmed by the funnel plots (see S7 Fig in S1 File). An assessment of publication bias via Duval and Tweedie’s trim-and-fill analyses only revealed a minimal risk for publication bias across the six TPB associations. Therefore, it was concluded the data revealed no evidence of publication bias, without any need of adjustment for overall effect sizes.

### Regression analysis

The sample-weighted correlations were used to create an input matrix for a regression analysis to examine the utility of the TPB in explaining charitable donation intentions (*N* = 47227) and prospective behaviour (*N* = 7927). The combination of attitude, subjective norm, and PBC explained 44.2% of variance in intention. A difference was observed in the predictive strength of attitude (*B* = .23), subjective norm (*B* = .25), and PBC (*B* = .39), with PBC as the strongest predictor. When moral/personal norm was incorporated with the standard TPB predictors for intention, the combination of standard predictors and moral norm explained 51.6% of variance in intentions. PBC remained the strongest predictor (*B* = .34), followed by moral/personal norm (*B* = .28), attitude (*B* = .21), and subjective norm (*B* = .15). The combination of intention and PBC explained 18.5% of variance in prospective behaviour, with intention the main predictor (*B* = .37) compared to PBC (*B* = .09).

### Moderator analyses

Moderator analyses were conducted for the sample (age, participant gender, sample type/student status) and methodological (TA(C)T) adherence, attrition, length of follow-up) characteristics, as well as target behaviour type (blood donation vs. organ donation vs. volunteering time vs. donating money). Subgroup analyses were completed for associations where the number of independent effect sizes per subgroup exceeded 3. Analyses assessing the moderators comprising sample characteristics (age, gender, student status), adherence to TA(C)T principles as a methodological moderator, as well as target behaviour type, were undertaken for all TPB associations. Analyses assessing the moderators reflecting the methodological characteristics of attrition and length of follow-up were conducted only for the intention-behaviour and PBC-behaviour associations of those studies assessing prospective behaviour (see Table 2 for the results of moderator analyses including within and between subgroup statistics).

Subgroup comparisons for the sample characteristic of participant age group (young, older) were possible for all TPB associations examined. The attitude-intention association was stronger in young samples (k = 38, *r*+ = 0.571, 95% CI [0.519; 0.619]) compared to older samples (k = 57, *r*+ = 0.450, 95% CI [0.404; 0.493]), Q = 12.24, df = 1, *p* < .001. There was no significant moderation for any of the remaining TPB associations. Significant heterogeneity remained in all subgroups.

For the sample characteristic of participant gender (majority male participants, majority female participants), subgroup analyses were possible for all TPB associations examined. The PBC-behaviour association was stronger when the samples consisted of mainly female participants (k = 15, *r*+ = 0.364, 95% CI [0.231; 0.483]) compared to male participants (k = 9, *r*+ = 0.204, 95% CI [0.143; 0.264]), Q = 4.59, df = 1, *p* = 0.032. There was no significant moderation for any of the other TPB associations. Significant heterogeneity remained in all subgroups.

Subgroup analyses were possible for all of the six TPB associations for sample type (student, non-student/mixed). Comparisons revealed student samples had a statistically significantly larger sample-weighted effect size (k = 39, *r*+ = 0.574, 95% CI [0.529; 0.615]) than non-student samples (k = 69, *r*+ = 0.459, 95% CI [0.414; 0.501]) for the attitude-intention association, Q = 13.19, df = 1, *p* < 0.001. No other significant differences were found. Significant heterogeneity remained among all subgroups.

For the methodological moderator of use of TA(C)T principles (yes, no), subgroup analyses were possible for all TPB associations. A statistically significant difference in the sample-weighted effect sizes was found for the attitude-intention association (Q = 9.84, df = 1, *p* < .001). Contrary to expectations however, the association was weaker when the intention measure adhered to TA(C)T (k = 62, *r*+ = 0.460, 95% CI [0.410; 0.507]) compared to not (k = 50, *r*+ = 0.562, 95% CI [0.519; 0.602]). The PBC-behaviour association was, as expected, statistically significantly stronger when TA(C)T was applied (k = 21, *r*+ = 0.326, 95% CI [0.239; 0.408]) compared to not (k = 5, *r*+ = 0.209, 95% CI [0.153; 0.264]), Q = 4.98, df = 1, *p* = 0.026. The difference in the sample-weighted effect size between subgroups was similarly large and in the expected direction for the intention-behaviour association; however, the moderation effect did not reach statistical significance. Correlations for the PBC-behaviour association when the behaviour measure did not adhere to TA(C)T were homogenous as indicated by the Q statistic; however, this finding should be interpreted with caution due to the small number of independent effect sizes (k = 5). Heterogeneity remained within all other subgroups.

Moderator analyses were conducted for methodological characteristics of level of attrition and follow-up time period for the PBC-behaviour and intention-behaviour associations in studies with a prospective measure of behaviour. The intention-behaviour association was significantly stronger with any (k = 21, *r*+ = 0.483, 95% CI [0.390; 0.566]) compared to no attrition (k = 17, *r*+ = 0.329, 95% CI [0.263; 0.392]), Q = 7.08, df = 1, *p* = 0.008. For the PBC-behaviour association, the difference in sample-weighted correlations as a function of attrition was large and in the expected direction but the moderation effect did not reach statistical significance. Significant heterogeneity remained in all subgroups.

The PBC-behaviour association was statistically significantly larger when the length of follow-up was short (k = 13, *r*+ = 0.389, 95% CI [0.269; 0.498]) compared to long (k = 12, *r*+ = 0.222, 95% CI [0.136; 0.305]), Q = 5.03, df = 1, *p* = 0.025. Similarly, the intention-behaviour association was significantly stronger when the follow-up time period was short (k = 20, *r*+ = 0.526, 95% CI [0.439; 0.603]) compared to long (k = 20, *r*+ = 0.325, 95% CI [0.254; 0.392]), Q = 12.53, df = 1, *p* < 0.001. Significant heterogeneity remained in all subgroups.

Subgroup analyses were conducted for all TPB associations for the type of target behaviour (blood donation vs. other types of donation). The analyses revealed significant moderation for the subjective norm-intention (Q = 13.79, df = 1, *p* < .001) and moral norm-intention (Q = 13.19, df = 1, *p* < .001) associations. The subjective norm-intention correlation was weaker when the target behaviour was blood donation (k = 43, *r*+ = 0.384, 95% CI [0.336; 0.429]) compared to other types of charitable donations (k = 59, *r*+ = 0.533, 95% CI [0.470; 0.590]). Similarly, the moral norm-intention association was weaker for blood donation behaviour (k = 23, *r*+ = 0.442, 95% CI [0.377; 0.502]) compared to other types of behaviours (k = 25, *r*+ = 0.613, 95% CI [0.545; 0.673]). Significant heterogeneity remained in all subgroups.

As shown in Table 3, TPB associations among the four specific giving behaviours were compared. Consistent with the previous results (see Table 2), moderation for the PBC-intention (Q = 1.12, df = 3, *p* = 0.773) and PBC-behaviour (Q = 1.60, df = 3, *p* = 0.660) was not significant. However, giving behaviour type showed a significant moderation effect on the other four associations. Compared with blood donation, the attitude-intention (b = 0.20, *p* < .001), subjective norm-intention (b = 0.21, *p* = 0.005), and moral norm-intention (b = 0.41, *p* < 0.001) associations were stronger for organ donation; the subjective norm-intention (b = 0.17, *p* = 0.014), moral norm-intention (b = 0.27, *p* = 0.002), and intention-behaviour (b = 0.22, *p* = 0.008) associations were stronger for donating time; and the attitude-intention (b = 0.15, *p* = 0.025) and moral norm-intention (b = 0.18, *p* = 0.032) associations were stronger for money donation. Compared with time donation, the attitude-intention (b = 0.26, *p* < 0.001) association was stronger for organ donation; and a weaker intention-behaviour (b = -0.37, *p* = 0.003) association and a stronger attitude-intention (b = 0.21, *p* = 0.006) association were found for money donation. Compared with money donation, the moral norm-intention (b = 0.23, *p* = 0.030) association for organ donation was stronger.

## Discussion

In an effort to establish the validity of general decision-making models to represent the cognitions contributing to deliberative helping actions across a range of giving contexts, the present study assessed the utility of the Theory of Planned Behaviour (TPB), with the addition of moral norm, in reflecting the psychological determinants of people’s charitable donation decisions. Across 117 samples, strong support was found for the model in predicting people’s intention to donate across a broad range of charitable giving behaviours, with a moderate amount of variance explained for predicting people’s reported donation behaviours. Effect sizes across studies were consistently positive with few exceptions and summary effects were moderate-to-strong for all expected TPB associations. In particular, PBC and moral norm featured as strong determinants of people’s charitable intentions, with intention the strongest proximal determinant of behaviour. There were generally stronger associations for charitable intentions when prospective behaviour was measured at a short follow-up period. For specific behaviour types, differences included the (subjective and moral) norm-intention and attitude-intention links as weaker for blood than other forms of donation. Overall, the findings suggest a generic decision-making model is an efficient and parsimonious representation of especially people’s giving intentions, with minor variability in the strength of the major pathways of the model across different giving actions predominantly related to the impact of norms.

The amount of variance the standard TPB predictors explained in predicting intention (44%) exceeds meta-analytic results in general (e.g., 39%) [20]. Predicting donation behaviour from the standard TPB constructs (19%), however, fell somewhat short of expectations based on general TPB meta-analytic findings of 27% [20]. As a behavioural domain, then, donation as a behaviour may be more subject to the ‘intention-behaviour gap’ [41] than other types of behaviour (e.g., health behaviours), with self-regulatory tools such as implementation intentions (making if-then plans) potentially required to improve the likelihood that charitable giving intentions will translate into donation behaviours. With all summary effects moderate-to-strong in the present study, comparisons to the more general meta-analysis of Armitage and Conner show similar patterns for the strength of the predictors of behaviour, but with stronger relationships for the association between both subjective norm and PBC with intention in the case of people’s donation behaviours. Comparison to Bednall and Bove’s [25] meta-analysis of TPB blood donation studies shows similar associations for the predictors of behaviour but larger values for the association between both subjective norm and moral norm with intention, consistent with the moderator analyses between blood donation and other types of giving in the present study.

The types of studies included in the review favoured blood donation rather than the other forms of donation, with a few study examples of specific types of blood donation (i.e., plasma, cord) reflecting the changing foci of blood collection agencies in some jurisdictions. There was a predominance of studies examining donating money over goods. Of note, most of the settings for the included studies were developed nations, with developing countries underrepresented. While a few studies examined volunteering in general, most referenced volunteering in a specific context (e.g., at a homeless shelter) with a couple of studies focusing on online options. While only a minority of the studies included a follow-up assessment of donation behaviour, the studies examining organ donation were very unlikely to include a subsequent assessment of behaviour given the nature of the behaviour and the delay in action for most people to register their organ donation preference. It is noteworthy there was a mix of studies focusing exclusively on students as opposed to community samples in general. Very few studies assessed multiple donation behaviours simultaneously, prohibiting the opportunity to examine if there is consistency in people’s donation actions and determinants across the differing types of giving opportunities.

The dominant role of control perceptions determining donation intentions across donation types points to the central role of feeling capable to be able to perform altruistic actions. The moral dimension of altruistic actions remains paramount and confirms support for inclusion of moral norm as a critical addition to the TPB when assisting others is targeted. The lesser but significant role for attitudes and subjective norm confirms continued support for the original model components but indicates a sense of morality is likely more important to consider when evaluating relevant conceptual contributions. Intention remains a key consideration for predicting people’s subsequent donation behaviour, highlighting the planned nature of many of the altruistic actions people choose to perform. However, the review’s findings suggest greater conceptual work is needed to accurately reflect the determinants of people’s actual donation decisions, as opposed to their intentions to give.

Importantly, the results of the present study may serve to inform strategies for charities and not-for-profit organisations in promoting people’s engagement in donation behaviours. Given its dominant role in predicting donation behaviours, a focus on the antecedents of people’s intentions may be worthwhile in this context. Broadly speaking, strengthening intention via a focus on both constructs of PBC and moral norm may be associated with higher intentions to donate. For instance, strategic considerations of instilling confidence that acts of giving are easy to do and describing donation as deeds one ‘should’ perform may be useful.

### Proposed moderators

Moderators based on sample characteristics were examined and differences were minimal. There was a statistically significant difference for age and sample type (student status) between attitude and intention where there was a stronger association among younger and student samples. As students are predominantly younger, these findings undoubtedly overlap and suggest a greater consistency between favourability of opinions about donating and associated plans to donate among younger adults although attitude-intention correlations among all age (or sample type) cohorts were fairly strong. For participant gender, there was a stronger association between PBC and behaviour among female than male participants, with the correlation for male participants fairly low (*r*+ = .204). The difference suggests greater accuracy of control perceptions leading to subsequent donation among female participants.

For the moderators characterised by methodological differences, it was expected that TPB associations would be stronger when TA(C)T was used in the measurement of constructs in studies, and with higher attrition rates and shorter follow-up periods for studies including a measure of prospective behaviour. Although the TA(C)T prediction was supported for the PBC-behaviour association, contrary to expectations, the relationship between attitude and intention was stronger for studies not adhering to TA(C)T principles in measurement rather than those that did. The latter finding is contrary to other TPB meta-analytic results [29] and contrary to Ajzen’s [31] proposition about the need for specificity of measurement in TPB studies. It is possible it is difficult especially for some donation behaviours to adhere to a clear outline of likely behaviour engagement in relation to Target and Time elements (e.g., unexpected requests/appeals for monetary assistance, one-off nature of organ donation registration). In addition, due to the feasibility issue of some donation types not having a readily available context associated with donation (e.g., a specific charity request, donation centre, or blood drive known ahead of time), Context was not required as part of the definitional adherence which may have affected categorisation for this moderator. Consistent with expectations, the intention-behaviour link was stronger when there was some rather than no attrition in samples between assessing the predictors of intention and prospective behaviour, and when the follow up period between measurement phases was shorter rather than longer (with the PBC-behaviour link also strengthened with shorter follow-up periods). These findings indicate studies with a shorter follow-up time period and those subject to greater attrition may inflate TPB relationships when trying to extrapolate findings from shorter-term studies over longer durations and descriptions of the intention-behaviour relationship as demonstrating consistency without a consideration of the impact of participant attrition.

In an exploratory manner, the type of donation was explored to assess any differences between the dominant form of donation examined thus far in TPB studies (blood) and other types of charitable giving (money, time, goods etc). Both the subjective norm-intention and moral norm-intention links were stronger for types of donation other than blood. One possible explanation is that blood donation is often a solitary behaviour and less subject to normative pressures and social influences than other forms of giving such as volunteering in groups. In addition, blood donors may have fewer opportunities, compared to online charitable donors, to ‘advertise’ their actions despite efforts to enhance donor status recognition via digital ‘badges’ and other initiatives [42,43]. Efforts to strengthen the link between people’s norms and intentions for blood donation may benefit from a focus on fostering a supportive normative climate for blood donation (such as encouraging group-based donations in work teams and among friendship groups). Further, as the attitude-intention link also was weaker for blood than some other donation types, focusing on a cost-benefit analysis in favour of positive outcomes may assist in efforts to facilitate people’s strengthened plans to participate in blood drives.

### Limitations

A number of limitations should be taken into consideration when interpreting the results of the present study. The definition of the target behaviour was intentionally broad to encapsulate a range of giving behaviours but may have resulted in comparing forms of giving that may be quite disparate, especially in their level of commitment and degree of planning required (e.g., monetary donation to a mental health association vs. volunteering with elderly people in their homes). Relatedly, the stage of a donor’s ‘career’ is more relevant to some of the donation behaviours (e.g., blood donation, volunteering time) than others but was not assessed as a moderator especially given the limited number of studies assessing latter points of a donor’s trajectory (such as retention). With few studies employing a longitudinal approach to their data collection methodology, it prohibited an analysis of donation over time among the same cohort of donors.

Some limitations common to meta-analytic studies in general included: the likelihood of unaccounted sources of error such as measurement reactivity and error despite correcting for sampling error in the summary effects [44,45]; differences between studies in operationalisation of the model constructs; testing the predictive utility of the model based on a correlation matrix not necessarily representative of the full sample included; a small number of independent samples in each subgroup (k < 10) for some of the subgroup analyses; the predominance of data from a limited range of countries; and the significant heterogeneity that remained in nearly all subgroup analyses undertaken preventing an explanation of the dispersion in effect sizes.

For the sub-sample analyses, there were more instances of studies with older donors and female participants, which, although likely representative of donor demographic profiles [4], highlights a gap to examine younger and male samples to understand the determinants of their donation behaviours. While length of follow-up behaviour showed some differences in the association between both intention and PBC on donation behaviour, there was a disparity in the number of studies including an assessment of prospective behaviour although, for some donation behaviour types, a measure of follow-up behaviour is not always feasible (e.g., organ donation). For the moderator variables of age, gender, length of follow-up, and attrition, it would further have been preferable to use the original continuous coding in the moderator analyses; however, assumption violations warranted a less nuanced binary categorisation.

## Conclusion

Overall, this systematic review and meta-analysis provided support for the theory of planned behaviour as a parsimonious model reflecting the psychological determinants of people’s charitable donations across a range of donation behaviours. This support is particularly pronounced for predicting people’s charitable intentions, rather than donation behaviour. Control perceptions relating to donating, as well as a sense of moral obligation, emerged as the stronger influences of intentions, with intentions the strongest predictor of people’s donation behaviours. Stronger associations were established for the predictors of donation behaviour when the follow-up length was shorter and the relationship between norms (subjective and moral) and people’s intentions was more pronounced for charitable intentions for other forms of donation such as organs and time than blood donation. These findings can serve to inform practical strategies for charitable and not-for-profit organisations undertaking vital work assisting others by appealing to the relevant cognitions associated with people’s plans to give and their subsequent helping actions.

For future research avenues, given the shift to online forms of donation including volunteering and monetary donations, a comparison between face-to-face donations compared to online mediums may be a useful distinction in review analyses. Further exploration is needed to elucidate why the norm-intention links are stronger for charitable donations other than blood donation to assess the impact of the various sources of normative influence (personal, social). In addition, given the issue raised by researchers about the reverse-causal relations between intention and the TPB base constructs [46], future examinations of the efficacy of the model may benefit from a consideration of reciprocal causal relations. Based on the spontaneity of some charitable donations (e.g., online appeals for money donation), examining the utility of models that incorporate a less deliberative pathway [47] may be useful. This comparison may be beneficial given some donation type outcomes can only be feasibly examined in relation to an openness or willingness to donate (e.g., organ donation and donation in hypothetical scenarios). Comparisons between those studies employing an objective measure of charitable giving as opposed to self-report could be undertaken in future analyses on the basis of a sufficient number of studies including a prospective measure of observable/verifiable donations. Also, synthesizing experimental evidence for the TPB has the potential to inform more effective practical strategies to encourage charitable giving that target causal drivers of the behaviour. More primary experimental studies are needed to enable such synthesis.

As identified by the risk of bias assessment, the reliance on self-report rather than objective behaviour data when behaviour was measured is an additional consideration, reflecting a limitation of the area more generally. Similar to Bednall and Bove’s [25] meta-analysis assessing TPB blood donation research and identified in the risk of bias assessment in the present research, few studies examined and/or reported demographics related to ethnic minorities [48,49], suggesting a need for a focus on greater diversity and/or broader reporting of background demographics among future participant samples. As identified also in the risk of bias assessment, specifying the time period in which the study was conducted was rarely mentioned in studies, an inclusion becoming increasingly important in times of pandemics/natural disasters to assist in interpretation of findings around people’s donation choices and restrictions.

Although moral norm was identified as a common additional predictor for the TPB in the context of donation behaviour, as stated earlier, there is a range of other relevant psychological predictors to giving that could be examined when there are sufficient studies to enable a meaningful analysis. These constructs include anticipated regret for non-donation [50,51], guilt [52], role identity [11,53], and distinct types of norms such as descriptive and injunctive norms [10,54,55]. A consideration of personal traits such as altruism and empathy may prove worthwhile [5,56] although not often examined in TPB studies [57]. A few studies have also considered established personality traits in the TPB for donation [58] but they are in the minority. Further, characteristics related to the charitable organisations themselves, such as trust of and commitment to charities [59] have been examined recently as determinants to people’s donation decisions within the context of the TPB [60] and these organisational-centred variables may be worth considering in the context of established decision-making models once a critical mass of extant studies is obtained.

## Supporting information

S1 Checklist PRISMA checklist. (DOCX)

S1 Table Included studies and study characteristics. (DOCX)

S2 Table Coding of moderators. (DOCX)

S3 Table Risk of bias assessment. (PDF)

S1 File S1-S7 Figs. Forest and Funnel plots.
(PDF)
