# Supplementary material for: Charitable donations and the theory of planned behaviour: A systematic review and meta-analysis
Source: PLoS One. 2023 May 19;18(5):e0286053. doi: 10.1371/journal.pone.0286053 (PMC10198540; doi:10.1371/journal.pone.0286053)
Supplement: S1 Table — (DOCX) [file pone.0286053.s002.docx]

S1 Table.

Included Studies (in Chronological Order) and Study Characteristics

| **Author, Year** | **Country** | **Source** | **Design** | **Sample** | **Data collection period** | **Follow-up period** | **Target behaviour** |
| --- | --- | --- | --- | --- | --- | --- | --- |
| Zuckerman & Reis, 1978 [1] | USA | Published journal article | Prospective predictive | 135 male and 116 female undergraduate university students; age not reported; nil attrition | Not reported | 2 weeks | Blood donation |
| Hughes, 1984 [2] | USA | Dissertation | Prospective  predictive | 88 (60.2% male) randomly selected from a pool of about 5000 people affiliated with the Mental Health Association of Metropolitan Baltimore; Mage = 50.7 years; nil attrition | 1983 | 8 weeks | Monetary donation to a mental health association |
| Fortini, 1987 [3] | USA | Published journal article | Prospective predictive | 135 male/female rural public-school students in grades 5–7; age not reported | Not reported | 1 week | Signing up to become a volunteer peer tutor for "handicapped" students |
| Charng, Piliavin & Callero, 1988 [4] | USA | Published journal article | Prospective predictive | 658 (49.8% male) previous blood donors; M_age_ = 33 years; nil attrition | Aug–Sep 1981 | 7 months | Blood donation |
| Chafey, 1989 [5] | USA | Dissertation | Cross-sectional | 7 male and 69 female undergraduate nursing students; age 19–22 years = 36, 23–26 years = 28, 27–30 years = 13, 31–34 years = 14, 35–38 years = 3, 39 – 42 years = 2 | Not reported | N/A | Volunteer for the hospice care of an AIDS victim |
| Boride, Conner & Manteufel, 1992 [6] | USA | Book chapter | Prospective predictive | 88 male and 113 female potential kidney donors; M_age_ = 40 years, range = 16–72 years; 23% attrition | Feb 1989–Mar 1990 | < 13 months | Donating kidney to family member, spouse or friend |
| Jun, 1993 [7] | USA, Poland, & Korea | Dissertation | Prospective predictive | Male/female community blood donors  US sample: 658 participants; 49.8% male; M_age_ = 32.73 years  Poland sample: 915 participants; 96.8% male; M_age_ = 36.9 years  Korean sample: 200 participants; 87.8% male; M_age_ = 30.58 years | 1981–1984 | 6 months | Blood donation |
| Giles & Cairns, 1995 [8]  Giles, 1992 [9] | Ireland | Published journal article  Dissertation | Prospective predictive | 33 male and 108 female undergraduate students; M_age_ = 22.4 years | Not reported | 2 weeks | Blood donation |
| Harrison, 1995  [10]  Harrison, 1988  [11] | USA | Published journal article  Dissertation | Prospective predictive | Sample 1: 51 male homeless shelter volunteers; M_age_ = 39 years  Sample 2: 53 male homeless shelter volunteers; M_age_ = 39 years (cross-sectional)  Sample 3: 53 male homeless shelter volunteers; M_age_ = 35 years | 1987–1988 | Immediately after shift & 30 days | Attended work at a homeless shelter |
| Warburton, & Terry, 2000 [12] | Australia | Published journal article | Prospective predictive | 145 male and 151 female older adults; age range = 65–74 years; 18.9% attrition | Not reported | 4–6 weeks | Volunteering with any group or organisation |
| Armitage & Conner, 2001 (Study 1) [13] | UK | Published journal article | Cross-sectional | 23 male and 106 female prospective students attending university open day; M_age_ = 18.49 years, range = 14–50 years | Not reported | N/A | Blood donation |
| Armitage & Conner, 2001 (Study 2) [13] | UK | Published journal article | Cross-sectional | 64 male and 107 female undergraduate students; M_age_ = 23 years, range = 14–63 years | Not reported | N/A | Blood donation |
| Amponsah-Afuwape, Myers, & Newman, 2002 [14] | UK | Published journal article | Cross-sectional | 77 male and 69 female higher education students; M_age_ = 24.7 years, range = 18–56 years | Not reported | N/A | Blood donation |
| Kidwell, & Jewell, 2003  (Study 1) [15] | USA | Published journal article | Cross-sectional | 139 male/female university students; M_age_ = 21.2 years, range = 19–27 years | Not reported | N/A | Blood donation |
| Giles, McClenahan, Cairns & Mallet, 2004 [16] | Ireland | Published journal article | Prospective predictive | 21 male and 79 female undergraduate students; age not reported; attrition = 48% | Not reported | 2 weeks | Blood donation |
| Greenslade & White, 2005 [17] | Australia | Published journal article | Prospective predictive | 24 male and 117 female existing volunteers for a multi-purpose welfare organisation; M_age_ = 52.23 years, SD = 18.4, range = 15–88 years; attrition = 42.5% | May–Jun 2001 | 4 weeks | Volunteering for ≥ 3 hours per week |
| Holdershaw, 2005 (Pilot study) [18]  Holdershaw, Gendall & Wright, 2003 [19] | New Zealand | Dissertation  Published journal article | Prospective predictive | 100 male/female university students and staff; aged not reported; attrition = 60% | 2001 | 1 week | Blood donation |
| Holdershaw, 2005 (Main study) [18]  Holdershaw, Gendall, & Wright, 2011  [20] | New Zealand | Dissertation  Published journal article | Prospective predictive | 1262 male/female university students and staff; M_age_ = 22 years, range = 17–59 years; attrition = 20% | April 2002–May 2003 | 1 week | Blood donation |
| Lemmens et al., 2005 [21] | Netherlands | Published journal article | Cross-sectional | 46 male and 238 female undergraduate students who had not donated blood before: M_age_ = 19.7 years, range = 17–44 years | Not reported | N/A | Blood donation |
| Park & Smith, 2007 [22] | USA | Published journal article | Cross-sectional | 96 male and 165 female undergraduate students; M_age_ = 20.52 years, SD = 2.46 | Not reported | N/A | Signing state organ-donor registry |
| Smith & McSweeney, 2007 [23] | Australia | Published journal article | Prospective predictive | 60 male and 167 community members; M_age_ = 44.19 years; range = 17–82 years; attrition = 70.5% | Jun–Sep 2005 | 4 weeks | Monetary donation to a charity/community organisation |
| Weber, Martin & Corrigan, 2007 [24] | USA | Published journal article | Prospective predictive | 370 undergraduate students (182 male, 185 female, 3 unknown gender); age not reported, nil attrition | Not reported | Immediate | Signing an organ donor card |
| Bae & Kang, 2008 [25]  Bae, 2008 [26] | Korea | Published journal article  Published journal article | Cross-sectional | 1107 male and 951 female participants accessed via online panels of Korean research company: M_age_ = 34.38 years, SD = 11.9 | June 2005 | N/A | Signing a cornea donor card |
| Browne & Desmond, 2008 [27] | Ireland | Published journal article | Cross-sectional | 60 male and 75 female undergraduate students; M_age_ = 20.6 years, SD = 3.76 | Not reported | N/A | Donate part of liver posthumously |
| France, France & Himawan, 2008 [28]  France, France & Himawan, 2007 [29]  France, France & Himawan, 2008 [30] | USA | Published journal article  Published journal article  Conference presentation | Cross-sectional | 136 male and 328 female undergraduate psychology students; M_age_ = 18.9–19.1 years (reported for two subsamples), range = 17–45 years | Sep 2005–Nov 2006 | N/A | Blood donation within the next 8 weeks |
| Grano, Lucidi, Zelli & Violani, 2008 [31] | Italy | Published journal article | Prospective predictive | 312 male and 253 female older adult community organisation volunteers, M_age_ men 66.1 years, SD = 5.28; M_age_ women = 66.67 years, SD = 5.79; attrition = 6.7% | Not reported | 3 months | Volunteering |
| Henning, 2008 [32]  Henning, Huffman & Elandt, 2009 [33] | USA | Dissertation  Conference abstract | Cross-sectional | Sample 1: 40 employees of an organisation that supports volunteerism; 47.5% male; M_age_ = 37.59 years, SD = 11.76, age range = 20–62 years  Sample 2: 70 employees of an organisation that supports volunteerism; 21.4% male; M_age_ = 33.64 years, SD = 8.05, range = 23–55 years | Not reported | N/A | Participating in company supported volunteer activities |
| McMahon, & Byrne, 2008 [34] | Ireland | Published journal article | Prospective predictive | 46 male and 126 female university students and staff, M_age_ = 29.88 years, SD = 11.92, nil attrition | Not reported | 2 weeks | Blood donation |
| Robinson, Masser, White, Hyde & Terry, 2008 [35] | Australia | Published journal article | Cross-sectional | 195 community members; 43 male, 147 female and 5 unknown gender; Median age range = 35–44 years, 65% of respondents aged 25–54 years | Dec 2005–May 2006 | N/A | Blood donation |
| Lemmens et al., 2009 (Study 1) [36] | Netherlands | Published journal article | Cross-sectional | 61 male and 185 female distance-learning university students; M_age_ = 37.1 years, range = 19–66 years | Not reported | N/A | Blood donation |
| Lemmens et al., 2009 (Study 2) [36] | Netherlands | Published journal article | Cross-sectional | 219 male and 459 female  young people who had no experience of higher education; M_age_ = 23.1 years, range = 18–30 years | Not reported | N/A | Blood donation |
| Hyde & White, 2009a [37] | Australia | Published journal article | Prospective predictive | 94 male and 265 female undergraduate students and community members; M_age_ = 28.99 years, SD = 13.69, range = 17–65 years; attrition = 63.8% | Not reported | Contacted after 4 weeks, given up to 3 months to respond | Registering with the national organ donor register |
| Hyde & White, 2009b [38]  Hyde, & White, 2006 [39] | Australia | Published journal article  Conference abstract | Cross-sectional | 303 university students; 68 male, 233 female and 2 unknown gender; M_age_ = 23.5 years, SD = 8.3, 17–56 years | Not reported | N/A | Registering or continuing to be registered as a posthumous organ/tissue donor |
| Masser, White, Hyde, Terry & Robinson, 2009 [40]  Masser, White, Robinson, Hyde & Terry, 2007 [41] | Australia | Published journal article  Report | Prospective predictive | 101 male and 159 female community members; age range = 35–64 years; attrition = 30.8% | Not reported | 3 months | Visiting a blood collection site with the intention of giving blood |
| Park, Smith & Yun, 2009 [42] | USA | Published journal article | Cross-sectional | 2896 current or former employees of manufacturing and service industry companies; 50.8% male; M_age_ = 47.40 years, SD = 14.13 | Not reported | N/A | Enrolling in an organ donor registry in the near future |
| Hyde & White, 2010 [43] | Australia | Published journal article | Prospective predictive | 339 undergraduate students and community members; 35% male; M_age_ = 25.23 years, SD = 12.03, range = 17–77 years; attrition = 47.8% | Not reported | 4 weeks | Signing a registration form for the national organ donor registry |
| Lu, 2010 [44] | China | Dissertation | Prospective predictive | 100 male and 146 female university students; M_age_ = 22.30 years, SD = 1.92, range = 16–32 years; attrition = 69.9% | Not reported | 2 months | Blood donation |
| Stevenson, 2010 [45] | Central America | Dissertation | Cross-sectional | 115 male and 200 female community members; M_age_ = 32.1 years, SD = 12, range = 18–89 years | May–Jul 2009 | N/A | Donating some type of good or service at least once in the next 3 months to an organisation for children affected by HIV/AIDS |
| Yun & Park, 2010 [46] | USA | Published journal article | Cross-sectional | US and Korean university students  US sample: 290 participants; 43% male; M_age_ = 20.26 years, SD = 1.9  Korean sample: 292 participants; 52% male; M_age_ = 21.76 years, SD = 2.64 | Not reported | N/A | Signing up to an organ donor registry |
| Kinnally & Brinkerhoff, 2011 [47]  Kinnally & Brinkerhoff, 2013 [48] | USA | Published journal article  Published journal article | Cross-sectional | 984 participants recruited via public broadcast stations membership and donor email lists; 42% male; M_age_ = 55.6 years, SD = 14.2, range = 19–91 years | 2011 | N/A | Monetary donation to a public broadcasting station |
| Lee, 2011 [49] | USA | Dissertation | Cross-sectional | 291 potential volunteer tourists (members of volunteer tourism organisation or newsletter recipients); 24% male; modal age range = 21–30 years (44%), followed by 31–40 years (19.1%), then 41 -50 years (11.5%) and 51–60 years (11.5%) | Not reported | N/A | Volunteer tourism |
| van der Linden, 2011 [50] | UK | Published journal article | Cross-sectional | 62 male and 81 female community members; M_age_ = 28 years, SD = 8.5, range = 19–57 years | Not reported | N/A | Monetary donation to charity or community service organisation |
| Wang et al., 2011 [51] | China | Published journal article | Cross-sectional | 198 community health volunteers; 31.8% male; M_age_ = 54.3 years, SD = 10.1, range = 25–76 years | Jun–Jul 2008 | N/A | Participating in volunteering activities |
| Weberling, 2011 [52] | USA | Dissertation | Cross-sectional | 146 male and 368 female undergraduate students; M_age_ = 20 years, SD = 2.25 | Feb–Mar 2011 | N/A | Participating in a community fundraising event (Relay for Life) |
| Clowes & Masser, 2012 [53] | Australia | Published journal article | Experimental | 33 male and 43 female university students; M_age_ = 20.05 years, SD = 4.83, range = 17–46 years | Not reported | N/A | Blood donation in the next 3 months |
| Knowles, Hyde & White, 2012 [54] | Australia | Published journal article | Cross-sectional | 40 male and 170 female university students; M_age_ = 19.1 years, SD = 1.5; range = 18–24 years | 2004–2005 | N/A | Monetary donation to charity |
| Masser, Bednall, White & Terry, 2012 [55] | Australia | Published journal article | Prospective predictive | 53 male and 201 female first time blood donors; age range = 16–65+ years, 62.5% of sample aged 18–44 years; attrition ≈ 1% | Not reported | 4 & 8 months | Blood donation |
| McGlade, McClenahan & Pierscionek, 2012 [56] | Ireland | Published journal article | Cross-sectional | 3 male and 89 female pre-registered nursing university students; M_age_ = 24 years, SD = 5.6, range = 18–46 years | Jan 2012 | N/A | Registering consent to donate eyes upon death |
| Veldhuizen, Atsma, van Dongen & de Kort, 2012 [57] | Netherlands | Published journal article | Cross-sectional | 5540 male and 6511 female existing blood donors; M_age_ male donors = 48.6 years, SD = 11,8; M_age_ female donors = 42.5 years, SD = 12.8 | Jan 2007–Jan 2008 | N/A | Blood donation |
| Conner, Godin, Sheeran & Germain, 2013 [58] | Canada | Published journal article | Prospective predictive | 1108 male/female blood donors; age not reported; nil attrition | April 2003 | 6 months | Blood donation |
| Godin & Germain, 2013 [59] | Canada | Published journal article | Prospective predictive | 143 male and 109 female first-time blood donors; M_age_ = 40.94 years, SD = 14.67, range 18–70 years; nil attrition | Feb–Jun 2012 | 6 months | Plasma donation |
| Hyde, Knowles & Simon, 2013 [60]  Knowles, 2005a [61]  Hyde, Knowles, Simon & White, 2013 [62]  Knowles, 2005b [63] | Australia | Published journal article  Conference abstract  Published journal article  Conference abstract | Cross-sectional | 235 undergraduate psychology students; 44 male, 190 female, 1 unknown gender; M_age_ = 22.09 years; SD = 7.13  258 undergraduate psychology students; 18.7% male; M_age_ = 21.86 years, SD = 6.9, range = 17–59 years | Not reported | N/A | Volunteering for a community service  Blood and organ donation |
| Hyde & White 2013a  (Control group) [64] | Australia | Published journal article | Experimental | 24 male and 37 female community members; M_age_ = 48.79 years, SD = 13.83, range 18–70 years | Not reported | 1 month | Signing a registration form for the national organ donor register |
| Hyde & White, 2013b [65] | Australia | Published journal article | Prospective predictive | 174 undergraduate students not previously registered as a bone marrow donor; 23.3% male; M_age_ = 19.49 years, SD = 2.23; attrition = 46.6% | Not reported | 6 months | Preparatory steps in relation to joining the bone marrow registry |
| Jiranek, Kals, Humm, Strubel & Wehner, 2013 [66] | Switzerland | Published journal article | Cross-sectional | 513 community members; 63.5% male; M_age_ = 40.93 years, SD = 15.45, range = 16–85 years | Not reported | N/A | Participating in one of the following volunteer activities:  1) nursing/care for non-relatives in need  2) campaign  work in a humanitarian organization  3) office work  in an organization that helps people in need 4) assistance for non-relatives in need 5)  counselling/competence building for non-relatives in need |
| MacGillivray, Lynd-Stevenson, 2013 [67] | Australia | Published journal article | Cross-sectional | 72 male and 72 female community members; M_age_ = 34.19 years, SD = 16.5, range = 18–76 years | Jul–Sep 2009 | N/A | Volunteering ≥12 hours at an NPO/charity organisation |
| Newton, Newton, Ewing, Burney & Hay, 2013 [68]  Newton, Ewing, Burney & Hay, 2012 [69] | Australia | Published journal article  Published journal article | Cross-sectional | 352 community members; 136 male, 194 female, 22 unknown gender; M_age_ = 19.34 years, SD = 1.68, range = 18–24 years | Sep 2009–Feb 2010 | N/A | Registering as a posthumous organ donor |
| O'Brien, Fan, Yi & Goldman, 2013 [70] | Canada | Conference abstract | Prospective predictive | 520 new whole blood donors deferred within the previous month for short term malaria risk travel; 59.51% male; M_age_ = 33 years, range = 18–69 years | 2008 | 2 years | Blood donation |
| Polonsky, Renzaho, Ferdous & McQuilten, 2013 [71]  Ferdous, Polonsky, McQuilten & Renzaho, 2014 [72] | Australia | Published journal article  Book chapter | Cross-sectional | 425 African migrants and refugees in Victoria and South Australia aged > 16 years; 56.2% male; M_age_ = 33 years | Not reported | N/A | Blood donation |
| Veldhuizen & van Dongen, 2013 [73] | Netherlands | Published journal article | Cross-sectional | 4861 new blood donors  Whole blood donor sub-sample: 692 male and 1552 female, M_age_ = 34.3 years, SD = 12.6  Plasma sub-sample: 73 male and 147 female; M_age_ = 36.4 years, SD = 12.9 | Jul 2008–Mar 2009 | N/A | Blood donation |
| Veludo-de-Oliveira, Pallister, Foxall, & Gordon 2013 [74]  Veludo-de-Oliveira, 2009 [75] | UK | Published journal article  Dissertation | Prospective predictive | 28 male and 209 female volunteers for a charity which supports vulnerable young people; M_age_ = 20.3 years, SD = 1.26, range = 18–25 years; attrition = 32% | Nov/Dec 2007–April 2008 | 4–6 months | Volunteering |
| Alhidari, 2014 [76] | Saudi Arabia | Dissertation | Prospective | 294 male and 138 female charitable organisation donors; modal age range = 26–35 years (45.3%), followed by 36–45 years (23.3%), then 18–25 years (18.7%), 46–55 years (9.7%) and 56+ years (2.2%); nil attrition | Aug–Oct 2010 | 4 weeks | Monetary donation to charitable organisations |
| Bang, Odio & Reio, 2014 [77] | USA | Published journal article | Cross-sectional | 80 male and 27 female volunteers with a men’s basketball organisation; M_age_ = 28 years, range = 18–86 years | 2009 | N/A | Volunteering to assist for basketball tournament next year |
| Evans & Ferguson, 2014 [78] | UK | Published journal article | Cross-sectional | 414 university students; 159 male, 254 female, 1 unknown gender; M_age_ = 20.06 years, SD = 2.3, range = 17–39 years | Not reported | N/A | Blood donation |
| France et al., 2014 [79] | USA | Published journal article | Cross-sectional | Sample 2: 433 experience blood donors; 45% males M_age_ = 46.0 years, SD = 15.3 | Not reported | N/A | Blood donation in next 8 weeks |
| Huckins-Barker, 2014 [80] | USA | Dissertation | Experimental (prospective) | 390 college students; 43% male; 83.9% aged between 18–20 years | Not reported | 30 days | Blood donation |
| Kim & Lee, 2014 [81] | USA | Published journal article | Cross-sectional | 30 male and 170 female undergraduate students, M_age_ = 21 years, range = 19–33 years | Not reported | N/A | Volunteering for NPOs joined via social networking sites |
| Lee, Won & Bang, 2014 [82] | USA | Published journal article | Cross-sectional | 124 male and 138 female volunteers for a sporting event; M_age_ = 46.82 years, SD = 17.31 | 2008/2009 | N/A | Return to a volunteer activity |
| Siegel, Navarro, Tan & Hyde, 2014  (Study 2) [83] | Online (global), authors  based in USA | Published journal article | Cross-sectional | 358 participants recruited via crowd sourcing website, mTurk; 61.20% male; M_age_ = 30.71 years, SD = 11.46 | Not reported | N/A | Registering as an organ donor |
| Bagot, Masser & White, 2015 [84]  Masser, Bagot, White & Bove, 2013 [85] | Australia | Published journal article  Conference abstract | Prospective predictive | Sample 1: 527 recent whole blood donors who were asked to consider plasmapheresis donation; 55% male; M_age_ = 40.7 years, SD = 12.7  Sample 2: 166 previous blood donors who had not donated for 3–12 months; 53% male; M_age_ = 40.1 years, SD = 12.9 | Feb–Aug 2012 | 4–6 months | Plasma donation |
| Brayley et al., 2015 [86] | Australia | Published journal article | Cross-sectional | 186 older adults who were retired, semi-retired or approaching retirement; 56.1% male; M_age_ = 63.7 years, range = 50–86 years | Not reported | N/A | Engaging in episodic volunteering - defined as project-based volunteering undertaken for a period of ≤ 6 months |
| Delaney & White, 2015 [87] | Australia | Published journal article | Prospective predictive | 221 university students who were not currently registered body donors; 23.1% male; M_age_ = 26.28 years, SD = 9.01, range = 18–57 years; 49% attrition | Not reported | 2 months | Registering with a body bequest program within the next 2 months |
| Faqah, Moiz, Shahid, Ibrahim & Raheem, 2015 [88] | Pakistan | Published journal article | Cross-sectional | 121 male and 270 female medical students who had never donated blood before: M_age_ = 21.96 years, SD = 1.95 | Not reported | N/A | Blood donation |
| Kashif & De Run, 2015 [89] | Pakistan | Published journal article | Cross-sectional | 223 community members; 55% male; M_age_ = 24 years, range = 19–27 years | Not reported | N/A | Monetary donation to charities or community service organisations |
| Pavlova & Silbereisen 2015a [90]  2015b [91] | Germany | Published journal article  (Two records published in *Journal of Community and Applied Social Psychology*, & *Voluntas*) | Cross-sectional | Sample 1: 695 community members; 51.9% male; M_age_ = 29.9 years, SD = 6.3, range = 20–40 years  Sample 2: 694 community members; 51.7% male; M_age_ = 29.8 years, SD = 6.4, range = 20–40 years | Oct 2010–Jan 2011 | N/A | Civic and political volunteering |
| Reuveni & Werner, 2015 [92] | Israel | Published journal article | Cross-sectional | 258 9th grade high school students; 53.91% male; age not reported | June 2006 | N/A | Volunteering with elderly persons in their homes |
| Charsetad, 2016 [93] | Iran | Published journal article | Cross-sectional | 242 undergraduate university students; 113 male, 129 female; age not reported | Unclear | N/A | Blood donation |
| Mackay, White & Obst, 2016 [94] | Australia | Published journal article | Prospective predictive | 303 university students; 72% female; M_age_ = 25 years, range = 17–70 years; 43.6% attrition | Jul–Sep 2014 | 4 weeks | Online micro volunteering for non-profits, defined as bite-size volunteering with no commitment to repeat and with minimum formality, involving short and specific actions that are quick to start and complete |
| O'Carroll, Shepherd, Hayes & Eamonn, 2016  (Time 1) [95] | Scotland | Published journal article | Experimental | TPB/AR subsample: 446 community members; 152 male, 290 female, 4 unknown gender; M_age_ = 45.62 years, range = 19–61 years; nil attrition | April 2012 | 6 months | Registering as an organ donor |
| Britt, Britt & Anderson, 2017 [96] | USA | Published journal article | Cross-sectional | 150 university students; 43.8% male; M_age_ = 22.1 years, SD = 1.52 | Unclear | N/A | Signing organ donor card |
| Chen, 2017 [97] | China | Published journal article | Prospective predictive | 281 community members; at follow-up 117 male, 91 female; age 18–30 years = 71, 31–40 years = 48, 41–50 years = 26, 51–60 years = 63, 26% attrition | Unclear | 6 months | Blood donation |
| Hu, Wang & Fu, 2017 [98] | China | Published journal article | Prospective predictive | Sample 2: 861 community members; 329 male, 532 female; age 18–25 years = 689, 25–35 years = 139, 35–55 years = 33; 14% attrition | Dec 2013– Oct 2014 | 6 months | Blood donation |
| Poplaski, 2017 (main study) [99] | USA | Dissertation | Cross-sectional | 250 Christians from every major U.S. denomination; 98 male, 152 female; age 18–29 years = 48, 30–49 years = 84, 50–64 years = 77, 65 years and over = 41 | Unclear | N/A | Donations of money, property, or time to a non-profit organisation |
| Reynolds-Tylus & Quick, 2017 [100] | USA | Published journal article | Cross-sectional | 307 community members who were not previously registered as organ donors; 59% female; M_age_ = 20.64 years, SD = 2.22, range = 18–24 years | Jan 2010 | N/A | Joining the organ donor registry |
| Veludo-de-Oliveira, Alhaidari, Yani-de-Soriano & Yousafzai, 2017 [101] | Saudi Arabia | Published journal article | Prospective predictive | 432 community members over 18 years of age; 294 male, 138 female; age 18–25 years = 81, 26–35 years = 196, 36–45 years = 101, 46–55 years = 42, 56–65 years = 7, over 65 years = 5; 49% attrition | Unclear | 4 weeks | Monetary donation to a charitable organization |
| White, Poulsen & Hyde, 2017  [102] | Australia | Published journal article | Prospective predictive | 203 undergraduate psychology students; 48 male, 155 female; M_age_ = 21.63 years, SD = 7.54, range 17–65 years; 49% attrition | Aug–Dec 2007 | 3 months | Donating money to a charity or non-profit organization; volunteering time to a charity; and donating blood |
| Fox, Himawan & France, 2018 (Study 2) [103] | USA | Published journal article | Cross-sectional | 241 undergraduate students over 18 years of age; gender and age not reported | Unclear | N/A | Blood donation |
| Gellermann, 2018 [104] | Germany | Dissertation | Cross-sectional | 3231 community members; 45% male; M_age_ = 50 years, SD = 15.2, range = 18–75 years | Fall 2013 | N/A | Civic and political engagement |
| Gilchrist, Masser, Horsley & Ditto, 2019 [105] | Canada | Published journal article | Cross-sectional | 347 blood donors and non-donors recruited through campus advertisements; 23.7% male; M_age_ = 25.9 years, SD = 11.1, range = 18–65 years | Unclear | N/A | Blood donation |
| Li & Wu, 2019 [106] | China | Published journal article | Cross-sectional | 284 Chinese nationals and volunteers recruited from a nongovernmental organisation; 82 male, 202 female; M_age_ = 20.91 years, SD = 1.95, range = 17–38 years | Unclear | N/A | Volunteering work |
| Lu, Cheng, Lin & Chen, 2019 [107] | Taiwan | Published journal article | Prospective predictive | 391 university students majoring in sports and physical education; at follow-up 212 male, 135 female; M_age_ = 19.8 years, SD = 1.69, range = 18–33 years; 11.3% attrition | Unclear | 4 weeks | Sports volunteering |
| Alsalem, Fry, & Thaichon, 2020 [108] | Saudi Arabia | Published journal article | Cross-sectional | 423 Saudi Arabian Twitter users; 204 male and 219 female; age range 18–65 years | Not reported | N/A | Organ donation (registering to become a donor, discussing organ registration with family, and signing a donor card) |
| Costa, Alves, & Paco, 2020 [109] | Not reported  (authors based in Portugal) | Published journal article | Cross-sectional | 200 university students; 88 male and 112 female; M_age_ = 22.16 years | March, 2018 | N/A | Blood donation |
| Masser, Hyde, & Ferguson, 2020 [110] | Australia | Published journal article | Cross-sectional | 507 Australian residents aged 18-77 years who were eligible to donate whole blood, plasma, platelets; 236 male, 266 female and 5 non-specified; M_age_ = 28.69 years, SD = 10.43 | May 8-June 8, 2020 | N/A | Blood donation |
| Meng, Chua, Ryu, & Han, 2020 [111] | South Korea | Published journal article | Cross-sectional | 375 South Korean volunteers who had completed a non-profit global volunteer tourism program recruited at an event; 208 male and 167 female; M_age_ = 23.85 years | Feb17, 2019 | N/A | Re-volunteering |
| Meng, Ryu, Chua, & Han, 2020 [112] | South Korea | Published journal article | Cross-sectional | 320 South Korean university students participating in a 10-month overseas volunteer program recruited at homecoming festival; 152 male and 168 female; M_age_ = 23.68 years | Not reported | N/A | Continued volunteering |
| Hossain Parash, Suki, Shimmi, Hossain & Murthy, 2020 [113] | Malaysia | Published journal article | Cross-sectional | 455 students from a public higher learning institution; 145 male, 310 female; age 18–20 years = 131, 21–23 years = 249, over 24 years = 75 | Unclear | N/A | Blood donation |
| Kassie, Azale & Nigusie, 2020 [114] | Ethiopia | Published journal article | Cross-sectional | 515 community members; 66.4% female; M_age_ = 32.25 years, SD = 9.32, range 18–65 years | Mar 2019 | N/A | Blood donation |
| Aji, Albari Muthohar,  Sumadi, Sigit, Muslichah, & Hidayat, 2021 [115] | Indonesia | Published journal article | Cross-sectional | 560 Indonesian Muslim respondents recruited via several social media platforms; 284 male and 276 female; participants born <1965:48, 1965-1976: 79, 1977-1995: 138, >1995: 295 | Not reported | N/A | Making an online infaq (donation) |
| Aschale, Fufa, Kekeba & Birhanu, 2021 [116] | Ethiopia | Published journal article | Cross-sectional | 595 higher education students; 265 male and 330 female; M_age_ = 20.46 years, SD = 2.32 | Mar-April 1, 2019 | N/A | Blood donation |
| Duh & Dabula, 2021 [117] | South Africa | Published journal article | Cross-sectional | 650 university students; 42.8% male and 57.2% female; participants aged 18-35: 95.4%, 36-38: 4.6%, 40: 0.4% | Not reported | N/A | Blood donation |
| Chetioui, Satt, Lebdaoui, Baijou, Dassouli & Katona, 2022 [118] | Morocco | Published journal article | Cross-sectional | 377 Moroccan respondents recruited online; 181 male and 196 female; participants aged 18-25: 26.53%, 26-34: 38.46%, 35-44: 21.22% | June-Sept, 2020 | N/A | Monetary donation (Sadaqah donation) |
| Fernandes, Alessandri, Abbad, & Grano, 2022  [119] | Italy | Published journal article | Cross-sectional | Primiparous expectant mothers: 265 women; M_age_ = 31.22 years  Multiparous expectant mothers: 100 women; M_age_ = 33.09 years | Not reported | N/A | Blood cord donation |
| Li, Mao, & Liu, 2022  [120] | China | Published journal article | Cross-sectional | 721 Chinese Internet users 18 years and older; 272 male and 449 female; participants aged 18-25: 336 (46.6%); 26-35: 181 (25.1%); 36-45: 142 (19.7%); 46-55: 62 (8.6%) | Sept, 2019 | N/A | Monetary donation |

References

1. Zuckerman M, Reis HT. Comparison of three models for predicting altruistic behavior. J Pers Soc Psychol. 1978;36(5):498. doi: 10.1037/0022-3514.36.5.498

2. Hughes R. The interrelationship of attitude and past experience in the prediction of volunteer and donating behaviour. [Ph.D.]: Catholic University of America; 1984. Available from: <http://search.proquest.com/docview/303287956/>

3. Fortini M-E. Attitudes and behavior toward students with handicaps by their nonhandicapped peers. Am J Ment Defic. 1987.

4. Charng H-W, Piliavin J, Callero P. Role identity and reasoned action in the prediction of repeated behavior. Soc Psychol Q. 1988;51(4):303-17. doi: 10.2307/2786758.

5. Chafey KH. An exploration of the linkages among selected components of moral behavior. [PhD. Thesis]: University of Minnesota; 1989. Available from: <https://search.proquest.com/docview/303803533/>

6. Borgida E, Conner C, Manteufel L. Understanding Living Kidney Donation - a Behavioral Decision-Making Perspective. In: Spacapan S, Oskamp S, editors. Helping and being helped: naturalistic studies: Thousand Oaks, CA: : Sage Publications Inc; 1992.

7. Jun S. Cultural differences in altruistic action: comparing Korean, American, and Polish blood donors. [Ph.D.]: The University of Wisconsin-Madison; 1993. Available from: <https://www.proquest.com/docview/304087240?pq-origsite=gscholar&fromopenview=true>

8. Giles M, Cairns E. Blood donation and Ajzen's theory of planned behaviour: an examination of perceived behavioural control. Br J Soc Psychol. 1995;34(2):173-88.

9. Giles M. Attitude behaviour relations: an examination of the Ajzen and Fishbein approach. [PhD. Thesis]: New University of Ulster; 1992. Available from: <https://www.proquest.com/pqdtglobal/docview/301514214/F93427295042435CPQ/1?accountid=12372>

10. Harrison DA. Volunteer motivation and attendance decisions: Competitive theory testing in multiple samples from a homeless shelter. J Appl Psychol. 1995;80(3):371. doi: 10.1037//0021-9010.80.3.371.

11. Harrison DA. Attendance decisions: implications for absence from work. [Ph.D.]: University of Illinois; 1988. Available from: <http://search.proquest.com/docview/303668413/>

12. Warburton J, Terry D. Volunteer decision making by older people: a test of a revised theory of planned behavior. Basic Appl Soc Psychol. 2000;22(3):245-57. doi: 10.1207/15324830051036135.

13. Armitage C, Conner M. Social cognitive determinants of blood donation. J Appl Soc Psychol. 2001;31(7):1431-57. doi: DOI 10.1111/j.1559-1816.2001.tb02681.x. PubMed PMID: WOS:000170894100006.

14. Amponsah-Afuwape S, Myers L, Newman S. Cognitive predictors of ethnic minorities' blood donation intention. Psychol Health Med. 2002;7(3):357-61. doi: 10.1080/13548500220139359.

15. Kidwell B, Jewell R. The moderated influence of internal control: an examination across health‐related behaviours. J Consum Psychol. 2003;13(4):377-86. doi: 10.1207/S15327663JCP1304_05.

16. Giles M, McClenahan C, Cairns E, Mallet J. An application of the Theory of Planned Behaviour to blood donation: the importance of self-efficacy. Health Educ Res. 2004;19(4):380-91. Epub 2004/05/25. doi: 10.1093/her/cyg063. PubMed PMID: 15155590.

17. Greenslade JH, White KM. The prediction of above-average participation in volunteerism: a test of the theory of planned behavior and the volunteers functions inventory in older Australian adults. J Soc Psychol. 2005;145(2):155-72. doi: 10.3200/SOCP.145.2.155-172.

18. Holdershaw J. Comparison of two approaches to predicting blood donation behaviour. [Ph.D.]: Massey University; 2005. Available from: <https://mro.massey.ac.nz/bitstream/handle/10179/1726/02_whole.pdf>

19. Holdershaw J, Gendall P, Wright M. Predicting willingness to donate blood. Australas Market J. 2003;11(1):87-96. doi: 10.1016/S1441-3582(03)70120-7.

20. Holdershaw J, Gendall P, Wright M. Predicting blood donation behaviour: further application of the theory of planned behaviour. J Soc Mark. 2011;1(2):120-32. doi: 10.1108/20426761111141878.

21. Lemmens KPH, Abraham C, Hoekstra T, Ruiter RAC, De Kort WLAM, Brug J, et al. Why don't young people volunteer to give blood? an investigation of the correlates of donation intentions among young nondonors. Transfusion 2005;45(6):945-55. doi: 10.1111/j.1537-2995.2005.04379.x.

22. Park HS, Smith SW. Distinctiveness and influence of subjective norms, personal descriptive and injunctive norms, and societal descriptive and injunctive norms on behavioral intent: a case of two behaviors critical to organ donation. Hum Comm Res. 2007;33(2):194-218. doi: 10.1111/j.1468-2958.2007.00296.x.

23. Smith JR, McSweeney A. Charitable giving: the effectiveness of a revised theory of planned behaviour model in predicting donating intentions and behaviour. J Community Appl Soc Psychol. 2007;17(5):363-86. doi: 10.1002/casp.906.

24. Weber K, Martin MM, Corrigan M, Members of COMM 160 Real donors, real consent: testing the theory of reasoned action on organ donor consent. J Appl Soc Psychol. 2007;37(10):2435-50.

25. Bae H-S, Kang S. The Influence of viewing an entertainment–education program on cornea donation intention: a test of the theory of planned behavior. Health Commun. 2008;23(1):87-95. doi: 10.1080/10410230701808038.

26. Bae HS. Entertainment-education and recruitment of cornea donors: the role of emotion and issue involvement. J Health Commun. 2008;13(1):20-36. Epub 2008/03/01. doi: 10.1080/10810730701806953. PubMed PMID: 18307134.

27. Browne C, Desmond DM. Intention to consent to living organ donation: an exploratory study. Psychol Health Med. 2008;13(5):605-9. doi: 10.1080/13548500701842958.

28. France JL, France CR, Himawan LK. Re-donation intentions among experienced blood donors: does gender make a difference? Transfus Apher Sci. 2008;38(2):159-66. doi: 10.1016/j.transci.2008.01.001.

29. France JL, France CR, Himawan LK. A path analysis of intention to redonate among experienced blood donors: an extension of the theory of planned behavior. Transfusion. 2007;47(6):1006-13. doi: 10.1111/j.1537-2995.2007.01236.x.

30. France JL, France CR, Himawan LK. Comparing a predictive model of blood donation intention among donors and non-donors. Annals of Behavioral Medicine, 2008.

31. Grano C, Lucidi F, Zelli A, Violani C. Motives and determinants of volunteering in older adults: an integrated model. Int J Aging Hum Dev. 2008;67(4):305-26. doi: 10.2190/AG.67.4.b.

32. Henning JB. Antecedents of corporate volunteerism. [Ph.D.]: Texas A & M University; 2008. Available from: <http://search.proquest.com/docview/304179462/>

33. Henning JB, Huffman, A. H., & Elandt, A. M. The theory of planned behavior and corporate volunteerism. Paper presented at the 24th Annual Conference Program of the APA Division 14 Society for Industrial and Organizational Psychology; New Orleans, USA. 2009.

34. McMahon R, Byrne M. Predicting donation among an Irish sample of donors and nondonors: extending the theory of planned behavior. Transfusion. 2008;48(2):321-31. doi: 10.1111/j.1537-2995.2007.01526.x.

35. Robinson NG, Masser BM, White KM, Hyde MK, Terry DJ. Predicting intentions to donate blood among nondonors in Australia: an extended theory of planned behavior. Transfusion 2008;48(12):2559-67. doi: 10.1111/j.1537-2995.2008.01904.x.

36. Lemmens KPH, Abraham C, Ruiter RAC, Veldhuizen IJT, Dehing CJG, Bos AER, et al. Modelling antecedents of blood donation motivation among non-donors of varying age and education. Br J Psychol. 2009;100(1):71-90. doi: 10.1348/000712608X310237.

37. Hyde MK, White KM. Disclosing donation decisions: the role of organ donor prototypes in an extended theory of planned behaviour. Health Educ Res. 2009a;24(6):1080-92. doi: 10.1093/her/cyp028.

38. Hyde MK, White KM. To Be a Donor or Not to Be? applying an extended theory of planned behavior to predict posthumous organ donation intentions. J Appl Soc Psychol. 2009b;39(4):880-900. doi: 10.1111/j.1559-1816.2009.00464.x.

39. Hyde MK, & White, K. M. . Predicting posthumous organ donation intentions: applying an extended theory of planned behaviour. Aust J Psychol. 2006;58(S32).

40. Masser BM, White KM, Hyde MK, Terry D, Robinson N. Predicting blood donation intentions and behavior among Australian blood donors: testing an extended theory of planned behavior model. Transfusion 2009;49(2):320-9. doi: 10.1111/j.1537-2995.2008.01981.x.

41. Masser B, White K, Robinson N, Hyde M, Terry D. Recruiting and retaining Australian blood donors: A social psychological analysis of the role of attitudes, affect, norms, control factors and identity. Study 1. Preliminary Report, July 2007. Australian Red Cross Blood Service, 2007.

42. Park HS, Smith SW, Yun D. Ethnic differences in intention to enroll in a state organ donor registry and intention to talk with family about organ donation. Health Commun. 2009;24(7):647-59. doi: 10.1080/10410230903242259.

43. Hyde MK, White KM. Are organ donation communication decisions reasoned or reactive? a test of the utility of an augmented theory of planned behaviour with the prototype/willingness model. Br J Health Psychol. 2010;15(2):435-52. doi: 10.1348/135910709x468232

44. Lu J. Predicting blood donations among college students as a strategy to design voluntary blood donation campaigns in China. [Ph.D.]: Florida State University.; 2010. Available from: <http://search.proquest.com/docview/877923652/>

45. Stevenson LD. The culture of giving in the context of HIV/AIDS in the global south: a ase study from Belize. [Ph.D.]: Indiana University; 2010. Available from: <http://search.proquest.com/docview/726030745/>

46. Yun D, Park HS. Culture and the theory of planned behaviour: organ donation intentions in Americans and Koreans. J Pacific Rim Psychol. 2010;4(2):130-7. doi: 10.1375/prp.4.2.130.

47. Kinnally W, Brinkerhoff B. Modelling the members’ intentions to give: a case study using the TPB in the context of public broadcasting. J Creat Commun. 2011;6(3):297-313. doi: 10.1177/0973258613491665.

48. Kinnally W, Brinkerhoff B. Improving the drive: a case study for modeling public radio member donations using the theory of planned behavior. J Radio Audio Media. 2013;20(1):2-16. doi: 10.1080/19376529.2013.777733.

49. Lee SJ. Volunteer tourists' intended participation: using the revised Theory of Planned Behavior. [Ph.D.]: Virginia Polytechnic Institute and State University; 2011. Available from: <http://search.proquest.com/docview/1033590805/>

50. van der Linden S. Charitable Intent: A moral or social construct? a revised theory of planned behavior model. Curr Psychol. 2011;30(4):355-74. doi: 10.1007/s12144-011-9122-1.

51. Wang JW, Wei CN, Harada K, Minamoto K, Ueda K, Cui HW, et al. Applying the social cognitive perspective to volunteer intention in China: the mediating roles of self-efficacy and motivation. Health Promot Int. 2011;26(2):177-87. doi: 10.1093/heapro/daq056. PubMed PMID: 20819830.

52. Weberling B. From awareness to advocacy: understanding communication about cancer and nonprofit support. [Ph.D.]: University of North Carolina at Chapel Hill.; 2011. Available from: <http://search.proquest.com/docview/901120461/>

53. Clowes R, Masser BM. Right here, right now: the impact of the blood donation context on anxiety, attitudes, subjective norms, self‐efficacy, and intention to donate blood. Transfusion. 2012;52(7):1560-5. doi: 10.1111/j.1537-2995.2011.03486.x.

54. Knowles S, Hyde MK, White KM. Predictors of young people's charitable intentions to donate money: an extended theory of planned behavior perspective. J Appl Soc Psychol. 2012;42(9):2096-110. doi: 10.1111/j.1559-1816.2012.00932.x.

55. Masser BM, Bednall TC, White KM, Terry D. Predicting the retention of first‐time donors using an extended theory of planned behaviour. Transfusion. 2012;52(6):1303-10. doi: 10.1111/j.1537-2995.2011.03479.x.

56. McGlade D, McClenahan C, Pierscionek B. Attitudes underlying corneal donation in a group of trainee allied health professionals. Plos One. 2012;7(12):e53538. doi: 10.1371/journal.pone.0053538.

57. Veldhuizen I, Atsma F, van Dongen A, de Kort W. Adverse reactions, psychological factors, and their effect on donor retention in men and women. Transfusion. 2012;52(9):1871-9. doi: 10.1111/j.1537-2995.2011.03551.x.

58. Conner M, Godin G, Sheeran P, Germain M. Some feelings are more important: Cognitive attitudes, affective attitudes, anticipated affect, and blood donation. Health Psychol. 2013;32(3):264. doi: 10.1037/a0028500.

59. Godin G, Germain M. Predicting first lifetime plasma donation among whole blood donors. Transfusion. 2013;53:157S-61S. doi: 10.1111/trf.12475

60. Hyde MK, Knowles SR. What predicts Australian university students' intentions to volunteer their time for community service? Aust J Psychol. 2013;65(3):135-45. doi: 10.1111/ajpy.12014.

61. Knowles S. Using an extended theory of planned behaviour to predict intention towards blood donation. Aust J Psychol. 2005a;(57):91. doi: 10.1080/00049530600940006.

62. Hyde MK, Knowles SR, White KM. Donating blood and organs: using an extended theory of planned behavior perspective to identify similarities and differences in individual motivations to donate. Health Educ Res. 2013;28(6):1092-104. doi: 10.1093/her/cyt078.

63. Knowles S. Using an extended theory of planned behaviour to predict intention towards charitable donation. Aust J Psychol. 2005b;58:S34.

64. Hyde MK, White KM. A test of three interventions to promote people's communication of their consent for organ donation. Psychol Health. 2013a;28(4):399-417. doi: 10.1080/08870446.2012.731060.

65. Hyde MK, White KM. Testing an extended theory of planned behavior to predict young people's intentions to join a bone marrow donor registry. J Appl Soc Psychol. 2013b;43(12):2462-7. doi: 10.1111/jasp.12195.

66. Jiranek P, Kals E, Humm JS, Strubel IT, Wehner T. Volunteering as a means to an equal end? the impact of a social justice function on intention to volunteer. J Soc Psychol. 2013;153(5):520-41. doi: 10.1080/00224545.2013.768594.

67. MacGillivray GS, Lynd-Stevenson RM. The revised theory of planned behavior and volunteer behavior in Australia. Comm Dev (Columb). 2013;44(1):23-37. doi: 10.1080/15575330.2012.675578.

68. Newton JD, Newton FJ, Ewing MT, Burney S, Hay M. Conceptual overlap between moral norms and anticipated regret in the prediction of intention: implications for theory of planned behaviour research. Psychol Health. 2013;28(5):495-513. doi: 10.1080/08870446.2012.745936.

69. Newton JD, Ewing MT, Burney S, Hay M. Resolving the theory of planned behaviour's 'expectancy-value muddle' using dimensional salience. Psychol Health. 2012;27(5):588-602. doi: 10.1080/08870446.2011.611244.

70. O'Brien S, Fan, W., Yi, Q. L., & Goldman, M. R. . Donor motivational factors predicting donor return after malaria risk travel deferral: an application of path analysis. Paper presented at the 23rd Regional Congress of the International Society of Blood Transfusion; Amsterdam, The Netherlands.2013.

71. Polonsky MJ, Renzaho AMN, Ferdous AS, McQuilten Z. African culturally and linguistically diverse communities' blood donation intentions in Australia: integrating knowledge into the theory of planned behavior. Transfusion. 2013;53(7):1475. doi: 10.1111/j.1537-2995.2012.03915.x.

72. Ferdous AS, Polonsky MJ, McQuilten ZK, Renzaho A. Case study: using the theory of planned behaviour to assess blood donation intentions amongst African migrants in Australia. In: Brennan L, Binney, W., & Parker, L. , editor. Social marketing behaviour change: models, theory and applications 2014. p. 29-36.

73. Veldhuizen I, van Dongen A. Motivational differences between whole blood and plasma donors already exist before their first donation experience. Transfusion. 2013;53(8):1678. doi: 10.1111/trf.12056.

74. Veludo-de-Oliveira T, Pallister JG, Foxall GR. Accounting for sustained volunteering by young people: an Expanded TPB. Voluntas 2013;24(4):1180-98. doi: 10.1007/s11266-012-9317-6.

75. Veludo-de-Oliveira TM. Social marketing, volunteering, and the theory of planned behaviour: what is behind volunteering behaviour? [Ph.D.]: Cardiff University; 2009. Available from: <https://ethos.bl.uk/OrderDetails.do?uin=uk.bl.ethos.584710>

76. Alhidari I. Investigating individuals’ monetary donation behaviour in Saudi Arabia. PhD. Thesis: Cardiff University; 2014. Available from: <https://orca-mwe.cf.ac.uk/62661/1/2014%20Alhidari%20Ibrahim.pdf>

77. Bang H, Odio MA, Reio T. The moderating role of brand reputation and moral obligation: an application of the theory of planned behavior. J Manag Dev. 2014;33(4):282-98. doi: 10.1108/JMD-12-2010-0102.

78. Evans R, Ferguson E. Defining and measuring blood donor altruism: a theoretical approach from biology, economics and psychology. Vox Sang. 2014;106(2):118-26. doi: 10.1111/vox.12080.

79. France JL, Kowalsky JM, France CR, McGlone ST, Himawan LK, Kessler DA, et al. Development of common metrics for donation attitude, subjective norm, perceived behavioral control, and intention for the blood donation context. Transfusion. 2014;54(3pt2):839-47. doi: 10.1111/trf.12471.

80. Huckins-Barker JL. Using multimedia blood donation education materials to enhance individual readiness to donate blood and increase donation behaviors. [Ph.D.]: Ohio University; 2014. Available from: <https://etd.ohiolink.edu/apexprod/rws_etd/send_file/send?accession=ohiou1389353647&disposition=inline>

81. Kim Y, Lee W-N. Networking for philanthropy: increasing volunteer behavior via social networking sites. Cyberpsychol Behav Soc Netw. 2014;17(3):16-165. doi: 10.1089/cyber.2012.0415.

82. Lee Y-J, Won D, Bang H. Why do event volunteers return? theory of planned behavior. Int Rev Pub Nonprofit Mark. 2014;11(3):229-41. doi: 10.1007/s12208-014-0117-0.

83. Siegel JT, Navarro MA, Tan CN, Hyde MK. Attitude–behavior consistency, the principle of compatibility, and organ donation: a classic innovation. Health Psychol. 2014;33(9):1084-91. doi: 10.1037/hea0000062.

84. Bagot KL, Masser BM, White KM. Using an extended theory of planned behavior to predict a change in the type of blood product donated. Ann Behav Med. 2015;49(4):510-21. doi: 10.1007/s12160-014-9677-9.

85. Masser BM, Bagot KL, White M, Bove L. How can 'blood' collection agencies facilitate first time plasmapaheresis donation? Vox Sang. 2013;105(s1):S87-8. doi: 10.111/vox.12048.

86. Brayley N, Obst P, White KM, Lewis I, Warburton J, Spencer N. Examining the predictive value of combining the theory of planned behaviour and the volunteer functions inventory. Aust J Psychol. 2015;67(3):149-56. doi: 10.1111/ajpy.12078.

87. Delaney MF, White KM. Predicting people's intention to donate their body to medical science and research. J Soc Psychol. 2015;155(3):221-37. doi: 10.1080/00224545.2014.998962.

88. Faqah A, Moiz B, Shahid F, Ibrahim M, Raheem A. Assessment of blood donation intention among medical students in Pakistan: an application of theory of planned behavior. Transfus Apher Sci. 2015;53(3):353-9. doi: 10.1016/j.transci.2015.07.003.

89. Kashif M, De Run EC. Money donations intentions among Muslim donors: an extended theory of planned behavior model. Int J Nonprofit Volunt Sect Mark. 2015;20(1):84-96. doi: 10.1002/nvsm.1519.

90. Pavlova MK, Silbereisen RK. Factual versus potential civic participation in a Post-Communist region: a typological approach. Voluntas. 2015a;26(3):941-61. doi: 10.1007/s11266-014-9483-9.

91. Pavlova MK, Silbereisen RK. Supportive social contexts and intentions for civic and political participation: an application of the theory of planned behaviour. J Community Appl Soc Psychol. 2015b;25(5):432-46. doi: 10.1002/casp.2223.

92. Reuveni Y, Werner P. Factors associated with teenagers' willingness to volunteer with elderly persons: application of the theory of planned behavior (TPB). Educ Gerontol. 2015;41(9):623-34. doi: 10.1080/03601277.2015.1029768.

93. Charsetad P. Role of religious beliefs in blood donation behavior among the youngster in Iran:a theory of planned behavior perspective. J Islamic Mark. 2016;7(3):250-63. doi: 10.1108/JIMA-05-2014-0037.

94. Mackay SA, White KM, Obst PL. Sign and share: what influences our participation in online microvolunteering. Cyberpsychol Behav Soc Netw. 2016;19(4):257-63. doi: 10.1089/cyber.2015.0282.

95. O'Carroll RE, Shepherd L, Hayes PC, Ferguson E. Anticipated regret and organ donor registration: a randomized controlled trial. Health Psychol. 2016;35(11):1169. doi: 10.1037/hea0000363.

96. Britt RK, Britt BC, Anderson J. Theoretical implications addressing rural college students’ organ donation behaviors. J Health Psychol. 2017;22(5):650-60. doi: 10.1177/1359105315611953.

97. Chen L. Applying the extended theory of planned behaviour to predict Chinese people's non‐remunerated blood donation intention and behaviour: the roles of perceived risk and trust in blood collection agencies. Asian J Soc Psychol. 2017;20(3-4):221-31. doi: 10.1111/ajsp.12190.

98. Hu H, Wang T, Fu Q. Psychological factors related to donation behaviour among Chinese adults: results from a longitudinal investigation. Transfus Med 2017;27(5):335-41. doi: 10.1111/tme.12422.

99. Poplaski SC. Charitable behavior: Christian beliefs that explain donor intentions. [Ph.D.]: Kansas State University; 2017. Available from: <https://krex.k-state.edu/dspace/handle/2097/35283>

100. Reynolds-Tylus T, Quick BL. Examining differences in predictors of African American, Caucasian, and Latino young adults' intentions to register as an organ donor. J Broadcast Electron Media. 2017;61(2):368-92. doi: 10.1080/08838151.2017.1309413.

101. Veludo-de-Oliveira TM, Alhaidari IS, Yani-de-Soriano M, Yousafzai SY. Comparing the explanatory and predictive power of intention-based theories of personal monetary donation to charitable organizations. Voluntas 2017;28(2):571-93. doi: 10.1007/s11266-016-9690-7.

102. White KM, Poulsen B, Hyde MK. Identity and personality influences on donating money, time, and blood. Nonprofit Volunt Sect Q. 2017;46(2):372-94. doi: 10.1177/0899764016654280.

103. Fox KR, Himawan LK, France CR. The blood donation ambivalence survey: measuring conflicting attitudes about giving blood. Transfus Med. 2018;28(3):193-9. doi: 10.1111/tme.12426.

104. Gellermann J. Psychosocial predictors of intentions for civic and political engagement among currently engaged and unengaged individuals. PhD. Thesis. 2018

105. Gilchrist PT, Masser BM, Horsley K, Ditto B. Predicting blood donation intention: the importance of fear. Transfusion. 2019;59(12):3666-73. doi: 10.1111/trf.15554.

106. Li C, Wu Y. Understanding voluntary intentions within the theories of self-determination and planned behavior. J Nonprofit Pub Sec Mark. 2019;31(4):378-89. doi: 10.1080/10495142.2018.1526745.

107. Lu WC, Cheng C-F, Lin S-H, Chen M-Y. Sport volunteering and well-being among college students. Curr Psychol. 2019;38(5):1215-24. doi: 10.1007/s12144-017-9663-z.

108. Alsalem A, Fry ML, Thaichon P. To donate or to waste it: Understanding posthumous organ donation attitude. Australasian Marketing Journal. 2020;28(3):87-97. doi: 10.1016/j.ausmj.2020.04.001. PubMed PMID: WOS:000572687500011.

109. Costa AR, Alves H, Paco A. Explanatory factors of blood-giving in young adults: An extended theory of planned behaviour model. International Journal of Nonprofit and Voluntary Sector Marketing. 2020;25(4). doi: 10.1002/nvsm.1674. PubMed PMID: WOS:000531399500001.

110. Masser BM, Hyde MK, Ferguson E. Exploring predictors of Australian community members' blood donation intentions and blood donation-related behavior during the COVID-19 pandemic. Transfusion. 2020;60(12):2907-17. Epub 2020/09/10. doi: 10.1111/trf.16067. PubMed PMID: 32905630.

111. Meng B, Chua BL, Ryu HB, Han H. Volunteer tourism (VT) traveler behavior: merging norm activation model and theory of planned behavior. Journal of Sustainable Tourism. 2020;28(12):1947-69. doi: 10.1080/09669582.2020.1778010.

112. Meng B, Ryu HB, Chua BL, Han H. Predictors of intention for continuing volunteer tourism activities among young tourists. Asia Pacific Journal of Tourism Research. 2020;25(3):261-73. doi: 10.1080/10941665.2019.1692046.

113. Hossain Parash M, Suki NM, Shimmi SC, Hossain ABMT, Murthy KD. Examining students’ intention to perform voluntary blood donation using a theory of planned behaviour: a structural equation modelling approach. Transfus Clin Bio. 2020;27(2):70-7. doi: 10.1016/j.tracli.2020.02.002.

114. Kassie A, Azale T, Nigusie A. Intention to donate blood and its predictors among adults of Gondar city: using theory of planned behavior. PloS One. 2020;15(3):e0228929-e. doi: 10.1371/journal.pone.0228929.

115. Aji HM, Albari A, Muthohar M, Sumadi S, Sigit M, Muslichah I, et al. Investigating the determinants of online infaq intention during the COVID-19 pandemic: an insight from Indonesia. Journal of Islamic Accounting and Business Research. 2021;12(1):1-20. doi: 10.1108/jiabr-05-2020-0136. PubMed PMID: WOS:000597962800001.

116. Aschale A, Fufa D, Kekeba T, Birhanu Z. Intention to voluntary blood donation among private higher education students, Jimma town, Oromia, Ethiopia: Application of the theory of planned behaviour. PLoS One. 2021;16(3):e0247040. Epub 2021/03/03. doi: 10.1371/journal.pone.0247040. PubMed PMID: 33651830; PubMed Central PMCID: PMCPMC7924737.

117. Duh HI, Dabula N. Millennials' socio-psychology and blood donation intention developed from social media communications: A survey of university students. Telematics and Informatics. 2021;58. doi: 10.1016/j.tele.2020.101534. PubMed PMID: WOS:000654053900013.

118. Chetioui Y, Satt H, Lebdaoui H, Baijou M, Dassouli S, Katona S. Antecedents of giving charitable donations (Sadaqah) during the COVID-19 pandemic: does Islamic religiosity matter? Journal of Islamic Marketing. 2022. doi: 10.1108/JIMA-09-2021-0296.

119. Fernandes M, Alessandri G, Abbad R, Grano C. Determinants of the intention to donate umbilical cord blood in pregnant women. Vox Sang. 2022;117(2):169-76. Epub 2021/07/16. doi: 10.1111/vox.13179. PubMed PMID: 34263455.

120. Li W, Mao Y, Liu C. Understanding the Intention to Donate Online in the Chinese Context: The Influence of Norms and Trust. Cyberpsychology. 2022;16(1). doi: 10.5817/CP2022-1-7.
