# Supplementary material for: Charitable donations and the theory of planned behaviour: A systematic review and meta-analysis
Source: PLoS One. 2023 May 19;18(5):e0286053. doi: 10.1371/journal.pone.0286053 (PMC10198540; doi:10.1371/journal.pone.0286053)
Supplement: S2 Table — (DOCX) [file pone.0286053.s003.docx]

S2 Table

Coding of Moderators

|  | Participant age | Participant gender | Type of sample | TA(C)T | Follow-up time period | Attrition | Type of behaviour |
| --- | --- | --- | --- | --- | --- | --- | --- |
| Codes/Categories  Authors | Mean age  (Young <25; Older ≥25)  Unclear | % female  (Majority male/female)  Unclear | Student  Non-student  Unclear | Yes  No | # days  (short <2 months; long ≥2 months)  Not applicable | % attrition  (No/any attrition)  Unclear  Not applicable | Blood  Organs  Time  Money  Mixed |
| Aji, Albari Muthohar, Sumadi, Sigit, Muslichah, & Hidayat, 2021 [115] | Unclear* | 49.3  (Majority male) | Non-student | No | N/A | N/A | Money |
| Alhidari, 2014 [76] | Unclear  (Older*) | 31.94  (Majority male) | Non-student | Yes | 28  (Short) | 0  (No) | Money |
| Alsalem, Fry, & Thaichon, 2020 [108] | Unclear | 52  (Majority female) | Non-student | No | N/A | N/A | Organs |
| Amponsah-Afuwape, Myers, & Newman, 2002 [14] | 24.70  (Young) | 47.26  (Majority male) | Student | Yes | N/A | N/A | Blood |
| Armitage & Conner, 2001: Study 1 [13] | 18.94  (Young) | 82.17  (Majority female) | Unclear* | No | N/A | N/A | Blood |
| Armitage & Conner, 2001: Study 2 [13] | 23  (Young) | 62.57  (Majority female) | Non-student* | No | N/A | N/A | Blood |
| Aschale, Fufa, Kekeba & Birhanu, 2021 [116] | 20.46  (Young) | 55.5  (Majority female) | Student | Yes | N/A | N/A | Blood |
| Bae & Kang, 2008 [25] | 34.38  (Older) | 46.21  (Majority male) | Non-student* | Yes | N/A | N/A | Organs |
| Bagot, Masser, & White, 2015: Sample 1 [84] | 40.7  (Older) | 45  (Majority male) | Non-student* | No | 150  (Long) | 0*  (No) | Blood |
| Bagot, Masser, & White, 2015: Sample 2 [84] | 40.1  (Older) | 47  (Majority male) | Non-student* | No | 150  (Long) | 0*  (No) | Blood |
| Bang, Odio, & Reio, 2014 [77] | 28  (Older) | 25.7  (Majority male) | Non-student | Yes | N/A | N/A | Time |
| Borgida, Conner, & Manteufel, 1992 [6] | 40  (Older) | 56.22  (Majority female) | Non-student | No | N/A | N/A | Organs |
| Brayley et al., 2015 [86] | 63.7  (Older) | 43.9  (Majority male) | Non-student | No | N/A | N/A | Time |
| Britt, Britt, & Anderson, 2017 [96] | 22.1  (Young) | 56.2  (Majority female) | Student | No | N/A | N/A | Organs |
| Browne & Desmond, 2008 [27] | 20.6  (Young) | 55.56*  (Majority female) | Student | No | N/A | N/A | Organs |
| Chafey, 1989 [5] | Unclear* | 92.71  (Majority female) | Student | No | N/A | N/A | Time |
| Charng, Piliavin, & Callero,  1988 [4] | 33  (Older) | 50.2  (Majority female) | Non-student | Yes | 210  (Long) | 0  (No) | Blood |
| Charsetad, 2016 [93] | Unclear | 53.31  (Majority female) | Student | Yes | N/A | N/A | Blood |
| Chen, 2017 [97] | Unclear  (Older) | 43.75  (Majority male) | Non-student | No | 180  (Long) | 26  (Any) | Blood |
| Chetioui, Satt, Lebdaoui, Baijou, Dassouli & Katona, 2022 [118] | Unclear | 52  (Majority female) | Non-student | No | N/A | N/A | Money |
| Clowes & Masser, 2012 [53] | 20.05  (Young) | 56.58  (Majority female) | Student | Yes | N/A | N/A | Blood |
| Conner, Godin, Sheeran, & Germain, 2013 [58] | Unclear | Unclear | Non-student | Yes | 180  (Long) | 0  (No) | Blood |
| Costa, Alves, & Paco, 2020 [109] | 22.16  (Young) | 56  (Majority female) | Student | No* | N/A | N/A | Blood |
| Delaney & White, 2015 [87] | 26.28  (Older) | 76.9  (Majority female) | Student | Yes | 60  (Long) | 49  (Any) | Organs |
| Duh & Dabula, 2021 [117] | Unclear | 57.2  (Majority female) | Student | No | N/A | N/A | Blood |
| Evans & Ferguson, 2014 [78] | 20.06  (Young) | 61.5  (Majority female) | Student | No* | N/A | N/A | Blood |
| Faqah, Moiz, Shahid, Ibrahim, & Raheem, 2015 [88] | 21.96  (Young) | 69.05  (Majority female) | Student | Yes | N/A | N/A | Blood |
| Fernandes, Alessandri, Abbad, & Grano, 2022: Sample 1 (Primiparous expectant mothers) [119] | 33.09  (Older) | 100  (Majority female) | Non-student | No | N/A | N/A | Blood |
| Fernandes, Alessandri, Abbad, & Grano, 2022: Sample 2 (Multiparous expectant mothers) | 31.22  (Older) | 100  (Majority female) | Non-student | No | N/A | N/A | Blood |
| Fortini, 1987 [3] | Unclear  (Young) | Unclear | Student | Yes | 7  (Short) | Unclear | Time |
| Fox, Himawan, & France, 2018: Study 2 [103] | Unclear | Unclear | Student | Yes | N/A | N/A | Blood |
| France, France, & Himawan, 2008 [28] | Unclear*  (Young) | 70.69*  (Majority female) | Student | No* | N/A | N/A | Blood |
| France et al., 2014 [79] | 46  (Older) | 55  (Majority female) | Non-student | Yes | N/A | N/A | Blood |
| Gellermann, 2018 [104] | 50  (Older) | 55  (Majority female) | Non-student | No | N/A | N/A | Time |
| Gilchrist, Masser, Horsley, & Ditto, 2019 [105] | 25.9  (Older) | 76.3  (Majority female) | Unclear* | Yes | N/A | N/A | Blood |
| Giles & Cairns, 1995 [8] | 22.3  (Young) | 76.6  (Majority female) | Student | Yes | 14  (Short) | Unclear | Blood |
| Giles, McClenahan, Cairns, & Mallet, 2004 [16] | Unclear | 79  (Majority female) | Student | Yes | 14  (Short) | 48  (Any) | Blood |
| Godin & Germain, 2013 [59] | 40.94  (Older) | 43.25  (Majority male) | Non-student | Yes | 180  (Long) | 0  (No) | Blood |
| Grano, Lucidi, Zelli, & Violani, 2008 [31] | 66.35*  (Older) | 44.78  (Majority male) | Non-student | Yes | 90  (Long) | 6.7  (Any) | Time |
| Greenslade & White, 2005 [17] | 52.23  (Older) | 82.98  (Majority female) | Non-student | Yes | 28  (Short) | 42.5  (Any) | Time |
| Harrison, 1995: Sample 1 [10] | 39  (Older) | 0  (Majority male) | Non-student | Yes | 15  (Short) | 3.9*  (Any) | Time |
| Harrison, 1995: Sample 2 [10] | 39  (Older) | 0  (Majority male) | Non-student | Yes | 15  (Short) | 5.7*  (Any) | Time |
| Harrison, 1995: Sample 3 [10] | 35  (Older) | 0  (Majority male) | Non-student | Yes | 15  (Short) | 7.5*  (Any) | Time |
| Henning, 2008 [32] | 35.08  (Older) | 69.09  (Majority female) | Non-student | Yes | N/A | N/A | Time |
| Holdershaw, 2005: pilot study [18] | Unclear | Unclear | Non-student | Yes | 7  (Short) | 60  (Any) | Blood |
| Holdershaw, 2005: main study [18] | 22  (Young) | Time 1: Unclear Time 2: 60.32  (Majority female) | Non-student | Yes | 7  (Short) | 20  (Any) | Blood |
| Hossain Parash, Suki, Shimmi, Hossain, & Murthy, 2020 [113] | Unclear | 68.13  (Majority female) | Student | No | N/A | N/A | Blood |
| Hu, Wang, & Fu, 2017: Sample 2 [98] | Unclear | 61.79  (Majority female) | Non-student | Yes | 180  (Long) | 14  (Any) | Blood |
| Huckins-Barker, 2014 [80] | Unclear  (Young) | 57  (Majority female) | Student | Yes | 30  (Short) | Unclear | Blood |
| Hughes, 1984, sample 1 [2] | Unclear  (Older) | Unclear  (Majority male) | Non-student | No | 56  (Long) | 0*  (No) | Money |
| Hughes, 1984, sample 2 [2] | Unclear  (Older) | Unclear  (Majority male) | Non-student | No | 56  (Long) | 0*  (No) | Money |
| Hughes, 1984, sample 3 [2] | Unclear  (Older) | Unclear  (Majority male) | Non-student | No | 56  (Long) | 0*  (No) | Money |
| Hughes, 1984, sample 4 [2] | Unclear  (Older) | Unclear  (Majority male) | Non-student | No | 56  (Long) | 0*  (No) | Money |
| Hyde & Knowles, 2013: volunteering [60] | 22.09  (Young) | 81.2  (Majority female) | Student | No | N/A | N/A | Money |
| Hyde, Knowles, & White, 2013: blood, organ donation [62] | 21.86  (Young) | 81.3  (Majority female) | Student | No | N/A | N/A | Mixed |
|  |  |  |  |  |  |  |  |
| Hyde & White, 2009a: registering behaviour [37] | Time 1: 28.99 Time 2: 28.44  (Older) | Time 1: 73.82 Time 2: 73.08  (Majority female) | Non-student | No | 28  (Short) | 63.8  (Any) | Organs |
| Hyde & White, 2009b: registering behaviour [38] | 23.5  (Young) | 77.41  (Majority female) | Student | No | N/A | N/A | Organs |
| Hyde & White, 2010: registering behaviour [43] | Time 1: 25.23  (Older)  Time 2: 24.66  (Young) | Time 1: 65 Time 2: 69  (Majority female) | Non-student | Yes | 28  (Short) | 47.8  (Any) | Organs |
| Hyde & White, 2013a: registering behaviour, control group [64] | 48.79  (Older) | 60.66  (Majority female) | Non-student | Yes* | 30  (Short) | 0  (No) | Organs |
| Hyde & White, 2013b [65] | Time 1: 19.49  Time 2: 19.70  (Young) | Time 1: 76.7 Time 2: 79.6*  (Majority female) | Student | Yes | 90  (Long) | 46.6  (Any) | Organs |
| Jiranek, Kals, Humm, Strubel, & Wehner, 2013 [66] | 40.93  (Older) | 36.5  (Majority male) | Non-student | No | N/A | N/A | Time |
| Jun, 1993: US sample [7] | 32.73  (Older) | 50.2  (Majority female) | Non-student | Yes | 180  (Long) | 0*  (No) | Blood |
| Jun, 1993: Polish sample, men [7] | 36.9  (Older) | 3.2  (Majority male) | Non-student | Yes | 180  (Long) | 0*  (No) | Blood |
| Jun, 1993: Korean sample, men [7] | 30.58  (Older) | 12.2  (Majority male) | Non-student | Yes | 180  (Long) | 0*  (No) | Blood |
| Kashif & De Run, 2015 [89] | 24  (Young) | 45  (Majority male) | Non-student | Yes | N/A | N/A | Money |
| Kassie, Azale, & Nigusie, 2020 [114] | 32.25*  (Older) | 66.4  (Majority female) | Non-student | Yes | N/A | N/A | Blood |
| Kidwell & Jewell, 2003: Study 1, blood donation behaviour [15] | 21.2  (Young) | Unclear | Student | Yes* | N/A | N/A | Blood |
| Kim & Lee, 2014 [81] | 21  (Young) | 58.62  (Majority female) | Student | No | N/A | N/A | Time |
| Kinnally & Brinkerhoff, 2011 [47] | 55.6  (Older) | 58  (Majority female) | Non-student | No* | N/A | N/A | Money |
| Knowles, Hyde, & White, 2012 [54] | 19.1  (Young) | 80.95  (Majority female) | Student | No | N/A | N/A | Money |
| Lee, 2011 [49] | Unclear  (Older*) | 76  (Majority female) | Non-student | Yes | N/A | N/A | Time |
| Lee, Won, & Bang, 2014 [82] | 46.82  (Older) | 52.67  (Majority female) | Non-student | Yes | N/A | N/A | Time |
| Lemmens et al., 2005 [21] | 19.77  (Young) | 83.8  (Majority female) | Student | No* | N/A | N/A | Blood |
| Lemmens et al., 2009: Study 1 [36] | 37.1  (Older) | 75.2  (Majority female) | Student | Yes | N/A | N/A | Blood |
| Lemmens et al., 2009: Study 2 [36] | 23.1  (Young) | 67.7  (Majority female) | Non-student | Yes | N/A | N/A | Blood |
| Li, Mao, & Liu, 2022 [120] | Unclear  (Older) | 62.3  (Majority female) | Non-student | Yes | N/A | N/A | Money |
| Li & Wu, 2019 [106] | 20.91  (Young) | 71.13  (Majority female) | Student | Yes | N/A | N/A | Time |
| Lu, 2010 [44] | Time 1: 22.3 Time 2: 22.26  (Young) | Time 1: 59.35 Time 2: 66.67  (Majority female) | Student | Yes | 60  (Long) | 69.9  (Any) | Blood |
| Lu, Cheng, Lin, & Chen, 2019 [107] | 19.8  (Young) | 38.9  (Majority male) | Student | Yes | 28  (Short) | 11.3  (Any) | Time |
| MacGillivray & Lynd-Stevenson, 2013 [67] | 34.19  (Older) | 50  - | Non-student | Yes | N/A | N/A | Time |
| Mackay, White, & Obst, 2016 [94] | 25  (Older) | Time 1: 72  (Majority female)  Time 2: Unclear | Student | Yes | 28  (Short) | 43.6  (Any) | Time |
| Masser, Bednall, White, & Terry, 2012 [55] | Unclear | 79.13  (Majority female) | Non-student | No | 180  (Long) | 1  (Any) | Blood |
| Masser, Hyde, & Ferguson, 2020: Sample 1 (donors) [110] | Unclear | Unclear | Non-student | Yes | N/A | N/A | Blood |
| Masser, Hyde, & Ferguson, 2020: Sample 2 (non-donors) [110] | Unclear | Unclear | Non-student | Yes | N/A | N/A | Blood |
| Masser et al., 2009 [40] | Unclear | Time 1: 61.15 Time 2: 67.03  (Majority female) | Non-student | Yes | 90  (Long) | 30.8  (Any) | Blood |
| McGlade, McClenahan, & Pierscionek, 2012 [56] | 24  (Young) | 96.74  (Majority female) | Student | No | N/A | N/A | Organs |
| McMahon & Byrne, 2008 [34] | 29.88  (Older) | 73.26  (Majority female) | Non-student | Yes | 14  (Short) | 0  (No) | Blood |
| Meng, Chua, Ryu, & Han, 2020 [111] | 23.85  (Young) | 44.5  (Majority male) | Non-student | No* | N/A | N/A | Time |
| Meng, Ryu, Chua, & Han, 2020 [112] | 23.68  (Young) | 52.5  (Majority female) | Student | No | N/A | N/A | Time |
| Newton, Newton, Ewing, Burney, & Hay, 2013 [68] | 19.34  (Young) | 58.79  (Majority female) | Student | No | N/A | N/A | Organs |
| O'Brien, Fan, Yi, & Goldman, 2013 [70] | 33  (Older) | 40.49  (Majority male) | Non-student | Yes* | 730  (Long) | 0  (No) | Blood |
| O’Carroll, 2016: TPB/AR condition, time 1 measurement [95] | 45.62  (Older) | 65.61  (Majority female) | Non-student | Yes | 180  (Long) | 0  (No) | Organs |
| Park & Smith, 2007: registering behaviour [22] | 20.52  (Young) | 63.22  (Majority female) | Student | No | N/A | N/A | Organs |
| Park, Smith, & Yun, 2009 [42] | 47.40  (Older) | 49.2  (Majority male) | Non-student | No | N/A | N/A | Organs |
| Pavlova & Silbereisen, 2015a; 2015b: Civic volunteering [90, 91] | 29.9  (Older) | 48.1  (Majority male) | Non-student | No | N/A | N/A | Time |
| Pavlova & Silbereisen, 2015b:Political volunteering [91] | 29.8  (Older) | 48.3  (Majority male) | Non-student | No | N/A | N/A | Time |
| Polonsky, Renzaho, Ferdous, & McQuilten, 2013 [71] | 33  (Older) | 43.8  (Majority male) | Non-student | Yes | N/A | N/A | Blood |
| Poplaski, 2017: main study [99] | Unclear  (Older) | 60.8  (Female) | Non-student | Yes | N/A | N/A | Mixed |
| Reuveni & Werner, 2015 [92] | Unclear  (Young) | 46.09*  (Majority male) | Student | No | N/A | N/A | Time |
| Reynolds-Tylus & Quick, 2017 [100] | 20.64  (Young) | 59  (Majority female) | Non-student | No | N/A | N/A | Organs |
| Robinson, Masser, White, Hyde, & Terry, 2008 [35] | Unclear  (Older) | 77.37*  (Majority female) | Non-student | Yes | N/A | N/A | Blood |
| Siegel, Navarro, Tan, & Hyde, 2014: Study 2 [83] | 30.71  (Older) | 38.8  (Majority male) | Non-student | No | N/A | N/A | Organs |
| Smith & McSweeney, 2007 [23] | Time 1: 44.19 Time 2: 41.22  (Older) | Time 1: 73.57 Time 2: 61.19  (Majority female) | Non-student | Yes | 28  (Short) | 70.5  (Any) | Money |
| Stevenson, 2010 [45] | 32.1  (Older) | 63.49  (Majority female) | Non-student | Yes | N/A | N/A | Mixed |
| van der Linden, 2011 [50] | 28  (Older) | 56.64  (Majority female) | Non-student | Yes | N/A | N/A | Money |
| Veldhuizen, Atsma, van Dongen, & de Kort, 2012 [57] | 45.27  (Older) | 54.03  (Majority female) | Non-student | Yes* | N/A | N/A | Blood |
| Veldhuizen & van Dongen, 2013: Whole blood sample [73] | 34.3  (Older) | 69.12  (Majority female) | Non-student | No* | N/A | N/A | Blood |
| Veldhuizen & van Dongen, 2013: Plasma sample [73] | 36.4  (Older) | 66.2  (Majority female) | Non-student | No* | N/A | N/A | Blood |
| Veludo-de-Oliveira, Alhaidari, Yani-de-Soriano, & Yousafzai, 2017 [101] | Unclear  (Older) | 31.94  (Majority male) | Non-student | Yes* | 28  (Short) | 49  (Any) | Money |
| Veludo-de-Oliveira, Pallister, & Foxall, 2013 [74] | 20.3  (Young) | Time 1: 88.19 Time 2: 89.44  (Majority female) | Non-student | Yes | 150  (Long) | 32  (Any) | Time |
| Wang et al., 2011 [51] | 54.3  (Older) | 68.2  (Female) | Non-student | No | N/A | N/A | Time |
| Warburton & Terry, 2000 [12] | Unclear  (Older) | Time 1: 51.01 Time 2: 52.5  (Majority female) | Non-student | Yes | 35  (Short) | 18.9  (Any) | Time |
| Weber, Martin, & Corrigan, 2007 [24] | Unclear* | 50.41  (Majority female) | Student | No | 0  (Short) | 0  (No) | Organs |
| Weberling, 2011 [52] | 20  (Young) | 71.6  (Majority female) | Student | No | N/A | N/A | Time |
| White, Poulsen, & Hyde, 2017 [102] | 21.63  (Young) | Time 1: 76.35 Time 2: 78.64  (Majority female) | Student | Yes | 90  (Long) | 49  (Any) | Mixed |
| Yun & Park, 2010 [46] | 21  (Young) | 52.48  (Majority female) | Student | No | N/A | N/A | Organs |
| Zuckerman & Reis, 1978 [1] | Unclear | 46.22  (Majority male) | Student | Yes | 14  (Short) | 0  (No) | Blood |
| Coding agreement^1^ | 97.5% | 93.2% | 94.1% | 87.2% | 100 % | 69.7% | 100% |

*Note:* TA(C)T = Target, Action, (Context), Time. Please refer to Supplementary Table 1 for references.
