# Supplementary material for: Charitable donations and the theory of planned behaviour: A systematic review and meta-analysis
Source: PLoS One. 2023 May 19;18(5):e0286053. doi: 10.1371/journal.pone.0286053 (PMC10198540; doi:10.1371/journal.pone.0286053)
Supplement: S3 Table — (PDF) [file pone.0286053.s004.pdf]

Supplementary Table 3. Risk of Bias Assessment using JBI Checklist for Analytical Cross Sectional Studies									
Author and Year	Type of Record	1. Were the criteria for inclusion in the sample clearly defined?	2. Were the study subjects and the setting described in detail?	3. Was the exposure measured in a valid and reliable way?	4. Were objective, standard criteria used for measurement of the condition?	5. Were confounding factors identified?	6. Were strategies to deal with confounding factors stated?	7. Were the outcomes measured in a valid and reliable way?	8. Was appropriate statistical analysis used?
Zuckerman & Reis, 1978	Art	Y	N	UC	NA	NA	NA	Y	Y
Fortini, 1987	Art	Y	N	UC	NA	NA	NA	Y	Y
Charng, Piliavin & Callero, 1988	Art	Y	Y	Y	NA	NA	NA	Y	Y
Chafey, 1989	Diss	Y	Y	Y	NA	NA	NA	Y	Y
Borgida, Conner & Manteufel, 1992	BC	Y	Y	Y	NA	NA	NA	Y	Y
Jun, 1993	Diss	Y	Y	N	NA	NA	NA	Y	Y
Giles, 1992; Giles & Cairns, 1995	Diss, Art	Y	Y	Y	NA	NA	NA	Y	Y
Harrison, 1995; Harrison 1988	Art, Diss	Y	Y	Y	NA	NA	NA	Y	Y
Warburton, & Terry, 2000	Art	Y	Y	Y	NA	NA	NA	Y	Y
Armitage & Conner, 2001 (Study 1)	Art	Y	Y	Y	NA	NA	NA	NA	Y
Armitage & Conner, 2001 (Study 2)	Art	Y	Y	Y	NA	NA	NA	Y	Y
Amponsah-Afuwape, Myers, & Newman, 2002	Art	UC	Y	N	NA	NA	NA	NA	Y
Kidwell, & Jewell, 2003 (Study 1)	Art	Y	N	Y	NA	NA	NA	NA	Y
Giles, McClenahan, Cairns & Mallet, 2004	Art	Y	Y	Y	NA	NA	NA	Y	Y
Greenslade & White, 2005	Art	Y	Y	Y	NA	NA	NA	Y	Y
Holdershaw, 2005 (Main study)<br>Holdershaw, Gendall, & Wright, 2011	Diss, Art	Y	Y	Y	NA	NA	NA	Y	Y

Knowles 2005a; Knowles 2005b; Hyde Knowles & White, 2013	Ab, Ab, Art	Y	Y	Y	NA	NA	NA	NA	Y
Lemmens et al., 2005	Art	Y	Y	Y	NA	NA	NA	NA	Y
Hyde & White, 2006; Hyde & White 2009b	Ab, Art	Y	Y	Y	NA	NA	NA	Y	Y
Knowles, 2006a; Hyde, Knowles and Simon, 2013	Ab, Art	Y	Y	Y	NA	NA	NA	NA	Y
Knowles, 2006b; Knowles, Hyde and White, 2012	Ab, Art	Y	Y	Y	NA	NA	NA	NA	Y
Park & Smith, 2007	Art	Y	Y	Y	NA	NA	NA	NA	Y
Smith & McSweeney, 2007	Art	Y	Y	Y	NA	NA	NA	Y	Y
Weber, Martin & Corrigan, 2007	Art	Y	N	Y	NA	NA	NA	Y	Y
France, France & Himawan, 2007	Art	Y	Y	Y	NA	NA	NA	NA	Y
France, France & Himawan, 2008	Art	Y	Y	Y	NA	NA	NA	NA	Y
Masser, White, Robinson, Hyde & Terry, 2007; Robinson, Masser, White, Hyde & Terry, 2008	Rep, Art	Y	Y	Y	NA	NA	NA	Y	Y
Masser, White, Robinson, Hyde & Terry, 2007; Masser, White, Hyde, Terry & Robinson, 2009	Rep, Art	Y	Y	Y	NA	NA	NA	Y	Y
Bae, 2008; Bae & Kang, 2008	Art	Y	Y	Y	NA	NA	NA	NA	Y
Browne & Desmond, 2008	Art	Y	Y	Y	NA	NA	NA	NA	Y
Grano, Lucidi, Zelli & Violani, 2008	Art	Y	Y	Y	NA	NA	NA	NA	Y
Henning, 2008; Henning, Huffman & Elandt, 2009.	Diss, Ab	Y	Y	Y	NA	NA	NA	NA	Y
McMahon, & Byrne, 2008	Art	Y	Y	Y	NA	NA	NA	Y	Y
Hyde & White, 2009a	Art	Y	Y	Y	NA	NA	NA	NA	Y
Lemmens et al., 2009 (Study 1); (Study 2)	Art	N	Y	Y	NA	NA	NA	NA	Y
Park, Smith & Yun, 2009	Art	Y	Y	N	NA	NA	NA	NA	Y

Hyde & White, 2010	Art	Y	Y	Y	NA	NA	NA	Y	Y
Lu, 2010	Diss	Y	Y	Y	NA	NA	NA	Y	Y
Stevenson, 2010	Diss	Y	Y	N	NA	NA	NA	NA	Y
Yun & Park, 2010	Art	Y	Y	Y	NA	NA	NA	NA	Y
Kinnally & Brinkerhoff, 2011, 2013	Art	Y	Y	Y	NA	NA	NA	NA	Y
Lee, 2011	Diss	Y	Y	Y	NA	NA	NA	NA	Y
van der Linden, 2011	Art	Y	Y	Y	NA	NA	NA	NA	Y
Wang et al., 2011	Art	Y	Y	Y	NA	NA	NA	NA	Y
Weberling, 2011	Diss	Y	Y	Y	NA	NA	NA	NA	Y
Clowes & Masser, 2012	Art	Y	Y	Y	NA	N	NA	NA	Y
Masser, Bednall, White & Terry, 2012	Art	Y	Y	Y	NA	NA	NA	Y	Y
McGlade, McClenahan & Pierscionek, 2012	Art	Y	Y	Y	NA	NA	NA	NA	Y
Newton, Ewing, Burney & Hay, 2012	Art	Y	Y	Y	NA	NA	NA	NA	Y
Veldhuizen, Atsma, van Dongen & de Kort, 2012	Art	Y	Y	N	NA	NA	NA	NA	Y
Conner, Godin, Sheeran & Germain, 2013	Art	Y	N	Y	NA	NA	NA	Y	Y
Godin & Germain, 2013	Art	Y	Y	Y	NA	NA	NA	Y	Y
Hyde & White 2013a (Control Group)	Art	Y	Y	Y	NA	NA	NA	Y	Y
Hyde & White, 2013b	Art	Y	Y	Y	NA	NA	NA	Y	Y
Jiranek, Kals, Humm, Strubel & Wehner, 2013	Art	N	Y	Y	NA	NA	NA	NA	Y
MacGillivray, Lynd-Stevenson, 2013	Art	Y	Y	N	NA	NA	NA	N	Y
Newton, Newton, Ewing, Burney & Hay, 2013	Art	Y	Y	Y	NA	NA	NA	NA	Y
O'Brien, Fan, Yi & Goldman, 2013	Ab	N	Y	UC	NA	NA	NA	UC	UC
Polonsky, Renzaho, Ferdous & McQuilten, 2013; Ferdous, Polonsky, McQuilten & Renzaho, 2014	Art, BC	Y	Y	Y	NA	NA	NA	NA	Y

Veldhuizen & van Dongen, 2013	Art	Y	Y	N	NA	NA	NA	Y	Y
Veludo-de-Oliveira, 2009;<br>Veludo-de-Oliveira, Pallister & Foxall, 2013	Diss, Art	UC	Y	Y	NA	NA	NA	Y	Y
Alhidari, 2014	Diss	Y	Y	Y	NA	NA	NA	N	Y
Bang, Odio & Reio, 2014	Art	Y	Y	Y	NA	NA	NA	NA	Y
Evans & Ferguson, 2014	Art	Y	Y	Y	NA	NA	NA	NA	Y
France et al., 2014	Art	Y	Y	Y	NA	NA	NA	NA	Y
Huckins-Barker, 2014	Diss	Y	Y	Y	NA	NA	NA	NA	N
Lee, Won & Bang, 2014	Art	Y	Y	Y	NA	NA	NA	NA	Y
Siegel, Navarro, Tan & Hyde, 2014 (Study 2)	Art	N	Y	Y	NA	NA	NA	Y	Y
Brayley et al., 2015	Art	N	Y	Y	NA	NA	NA	NA	Y
Delaney & White, 2015	Art	Y	Y	Y	NA	NA	NA	Y	Y
Faqah, Moiz, Shahid, Ibrahim & Raheem, 2015	Art	Y	Y	N	NA	NA	NA	NA	Y
Kashif & De Run, 2015	Art	Y	Y	Y	NA	NA	NA	N	Y
Masser, Bagot, White & Bove, 2013;<br>Bagot, Masser & White, 2015	Ab, Art	Y	Y	Y	NA	NA	NA	Y	Y
Pavlova & Silbereisen, 2015a; 2015b	Art	Y	Y	Y	NA	NA	NA	N	Y
Reuveni & Werner, 2015	Art	Y	N	Y	NA	NA	NA	NA	Y
Charsetad, 2016	Art	Y	N	Y	NA	NA	NA	NA	Y
Mackay, White & Obst, 2016	Art	Y	Y	Y	NA	NA	NA	Y	Y
O'Carroll, Shepherd, Hayes & Eamonn, 2016<br>(Time 1)	Art	Y	Y	N	Y	Y	Y	Y	Y
Britt, Britt & Anderson, 2017	Art	Y	Y	Y	NA	NA	NA	NA	Y
Chen, 2017	Art	Y	Y	Y	NA	NA	NA	Y	Y

Hu, Wang & Fu, 2017	Art	Y	Y	Y	NA	NA	NA	Y	Y
Poplaski, 2017 (main study)	Diss	Y	Y	N	NA	NA	NA	NA	Y
Reynolds-Tylus & Quick, 2017	Art	Y	Y	Y	NA	NA	NA	NA	Y
Veludo-de-Oliveira, Alhaidari, Yani-de-Soriano & Yousafzai, 2017	Art	Y	Y	Y	NA	NA	NA	Y	Y
White, Poulsen & Hyde, 2017	Art	Y	Y	Y	NA	NA	NA	Y	Y
Fox, Himawan & France, 2018 (Study 2)	Art	Y	Y	Y	NA	NA	NA	NA	Y
Gellermann, 2018	Diss	Y	Y	Y	NA	NA	NA	NA	Y
Gilchrist, Masser, Horsley & Ditto, 2019	Art	Y	Y	Y	NA	NA	NA	NA	Y
Li & Wu, 2019	Art	Y	Y	Y	NA	NA	NA	NA	Y
Lu, Cheng, Lin & Chen, 2019	Art	Y	Y	Y	NA	NA	NA	Y	Y
Alsalem et al., 2020	Art	Y	Y	Y	NA	NA	NA	NA	Y
Costa et al., 2020	Art	Y	Y	Y	NA	NA	NA	NA	Y
Masser et al., 2020. Sample 1 (donors); Sample 2 (non-donors)	Art	Y	N	Y	NA	NA	NA	NA	Y
Meng et al., 2020. Journal of Responsible Tourism	Art	N	Y	Y	NA	NA	NA	NA	Y
Meng et al., 2020. Asia Pacific Journal of Tourism Research	Art	N	Y	Y	NA	NA	NA	NA	Y
Duh & Dabula, 2021	Art	Y	Y	Y	NA	NA	NA	NA	Y
Fernandes et al., 2021 Sample 1 (multiparous); Sample 2 (primiparous)	Art	Y	Y	Y	NA	NA	NA	NA	Y
Chetioui et al., 2022	Art	N	N	Y	NA	NA	NA	NA	Y
Li et al. 2022	Art	Y	Y	Y	NA	NA	NA	NA	Y

Note. Refer to http://joannabriggs.org/research/critical-appraisal-tools.html for the questions and how each survey was evaluated. The following parameters were used by the authors to ensure consistent assessment; Q1: general inclusion/exclusion; Q2: As long as demographics of age and sex then a "YES"; bonus if also mention ethnicity/SES/other or past giving or time period (as rare) - same for location as not always stipulated explicitly; Q3: for IVs if based on other studies/Ajzen website etc deemed valid (or if piloted for validity etc) and if offer reliability statistics that meet criteria (such as most scales >.7 Cronbach or >.4/.5 correlation) then "YES". Some difficulty as articles report scales are reliable with a Cronbach cut-off of .60 so treated as reliable; Q4: only answered in relation to RCTs (re matching etc); Q5: only answered in relation to RCTs (re identifying differences between comparison groups such as baseline characteristics); Q6: only answered in relation to RCTs re dealing with confounders; Q7: for DVs of behaviour YES if observational/objective or for self-report as long as valid/reliable; Q8: check for correlations/regressions/path analysis coefficients provided etc (and assumptions as a bonus) then "YES".

Y=Yes; N=No; UC=Unclear; Ab=Abstract, Art=Article, BC=Book Chapter, Conf_Pr = Conference Presentation, Diss = Dissertation, Rep = Report.
