# Supplementary material for: Charitable donations and the theory of planned behaviour: A systematic review and meta-analysis
Source: PLoS One. 2023 May 19;18(5):e0286053. doi: 10.1371/journal.pone.0286053 (PMC10198540; doi:10.1371/journal.pone.0286053)
Supplement: S1 File — Forest and Funnel plots. (PDF) [file pone.0286053.s005.pdf]

Supplementary Figures

1. Forest plot for the attitude-intention association
2. Forest plot for the subjective norm-intention association
3. Forest plot for the PBC-intention association
4. Forest plot for the moral norm-intention association
5. Forest plot for the PBC-behaviour association
6. Forest plot for the intention-behaviour association
7. Funnel plots of standard error by Fisher's Z

1. Forest plot for the attitude-intention association

Study name	Correlation	Lower limit	Upper limit	Z-Value	p-Value
	Statistics for each study				
Aji, H et al., 2021	0.768	0.732	0.800	23.965	0.000
Alhidari, 2014	0.530	0.459	0.595	12.223	0.000
Alsalem, A et al., 2020	0.770	0.728	0.806	20.910	0.000
Amponsah-Afuwape et al., 2002	0.460	0.322	0.579	5.947	0.000
Armitage & Conner, 2001: Study 1	0.680	0.577	0.762	9.490	0.000
Armitage & Conner, 2001: Study 2	0.530	0.413	0.630	7.672	0.000
Aschale, A., 2021	0.458	0.392	0.519	12.038	0.000
Bae & Kang, 2008	0.650	0.624	0.674	35.146	0.000
Bagot, Masser, & White, 2015	0.430	0.358	0.497	10.528	0.000
Bagot, Masser, & White, 2015	0.440	0.308	0.555	6.029	0.000
Bang, Odio, & Reio, 2014	0.270	0.084	0.437	2.823	0.005
Borgida, Conner, & Manteufel, 1992	0.390	0.266	0.501	5.795	0.000
Brayley, Obst, White, Lewis, Warburton, & Spencer, 2015	0.590	0.488	0.676	9.167	0.000
Browne & Desmond, 2008	0.609	0.491	0.705	8.127	0.000
Chafey, 1989	0.830	0.737	0.892	9.505	0.000
Charng, Piliavin, & Callero, 1988	0.240	0.167	0.311	6.264	0.000
Chen, 2017	0.440	0.323	0.544	6.761	0.000
Chetioui, Y,et al. 2021	0.677	0.618	0.728	15.927	0.000
Clowes & Masser, 2012	0.548	0.368	0.688	5.259	0.000
Conner, Godin, Sheeran, & Germain, 2013	0.378	0.326	0.427	13.202	0.000
Costa, A et al. 2020	0.496	0.384	0.594	7.635	0.000
Delaney & White, 2015	0.620	0.532	0.695	10.705	0.000
Duh & Dabula, 2021	0.525	0.467	0.579	14.835	0.000
Evans & Ferguson, 2014	0.405	0.321	0.483	8.667	0.000
Faqah, Moiz, Shahid, Ibrahim, & Raheem, 2015	0.723	0.672	0.767	18.002	0.000
Fernandes et al., 2021; Sample 1 (Multiparous)	0.520	0.351	0.656	5.376	0.000
Fernandes et al., 2021; Sample 2 (Primiparous)	0.690	0.623	0.747	13.985	0.000
Fortini, 1987	0.818	0.753	0.867	13.221	0.000
Fox, Himawan, & France, 2018: Study 2	0.575	0.484	0.654	10.104	0.000
France et al., 2014	0.520	0.448	0.586	11.951	0.000
France, France, & Himawan, 2008	0.669	0.615	0.716	17.368	0.000
Gellermann, 2018	0.396	0.367	0.425	23.800	0.000
Gilchrist, Masser, Horsley, & Ditto, 2019	0.620	0.551	0.681	13.447	0.000
Giles & Cairns, 1995	0.550	0.425	0.655	7.343	0.000
Giles, McClenahan, Cairns, & Mallet, 2004	0.250	0.056	0.426	2.516	0.012
Godin & Germain, 2013	0.636	0.556	0.704	11.857	0.000
Grano, Lucidi, Zelli, & Violani, 2008	0.190	0.113	0.265	4.758	0.000
Greenslade & White, 2005	0.670	0.565	0.754	9.315	0.000
Harrison, 1995 s1	0.320	0.043	0.552	2.249	0.024
Harrison, 1995 s2	0.215	-0.067	0.465	1.497	0.134
Harrison, 1995 s3	0.400	0.134	0.612	2.873	0.004
Henning, 2008	0.450	0.287	0.588	5.014	0.000
Holdershaw, 2005: main study	0.590	0.553	0.625	24.045	0.000
Holdershaw, 2005: pilot study	0.370	0.187	0.528	3.826	0.000
Hossain Parash, Suki, Shimmi, Hossain, & Murthy, 2020	0.580	0.516	0.638	14.084	0.000
Hu, Wang, & Fu, 2017: Sample 2	0.350	0.290	0.407	10.704	0.000
Huckins-Barker, 2014	0.670	0.611	0.721	15.949	0.000
Hughes, 1984 s1	0.707	0.372	0.879	3.525	0.000
Hughes, 1984 s2	0.530	0.239	0.734	3.338	0.001
Hughes, 1984 s3	0.270	-0.281	0.687	0.959	0.338
Hughes, 1984 s4	0.288	-0.191	0.656	1.186	0.236
Hyde & White, 2009a: registering behaviour	0.610	0.541	0.671	13.376	0.000
Hyde & White, 2009b: registering behaviour	0.560	0.477	0.633	10.961	0.000
Hyde & White, 2010: registering behaviour	0.550	0.471	0.620	11.335	0.000
Hyde & White, 2013a: registering behaviour, control group	0.636	0.439	0.774	5.260	0.000
Hyde & White, 2013b	0.390	0.256	0.509	5.385	0.000
Hyde, Knowles, & White, 2013/Hyde & Knowles, 2013 b	0.730	0.667	0.782	14.831	0.000
Hyde, Knowles, & White, 2013/Hyde & Knowles, 2013 o	0.740	0.679	0.791	15.178	0.000
Hyde, Knowles, & White, 2013/Hyde & Knowles, 2013 v	0.640	0.558	0.710	11.548	0.000
Jun, 1993 ko	0.390	0.257	0.509	5.416	0.000
Jun, 1993 po	0.330	0.270	0.387	10.187	0.000
Jun, 1993 us	0.245	0.172	0.316	6.400	0.000
Kashif & De Run, 2015	0.780	0.723	0.827	15.505	0.000
Kassie, Azale, & Nigusie, 2020	0.400	0.325	0.470	9.586	0.000
Kidwell & Jewell, 2003: Study 1, blood donation behaviour	0.475	0.335	0.594	6.023	0.000
Kim & Lee, 2014	0.380	0.277	0.474	6.777	0.000
Kinnally & Brinkerhoff, 2011	0.380	0.310	0.446	9.848	0.000
Knowles, Hyde, & White, 2012	0.650	0.564	0.722	11.155	0.000
Lee, Won, & Bang, 2014	0.150	0.029	0.266	2.432	0.015
Lemmens et al., 2005	0.490	0.396	0.574	8.986	0.000
Lemmens et al., 2009: Study 1	0.425	0.317	0.522	7.074	0.000
Lemmens et al., 2009: Study 2	0.330	0.261	0.395	8.907	0.000
Li & Wu, 2019	0.670	0.600	0.730	13.591	0.000
Li et al., 2022	0.560	0.508	0.608	16.957	0.000
Lu, 2010	0.330	0.213	0.437	5.322	0.000
Lu, Cheng, Lin, & Chen, 2019	0.260	0.159	0.356	4.936	0.000
MacGillvray & Lynd-Stevenson, 2013	0.470	0.332	0.588	6.057	0.000
Mackay, White, & Obst, 2016	0.640	0.568	0.702	13.132	0.000
Masser el al., 2009	0.580	0.473	0.670	8.764	0.000
Masser et al., 2020; Sample 1 (donors)	0.320	0.209	0.423	5.439	0.000
Masser et al., 2020; Sample 2 (non-donors)	0.200	0.074	0.320	3.088	0.002
Masser, Bednall, White, & Terry, 2012	0.485	0.385	0.574	8.372	0.000
McGlade, McClenahan, & Pierscionek, 2012	0.910	0.867	0.940	14.411	0.000
McMahon & Byrne, 2008	0.135	-0.015	0.279	1.766	0.077
Meng et al., 2020; Asia Pacific Journal of Tourism Research	0.616	0.543	0.680	12.793	0.000
Meng et al., 2020; Journal of Sustainable Tourism	0.510	0.431	0.581	10.854	0.000
Newton et al., 2013	0.600	0.529	0.663	12.949	0.000
O'Carroll, 2016: TPB/AR condition, time 1 measurement	0.440	0.362	0.512	9.939	0.000
O'Brien, Fan, Yi, & Goldman, 2013	0.250	0.168	0.329	5.807	0.000
Park & Smith, 2007: registering behaviour	0.560	0.468	0.640	9.865	0.000
Park, Smith, & Yun, 2009	0.500	0.467	0.531	25.154	0.000
Pavlova & Silbereisen, 2015 c	0.165	0.091	0.237	4.361	0.000
Pavlova & Silbereisen, 2015 p	0.283	0.213	0.351	7.592	0.000
Polonsky, Renzaho, Ferdous, & McQuilten, 2013	0.620	0.558	0.675	14.894	0.000
Poplaski, 2017: main study	0.270	0.151	0.381	4.351	0.000
Reuveni & Werner, 2015	0.590	0.504	0.664	10.821	0.000
Reynolds-Tylus & Quick, 2017	0.540	0.456	0.615	10.534	0.000
Robinson et al., 2008	0.680	0.596	0.749	11.489	0.000
Siegel, Navarro, Tan, & Hyde, 2014: Study 2	0.650	0.586	0.706	14.608	0.000
Smith & McSweeney, 2007	0.360	0.241	0.468	5.641	0.000
Stevenson, 2010	0.400	0.303	0.489	7.483	0.000
van der Linden, 2011	0.500	0.366	0.614	6.499	0.000
Veldhuizen & van Dongen, 2013 p	0.400	0.283	0.506	6.241	0.000
Veldhuizen & van Dongen, 2013 w	0.345	0.308	0.381	17.034	0.000
Veldhuizen, Atsma, van Dongen, & de Kort, 2012	0.230	0.213	0.247	25.705	0.000
Veludo-de-Oliveira, Alhaidari, Yani-de-Soriano, & Yousafzai, 2017	0.550	0.481	0.613	12.808	0.000
Veludo-de-Oliveira, Pallister, & Foxall, 2013	-0.087	-0.212	0.041	-1.334	0.182
Warburton & Terry, 2000	0.330	0.224	0.428	5.868	0.000
Weber, Martin, & Corrigan, 2007	0.470	0.387	0.546	9.772	0.000
White, Poulsen, & Hyde, 2017	0.493	0.377	0.594	7.385	0.000
Yun & Park, 2010	0.530	0.469	0.586	14.200	0.000
Zuckerman & Reis, 1978	0.380	0.269	0.481	6.300	0.000
	0.435	0.428	0.442	109.581	0.000

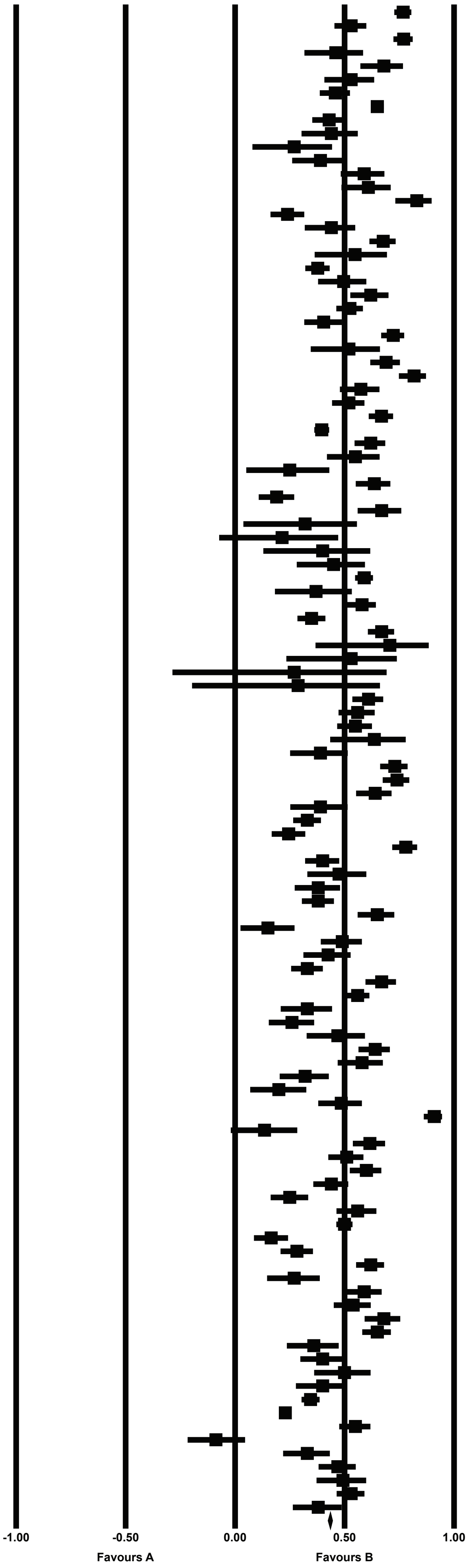

2. Forest plot for the subjective norm-intention association

Study name	Correlation	Lower limit	Upper limit	Z-Value	p-Value
Aji, H et al., 2021	0.704	0.660	0.743	20.655	0.000
Alhidari, 2014	0.560	0.492	0.622	13.107	0.000
Alsalem, A et al., 2020	0.290	0.200	0.375	6.119	0.000
Amponsah-Afuwape et al., 2002	0.360	0.210	0.494	4.507	0.000
Armitage & Conner, 2001: Study 1	0.370	0.214	0.508	4.446	0.000
Armitage & Conner, 2001: Study 2	0.320	0.179	0.448	4.311	0.000
Aschale, A., 2021	0.477	0.412	0.537	12.630	0.000
Bae & Kang, 2008	0.720	0.699	0.740	41.145	0.000
Bagot, Masser, & White, 2015	0.390	0.315	0.460	9.427	0.000
Bagot, Masser, & White, 2015	0.450	0.320	0.564	6.188	0.000
Bang, Odio, & Reio, 2014	0.670	0.550	0.763	8.268	0.000
Borgida, Conner, & Manteufel, 1992	0.120	-0.019	0.254	1.697	0.090
Brayley, Obst, White, Lewis, Warburton, & Spencer, 2015	0.660	0.570	0.734	10.725	0.000
Britt, Britt, & Anderson, 2017	0.242	0.085	0.387	2.993	0.003
Browne & Desmond, 2008	0.293	0.131	0.440	3.468	0.001
Chafey, 1989	0.340	0.109	0.536	2.833	0.005
Charsetad, 2016	0.170	0.045	0.290	2.654	0.008
Chen, 2017	0.430	0.312	0.535	6.585	0.000
Chetioui, Y,et al. 2021	0.619	0.553	0.678	13.990	0.000
Clowes & Masser, 2012	0.282	0.060	0.477	2.477	0.013
Conner, Godin, Sheeran, & Germain, 2013	0.357	0.305	0.407	12.414	0.000
Costa, A et al. 2020	0.354	0.226	0.470	5.193	0.000
Delaney & White, 2015	0.500	0.394	0.593	8.110	0.000
Evans & Ferguson, 2014	0.575	0.506	0.636	13.213	0.000
Faqah, Moiz, Shahid, Ibrahim, & Raheem, 2015	0.304	0.211	0.391	6.184	0.000
Fernandes et al., 2021; Sample 1 (Multiparous)	0.660	0.525	0.763	7.395	0.000
Fernandes et al., 2021; Sample 2 (Primiparous)	0.870	0.838	0.896	21.986	0.000
Fortini, 1987	0.693	0.594	0.772	9.808	0.000
France et al., 2014	0.350	0.264	0.430	7.578	0.000
France, France, & Himawan, 2008	0.484	0.411	0.551	11.341	0.000
Gellermann, 2018	0.873	0.865	0.881	76.448	0.000
Gilchrist, Masser, Horsley, & Ditto, 2019	0.355	0.259	0.444	6.884	0.000
Giles & Cairns, 1995	0.220	0.059	0.370	2.656	0.008
Giles, McClenahan, Cairns, & Mallet, 2004	0.180	-0.017	0.364	1.792	0.073
Godin & Germain, 2013	0.510	0.412	0.596	8.880	0.000
Grano, Lucidi, Zelli, & Violani, 2008	0.460	0.395	0.520	12.303	0.000
Greenslade & White, 2005	0.510	0.375	0.624	6.563	0.000
Harrison, 1995	0.540	0.305	0.713	4.098	0.000
Harrison, 1995	0.430	0.172	0.633	3.153	0.002
Harrison, 1995	0.480	0.230	0.671	3.547	0.000
Henning, 2008	0.450	0.287	0.588	5.014	0.000
Holdershaw, 2005: main study	0.360	0.311	0.407	13.373	0.000
Holdershaw, 2005: pilot study	0.370	0.187	0.528	3.826	0.000
Hossain Parash, Suki, Shimmi, Hossain, & Murthy, 2020	0.542	0.474	0.604	12.905	0.000
Hu, Wang, & Fu, 2017: Sample 2	0.320	0.259	0.379	9.714	0.000
Hyde & White, 2009a: registering behaviour	0.740	0.689	0.783	17.934	0.000
Hyde & White, 2009b: registering behaviour	0.430	0.333	0.518	7.966	0.000
Hyde & White, 2010: registering behaviour	0.700	0.641	0.751	15.898	0.000
Hyde & White, 2013a: registering behaviour, control group	0.782	0.647	0.869	7.353	0.000
Hyde & White, 2013b	0.510	0.391	0.612	7.359	0.000
Hyde, Knowles, & White, 2013/Hyde & Knowles, 2013	0.520	0.424	0.605	9.065	0.000
Jiranek et al., 2013	0.310	0.229	0.387	7.160	0.000
Kashif & De Run, 2015	0.720	0.650	0.778	13.463	0.000
Kidwell & Jewell, 2003: Study 1, blood donation behaviour	0.368	0.215	0.504	4.503	0.000
Kim & Lee, 2014	0.720	0.660	0.771	15.376	0.000
Kinnally & Brinkerhoff, 2011	0.290	0.216	0.361	7.350	0.000
Knowles, Hyde, & White, 2012	0.450	0.335	0.552	6.974	0.000
Lee, 2011	0.164	0.050	0.274	2.809	0.005
Lee, Won, & Bang, 2014	0.180	0.060	0.295	2.929	0.003
Lemmens et al., 2005	0.360	0.254	0.457	6.318	0.000
Lemmens et al., 2009: Study 1	0.360	0.246	0.464	5.875	0.000
Lemmens et al., 2009: Study 2	0.360	0.293	0.424	9.792	0.000
Li & Wu, 2019	0.400	0.297	0.493	7.102	0.000
Li et al., 2022	0.480	0.422	0.534	14.014	0.000
Lu, 2010	0.315	0.197	0.424	5.062	0.000
Lu, Cheng, Lin, & Chen, 2019	0.290	0.191	0.384	5.538	0.000
MacGillvray & Lynd-Stevenson, 2013	0.470	0.332	0.588	6.057	0.000
Mackay, White, & Obst, 2016	0.570	0.489	0.641	11.215	0.000
Masser el al., 2009	0.440	0.313	0.551	6.247	0.000
Masser et al., 2020; Sample 1 (donors)	0.470	0.372	0.558	8.366	0.000
Masser et al., 2020; Sample 2 (non-donors)	0.470	0.364	0.564	7.769	0.000
Masser, Bednall, White, & Terry, 2012	0.400	0.291	0.499	6.698	0.000
McGlade, McClenahan, & Pierscionek, 2012	0.670	0.539	0.769	7.649	0.000
McMahon & Byrne, 2008	-0.180	-0.321	-0.031	-2.366	0.018
Meng et al., 2020; Asia Pacific Journal of Tourism Research	0.365	0.266	0.456	6.813	0.000
Meng et al., 2020; Journal of Sustainable Tourism	0.580	0.509	0.643	12.777	0.000
Newton et al., 2013	0.690	0.631	0.741	15.841	0.000
O'Carroll, 2016: TPB/AR condition, time 1 measurement	0.560	0.493	0.621	13.320	0.000
O'Brien, Fan, Yi, & Goldman, 2013	0.225	0.142	0.305	5.205	0.000
Park & Smith, 2007: registering behaviour	0.490	0.389	0.580	8.356	0.000
Park, Smith, & Yun, 2009	0.560	0.530	0.589	28.979	0.000
Pavlova & Silbereisen, 2015	0.617	0.568	0.662	18.517	0.000
Polonsky, Renzaho, Ferdous, & McQuilten, 2013	0.250	0.159	0.337	5.247	0.000
Poplaski, 2017: main study	0.760	0.702	0.808	15.657	0.000
Reynolds-Tylus & Quick, 2017	0.597	0.520	0.665	12.004	0.000
Robinson et al., 2008	0.610	0.514	0.691	9.823	0.000
Smith & McSweeney, 2007	0.295	0.171	0.409	4.550	0.000
Stevenson, 2010	0.510	0.423	0.587	9.940	0.000
van der Linden, 2011	0.255	0.095	0.402	3.085	0.002
Veldhuizen & van Dongen, 2013	0.200	0.070	0.324	2.986	0.003
Veldhuizen & van Dongen, 2013	0.140	0.099	0.180	6.673	0.000
Veldhuizen, Atsma, van Dongen, & de Kort, 2012	0.260	0.243	0.277	29.209	0.000
Veludo-de-Oliveira, Alhaidari, Yani-de-Soriano, & Yousafzai, 2017	0.570	0.503	0.630	13.412	0.000
Veludo-de-Oliveira, Pallister, & Foxall, 2013	0.415	0.304	0.515	6.756	0.000
Warburton & Terry, 2000	0.540	0.454	0.616	10.341	0.000
Weber, Martin, & Corrigan, 2007	0.510	0.430	0.582	10.780	0.000
Weberling, 2011	0.580	0.520	0.635	14.975	0.000
White, Poulsen, & Hyde, 2017	0.580	0.477	0.667	9.059	0.000
Yun & Park, 2010	0.540	0.480	0.595	14.537	0.000
Zuckerman & Reis, 1978	0.340	0.226	0.445	5.576	0.000
	0.465	0.459	0.472	113.755	0.000

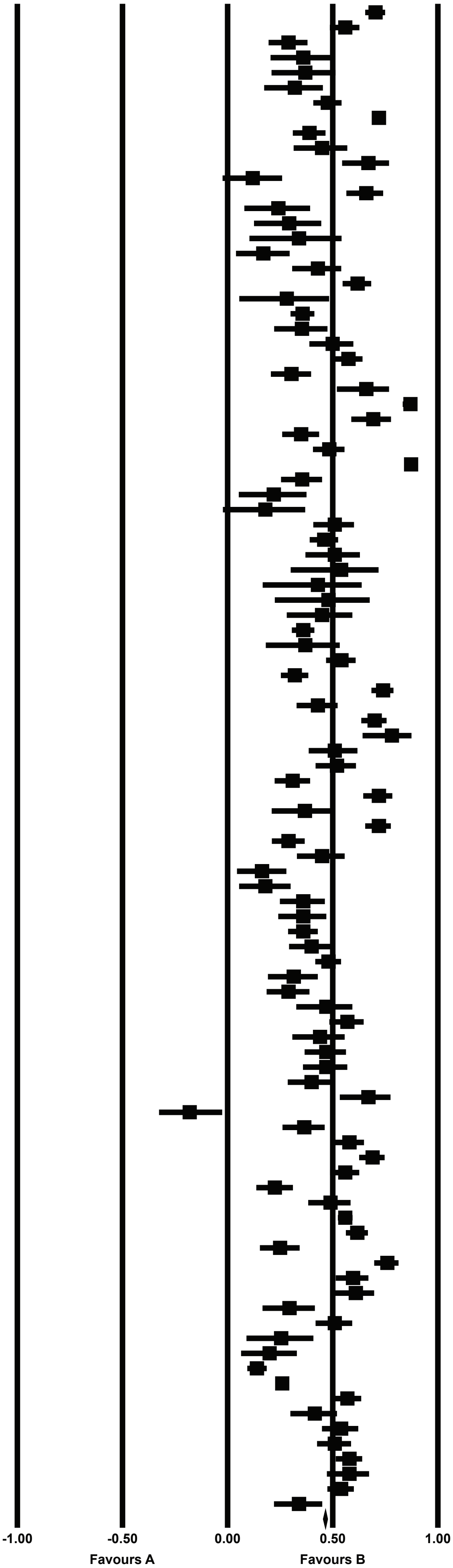

3. Forest plot for the PBC-intention association

Study name	Correlation	Lower limit	Upper limit	Z-Value	p-Value
Alhidari, 2014	0.680	0.626	0.728	17.173	0.000
Amponsah-Afuwape et al., 2002	0.600	0.485	0.695	8.289	0.000
Armitage & Conner, 2001: Study 1	0.370	0.214	0.508	4.446	0.000
Armitage & Conner, 2001: Study 2	0.505	0.384	0.609	7.228	0.000
Aschale, A., 2021	0.764	0.728	0.796	24.471	0.000
Bae & Kang, 2008	0.790	0.773	0.806	48.570	0.000
Bagot, Masser, & White, 2015	0.560	0.498	0.616	14.486	0.000
Bagot, Masser, & White, 2015	0.550	0.434	0.648	7.895	0.000
Bang, Odio, & Reio, 2014	0.280	0.095	0.446	2.934	0.003
Borgida, Conner, & Manteufel, 1992	0.590	0.492	0.673	9.536	0.000
Brayley, Obst, White, Lewis, Warburton, & Spencer, 2015	0.640	0.546	0.718	10.256	0.000
Browne & Desmond, 2008	0.494	0.355	0.612	6.219	0.000
Charsetad, 2016	0.250	0.128	0.365	3.949	0.000
Chen, 2017	0.350	0.225	0.464	5.232	0.000
Chetioui, Y,et al. 2021	0.517	0.439	0.587	11.067	0.000
Conner, Godin, Sheeran, & Germain, 2013	0.576	0.535	0.614	21.822	0.000
Costa, A et al. 2020	0.718	0.643	0.779	12.681	0.000
Delaney & White, 2015	-0.050	-0.181	0.083	-0.739	0.460
Evans & Ferguson, 2014	0.350	0.262	0.432	7.373	0.000
Faqah, Moiz, Shahid, Ibrahim, & Raheem, 2015	0.736	0.687	0.778	18.549	0.000
Fernandes et al., 2021; Sample 1 (Multiparous)	0.440	0.256	0.593	4.405	0.000
Fernandes et al., 2021; Sample 2 (Primiparous)	0.520	0.428	0.601	9.505	0.000
Fox, Himawan, & France, 2018: Study 2	0.370	0.256	0.474	5.992	0.000
France et al., 2014	0.740	0.694	0.780	19.710	0.000
France, France, & Himawan, 2008	0.687	0.636	0.732	18.084	0.000
Gellermann, 2018	0.910	0.903	0.915	86.622	0.000
Gilchrist, Masser, Horsley, & Ditto, 2019	0.575	0.500	0.641	12.148	0.000
Giles & Cairns, 1995	0.730	0.643	0.798	11.028	0.000
Giles, McClenahan, Cairns, & Mallet, 2004	0.600	0.457	0.712	6.827	0.000
Godin & Germain, 2013	0.707	0.639	0.764	13.904	0.000
Grano, Lucidi, Zelli, & Violani, 2008	0.740	0.702	0.774	23.514	0.000
Greenslade & White, 2005	0.810	0.744	0.861	13.095	0.000
Harrison, 1995	0.370	0.099	0.590	2.634	0.008
Harrison, 1995	0.480	0.233	0.669	3.585	0.000
Harrison, 1995	0.500	0.255	0.685	3.726	0.000
Henning, 2008	0.580	0.441	0.692	6.853	0.000
Holdershaw, 2005: main study	0.660	0.628	0.690	28.131	0.000
Holdershaw, 2005: pilot study	0.700	0.584	0.788	8.542	0.000
Hossain Parash, Suki, Shimmi, Hossain, & Murthy, 2020	0.680	0.627	0.727	17.627	0.000
Hu, Wang, & Fu, 2017: Sample 2	0.340	0.280	0.398	10.372	0.000
Hyde & White, 2009a: registering behaviour	0.700	0.643	0.749	16.364	0.000
Hyde & White, 2009b: registering behaviour	0.570	0.489	0.641	11.215	0.000
Hyde & White, 2010: registering behaviour	0.670	0.607	0.725	14.861	0.000
Hyde & White, 2013a: registering behaviour, control group	0.576	0.360	0.734	4.595	0.000
Hyde & White, 2013b	0.260	0.116	0.394	3.480	0.001
Hyde, Knowles, & White, 2013/Hyde & Knowles, 2013	0.766	0.709	0.813	15.872	0.000
Jiranek et al., 2013	0.220	0.135	0.302	4.996	0.000
Kashif & De Run, 2015	0.920	0.897	0.938	23.569	0.000
Kidwell & Jewell, 2003: Study 1, blood donation behaviour	0.465	0.324	0.586	5.874	0.000
Kinnally & Brinkerhoff, 2011	0.240	0.164	0.313	6.026	0.000
Knowles, Hyde, & White, 2012	0.630	0.541	0.705	10.667	0.000
Lee, Won, & Bang, 2014	0.630	0.551	0.698	11.932	0.000
Lemmens et al., 2005	0.350	0.244	0.448	6.126	0.000
Lemmens et al., 2009: Study 1	0.380	0.268	0.482	6.236	0.000
Lemmens et al., 2009: Study 2	0.440	0.377	0.499	12.269	0.000
Li & Wu, 2019	0.710	0.647	0.763	14.872	0.000
Li et al., 2022	0.340	0.274	0.403	9.488	0.000
Lu, 2010	0.395	0.283	0.496	6.485	0.000
Lu, Cheng, Lin, & Chen, 2019	0.280	0.180	0.374	5.336	0.000
MacGillvray & Lynd-Stevenson, 2013	0.480	0.343	0.597	6.210	0.000
Mackay, White, & Obst, 2016	0.470	0.377	0.553	8.835	0.000
Masser el al., 2009	0.690	0.604	0.760	11.217	0.000
Masser et al., 2020; Sample 1 (donors)	0.680	0.610	0.739	13.598	0.000
Masser et al., 2020; Sample 2 (non-donors)	0.600	0.511	0.676	10.558	0.000
Masser, Bednall, White, & Terry, 2012	0.450	0.346	0.543	7.664	0.000
McGlade, McClenahan, & Pierscionek, 2012	0.600	0.451	0.717	6.539	0.000
McMahon & Byrne, 2008	0.430	0.300	0.545	5.979	0.000
Meng et al., 2020; Asia Pacific Journal of Tourism Research	0.204	0.097	0.307	3.684	0.000
Meng et al., 2020; Journal of Sustainable Tourism	0.520	0.442	0.590	11.116	0.000
Newton et al., 2013	0.320	0.223	0.411	6.196	0.000
O'Carroll, 2016: TPB/AR condition, time 1 measurement	0.570	0.504	0.630	13.629	0.000
O'Brien, Fan, Yi, & Goldman, 2013	0.300	0.220	0.376	7.038	0.000
Park & Smith, 2007: registering behaviour	0.320	0.203	0.428	5.159	0.000
Park, Smith, & Yun, 2009	0.350	0.312	0.387	16.735	0.000
Pavlova & Silbereisen, 2015	0.622	0.573	0.667	18.693	0.000
Polonsky, Renzaho, Ferdous, & McQuilten, 2013	0.740	0.694	0.780	19.525	0.000
Poplaski, 2017: main study	0.510	0.412	0.596	8.844	0.000
Reynolds-Tylus & Quick, 2017	0.323	0.219	0.420	5.841	0.000
Robinson et al., 2008	0.190	0.051	0.322	2.665	0.008
Smith & McSweeney, 2007	0.620	0.533	0.694	10.851	0.000
Stevenson, 2010	0.300	0.196	0.397	5.467	0.000
van der Linden, 2011	0.290	0.132	0.434	3.533	0.000
Veldhuizen & van Dongen, 2013	0.670	0.590	0.737	11.943	0.000
Veldhuizen & van Dongen, 2013	0.730	0.710	0.749	43.975	0.000
Veldhuizen, Atsma, van Dongen, & de Kort, 2012	0.710	0.701	0.719	97.380	0.000
Veludo-de-Oliveira, Alhaidari, Yani-de-Soriano, & Yousafzai, 2017	0.700	0.648	0.745	17.964	0.000
Veludo-de-Oliveira, Pallister, & Foxall, 2013	0.771	0.714	0.818	15.646	0.000
Wang et al., 2011	0.563	0.460	0.651	8.898	0.000
Warburton & Terry, 2000	0.720	0.660	0.771	15.536	0.000
White, Poulsen, & Hyde, 2017	0.640	0.548	0.717	10.368	0.000
Yun & Park, 2010	0.460	0.393	0.522	11.967	0.000
	0.647	0.642	0.652	169.136	0.000

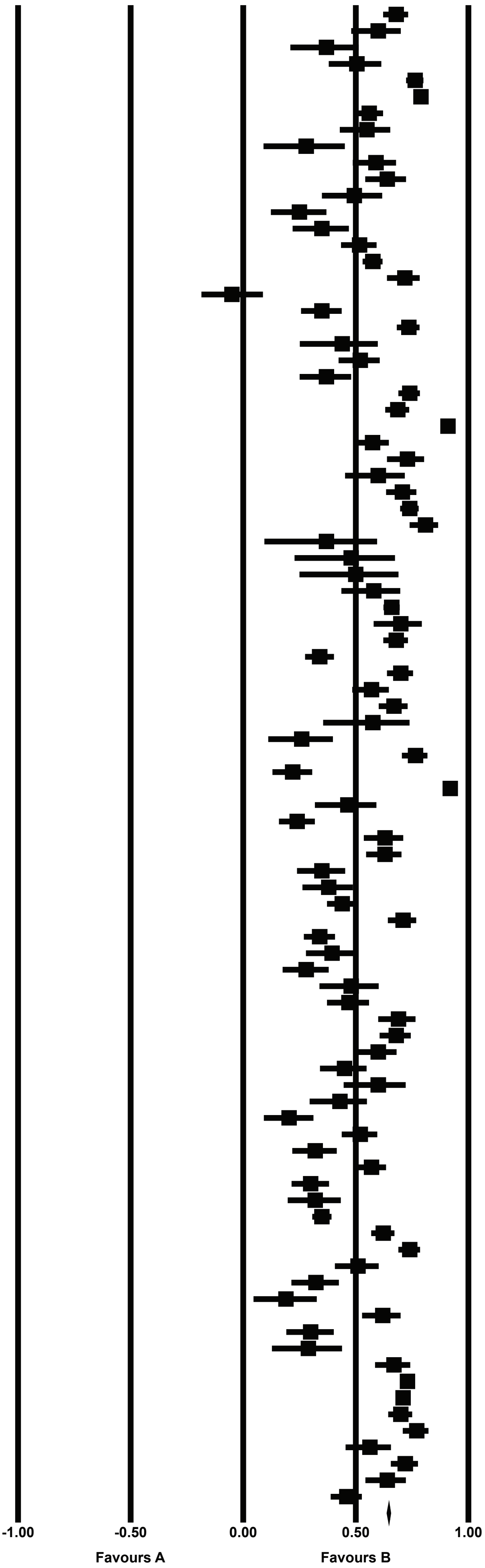

4. Forest plot for the moral norm-intention association

Study name	Correlation	Lower limit	Upper limit	Z-Value	p-Value
Alhidari, 2014	0.680	0.626	0.728	17.173	0.000
Armitage & Conner, 2001: Study 2	0.430	0.300	0.545	5.979	0.000
Bagot, Masser, & White, 2015	0.640	0.587	0.688	17.355	0.000
Bagot, Masser, & White, 2015	0.540	0.422	0.640	7.713	0.000
Bang, Odio, & Reio, 2014	0.520	0.366	0.646	5.878	0.000
Chafey, 1989	0.580	0.395	0.720	5.300	0.000
Costa, A et al. 2020	0.619	0.525	0.698	10.153	0.000
Delaney & White, 2015	0.740	0.674	0.794	14.034	0.000
Faqah, Moiz, Shahid, Ibrahim, & Raheem, 2015	0.732	0.682	0.775	18.378	0.000
France, France, & Himawan, 2008	0.354	0.272	0.431	7.944	0.000
Gilchrist, Masser, Horsley, & Ditto, 2019	0.450	0.362	0.530	8.990	0.000
Harrison, 1995	0.770	0.624	0.864	6.920	0.000
Harrison, 1995	0.560	0.334	0.725	4.338	0.000
Harrison, 1995	0.450	0.193	0.649	3.287	0.001
Holdershaw, 2005: main study	0.500	0.457	0.540	19.491	0.000
Hyde & White, 2009a: registering behaviour	0.680	0.620	0.732	15.644	0.000
Hyde & White, 2009b: registering behaviour	0.530	0.444	0.606	10.222	0.000
Hyde & White, 2010: registering behaviour	0.800	0.758	0.835	20.138	0.000
Hyde & White, 2013a: registering behaviour, control group	0.877	0.794	0.928	9.538	0.000
Hyde & White, 2013b	0.700	0.615	0.769	11.341	0.000
Hyde, Knowles, & White, 2013/Hyde & Knowles, 2013	0.470	0.368	0.562	8.023	0.000
Jun, 1993	0.330	0.191	0.456	4.509	0.000
Jun, 1993	0.305	0.244	0.364	9.361	0.000
Jun, 1993	0.203	0.128	0.275	5.255	0.000
Kashif & De Run, 2015	0.390	0.273	0.496	6.108	0.000
Knowles, Hyde, & White, 2012	0.500	0.391	0.595	7.903	0.000
Lemmens et al., 2005	0.500	0.407	0.582	9.208	0.000
Lemmens et al., 2009: Study 1	0.390	0.279	0.491	6.419	0.000
Lemmens et al., 2009: Study 2	0.460	0.399	0.517	12.921	0.000
Li et al., 2022	0.590	0.540	0.636	18.158	0.000
Mackay, White, & Obst, 2016	0.830	0.791	0.862	20.579	0.000
Masser el al., 2009	0.620	0.520	0.703	9.591	0.000
McMahon & Byrne, 2008	0.360	0.222	0.484	4.900	0.000
Newton et al., 2013	0.590	0.517	0.654	12.660	0.000
O'Brien, Fan, Yi, & Goldman, 2013	0.254	0.172	0.333	5.905	0.000
Poplaski, 2017: main study	0.000	-0.124	0.124	0.000	1.000
Robinson et al., 2008	0.720	0.645	0.781	12.577	0.000
Smith & McSweeney, 2007	0.440	0.329	0.539	7.068	0.000
van der Linden, 2011	0.670	0.568	0.751	9.593	0.000
Veldhuizen & van Dongen, 2013	0.120	-0.012	0.248	1.776	0.076
Veldhuizen & van Dongen, 2013	0.260	0.221	0.298	12.600	0.000
Veldhuizen, Atsma, van Dongen, & de Kort, 2012	0.270	0.253	0.286	30.390	0.000
Veludo-de-Oliveira, Alhaidari, Yani-de-Soriano, & Yousafzai, 2017	0.690	0.637	0.736	17.563	0.000
Veludo-de-Oliveira, Pallister, & Foxall, 2013	0.528	0.429	0.614	8.985	0.000
Warburton & Terry, 2000	0.620	0.544	0.686	12.410	0.000
Zuckerman & Reis, 1978	0.370	0.258	0.472	6.117	0.000
	0.401	0.391	0.411	71.038	0.000

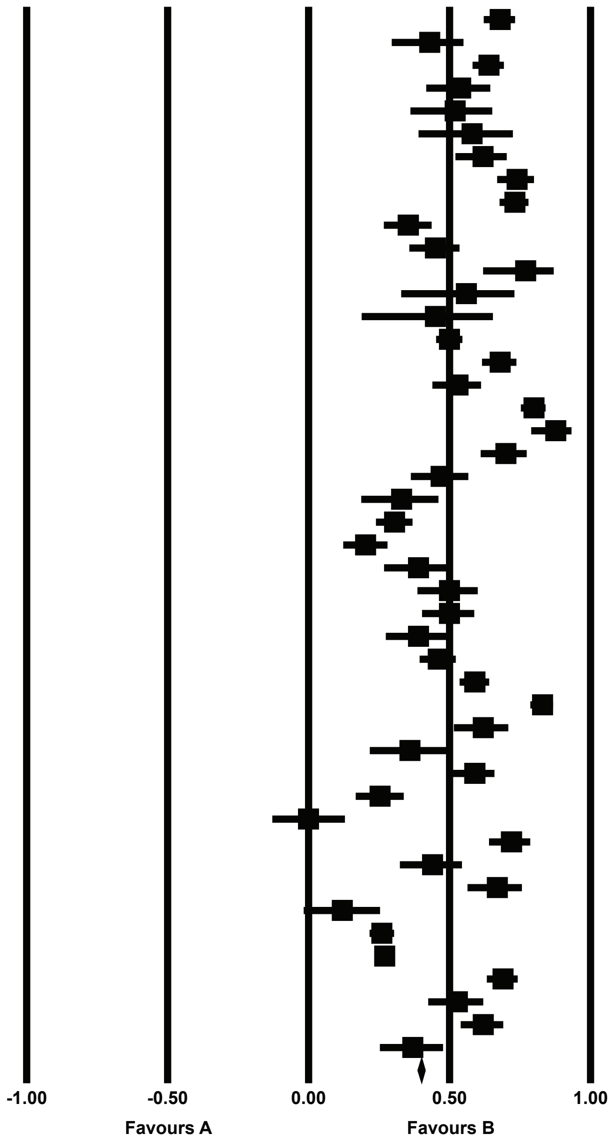

5. Forest plot for the PBC-behaviour association

Study name	Correlation	Lower limit	Upper limit	Z-Value	p-Value
Alhidari, 2014	0.270	0.180	0.355	5.734	0.000
Bagot, Masser, & White, 2015	0.240	0.158	0.319	5.603	0.000
Bagot, Masser, & White, 2015	0.220	0.070	0.360	2.855	0.004
Borgida, Conner, & Manteufel, 1992	0.130	-0.029	0.282	1.607	0.108
Chen, 2017	0.190	0.055	0.318	2.754	0.006
Conner, Godin, Sheeran, & Germain, 2013	0.287	0.232	0.340	9.816	0.000
Giles & Cairns, 1995	0.550	0.425	0.655	7.343	0.000
Giles, McClenahan, Cairns, & Mallet, 2004	0.425	0.172	0.625	3.176	0.001
Godin & Germain, 2013	0.279	0.161	0.389	4.522	0.000
Grano, Lucidi, Zelli, & Violani, 2008	0.230	0.151	0.306	5.596	0.000
Greenslade & White, 2005	0.680	0.542	0.782	7.323	0.000
Holdershaw, 2005: main study	0.290	0.232	0.346	9.465	0.000
Holdershaw, 2005: pilot study	0.270	-0.045	0.536	1.684	0.092
Hu, Wang, & Fu, 2017: Sample 2	0.090	0.023	0.156	2.643	0.008
Hyde & White, 2010: registering behaviour	0.350	0.214	0.473	4.821	0.000
Hyde & White, 2013a: registering behaviour, control group	0.297	0.020	0.531	2.099	0.036
Lu, Cheng, Lin, & Chen, 2019	0.140	0.035	0.242	2.614	0.009
Mackay, White, & Obst, 2016	0.250	0.104	0.386	3.311	0.001
Masser el al., 2009	0.630	0.532	0.711	9.808	0.000
Masser, Bednall, White, & Terry, 2012	0.180	-0.028	0.373	1.697	0.090
O'Brien, Fan, Yi, & Goldman, 2013	0.020	-0.066	0.106	0.455	0.649
Smith & McSweeney, 2007	0.265	0.026	0.475	2.172	0.030
Veludo-de-Oliveira, Alhaidari, Yani-de-Soriano, & Yousafzai, 2017	0.270	0.143	0.388	4.088	0.000
Veludo-de-Oliveira, Pallister, & Foxall, 2013	0.104	-0.052	0.255	1.312	0.190
Warburton & Terry, 2000	0.750	0.689	0.801	14.978	0.000
White, Poulsen, & Hyde, 2017	0.103	-0.097	0.295	1.007	0.314
	0.301	0.227	0.370	7.728	0.000

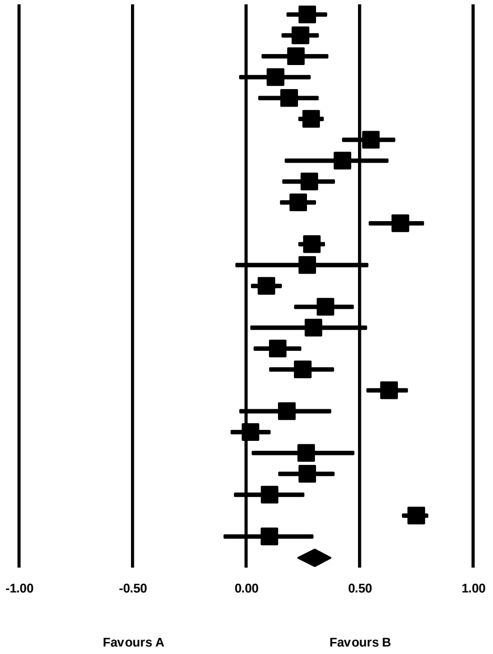

6. Forest plot for the intention-behaviour association

Study name	Correlation	Lower limit	Upper limit	Z-Value	p-Value
	Statistics for each study				
Alhidari, 2014	0.390	0.307	0.467	8.529	0.000
Bagot, Masser, & White, 2015	0.440	0.368	0.506	10.810	0.000
Bagot, Masser, & White, 2015	0.280	0.133	0.415	3.673	0.000
Borgida, Conner, & Manteufel, 1992	0.360	0.214	0.490	4.631	0.000
Charng, Piliavin, & Callero, 1988	0.400	0.334	0.462	10.842	0.000
Chen, 2017	0.570	0.470	0.655	9.271	0.000
Conner, Godin, Sheeran, & Germain, 2013	0.343	0.290	0.394	11.883	0.000
Fortini, 1987	0.573	0.447	0.676	7.491	0.000
Giles & Cairns, 1995	0.750	0.668	0.814	11.553	0.000
Giles, McClenahan, Cairns, & Mallet, 2004	0.480	0.238	0.666	3.661	0.000
Godin & Germain, 2013	0.348	0.235	0.452	5.731	0.000
Grano, Lucidi, Zelli, & Violani, 2008	0.290	0.213	0.363	7.134	0.000
Greenslade & White, 2005	0.750	0.636	0.832	8.593	0.000
Harrison, 1995	0.765	0.616	0.861	6.838	0.000
Harrison, 1995	0.755	0.603	0.854	6.749	0.000
Harrison, 1995	0.800	0.669	0.883	7.451	0.000
Holdershaw, 2005: main study	0.320	0.263	0.374	10.514	0.000
Holdershaw, 2005: pilot study	0.290	-0.024	0.552	1.816	0.069
Hu, Wang, & Fu, 2017: Sample 2	0.160	0.094	0.224	4.727	0.000
Hughes, 1984	0.350	0.019	0.612	2.067	0.039
Hughes, 1984	0.000	-0.512	0.512	0.000	1.000
Hughes, 1984	-0.559	-0.808	-0.140	-2.525	0.012
Hyde & White, 2010: registering behaviour	0.410	0.279	0.526	5.746	0.000
Hyde & White, 2013a: registering behaviour, control group	0.453	0.200	0.649	3.349	0.001
Hyde & White, 2013b	0.280	0.081	0.458	2.729	0.006
Jun, 1993	0.445	0.318	0.556	6.293	0.000
Jun, 1993	0.380	0.322	0.435	11.888	0.000
Jun, 1993	0.250	0.177	0.320	6.537	0.000
Lu, Cheng, Lin, & Chen, 2019	0.190	0.086	0.289	3.567	0.000
Mackay, White, & Obst, 2016	0.530	0.413	0.630	7.649	0.000
Masser el al., 2009	0.640	0.544	0.719	10.030	0.000
Masser, Bednall, White, & Terry, 2012	0.400	0.210	0.561	3.952	0.000
McMahon & Byrne, 2008	0.240	0.094	0.376	3.182	0.001
O'Brien, Fan, Yi, & Goldman, 2013	0.030	-0.056	0.116	0.682	0.495
Smith & McSweeney, 2007	0.380	0.154	0.568	3.200	0.001
Veludo-de-Oliveira, Alhaidari, Yani-de-Soriano, & Yousafzai, 2017	0.390	0.272	0.496	6.080	0.000
Veludo-de-Oliveira, Pallister, & Foxall, 2013	0.162	0.008	0.309	2.054	0.040
Warburton & Terry, 2000	0.750	0.689	0.801	14.978	0.000
Weber, Martin, & Corrigan, 2007	0.479	0.396	0.554	9.994	0.000
White, Poulsen, & Hyde, 2017	0.357	0.171	0.519	3.640	0.000
Zuckerman & Reis, 1978	0.400	0.291	0.499	6.672	0.000
	0.424	0.367	0.477	13.299	0.000

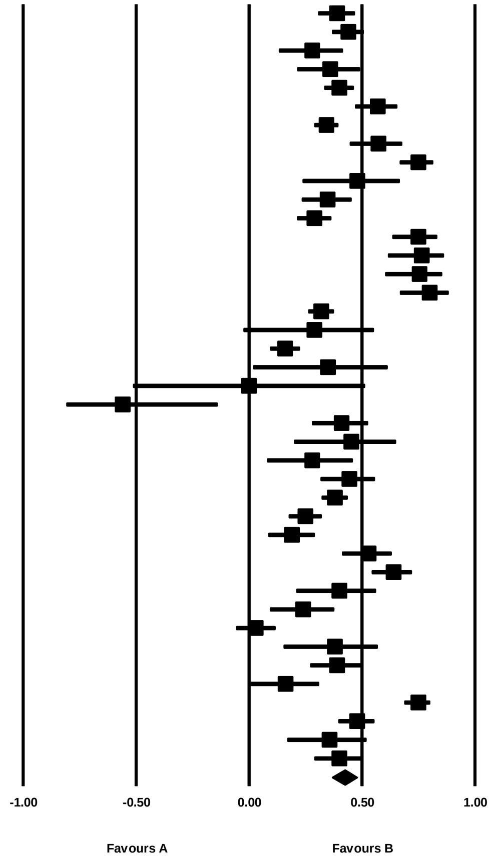

7. Funnel plots of standard error by Fisher's Z

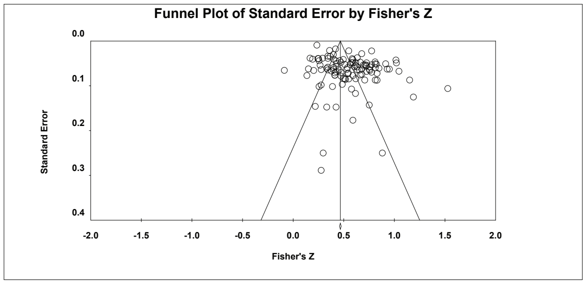

Attitude-intention association

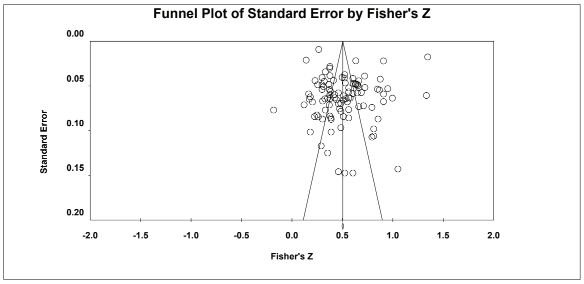

Subjective norm-intention association

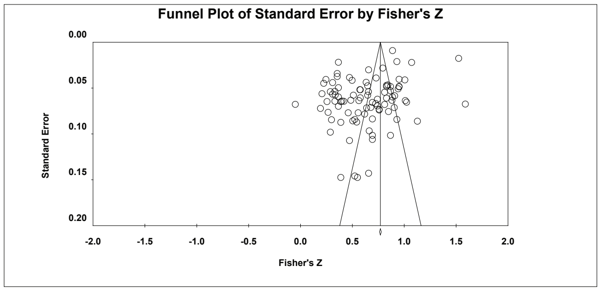

PBC-intention association

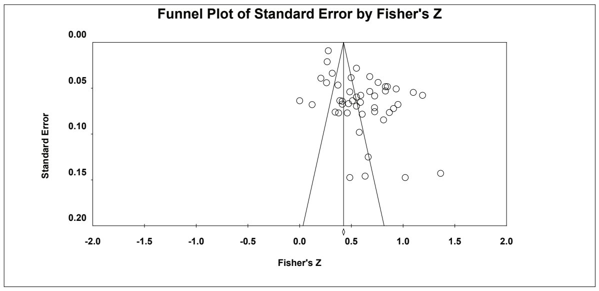

Moral norm-intention association

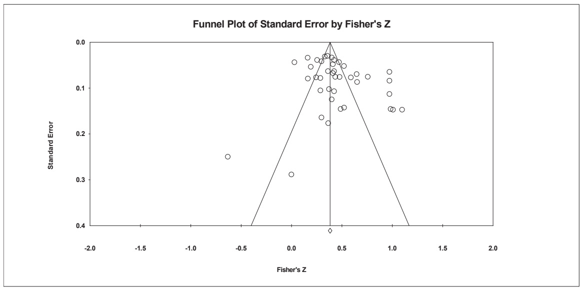

Intention-behaviour association

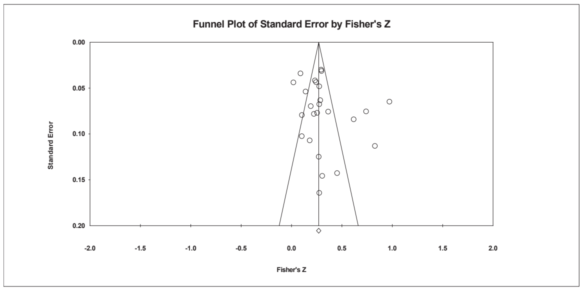

PBC-behaviour association
